# FASN promotes lipid metabolism and progression in colorectal cancer via the SP1/PLA2G4B axis

**DOI:** 10.1038/s41420-025-02409-9

**Published:** 2025-03-28

**Authors:** Xin Liu, Jiachun Lu, Xiangyu Ni, Yuxin He, Jiayu Wang, Zilin Deng, Guangbo Zhang, Tongguo Shi, Weichang Chen

**Affiliations:** 1 Jiangsu Institute of Clinical Immunology, The First Affiliated Hospital of Soochow University, Suzhou, China; 2 Department of Gastroenterology, The First Affiliated Hospital of Soochow University, Suzhou, China; 3 Jiangsu Key Laboratory of Clinical Immunology, Soochow University, Suzhou, China

**Keywords:** Colorectal cancer, Lipid signalling

## Abstract

Abnormal metabolic reprogramming is essential for tumorigenesis, metastasis, and the regulation of immune responses. Fatty acid synthase (FASN), a key enzyme in lipid metabolism, plays a crucial role in these processes. However, the relationship between FASN-mediated lipid reprogramming and the immune response in colorectal cancer (CRC) remains unclear. The present study demonstrated that FASN expression is elevated in CRC tissues and is significantly associated with poor prognosis. Functional experiments revealed that FASN promotes proliferation, migration, invasion, and phosphatidylcholine (PC) production in CRC cells. Additionally, in vivo experiments revealed that FASN knockdown significantly inhibits tumor growth and the spread of CRC cells to the lungs. Mechanistically, FASN, which is upregulated in CRC tissues, drives cancer cell proliferation, metastasis, and PC metabolism through the SP1/PLA2G4B axis, subsequently suppressing the antitumor response of natural killer (NK) cells in a PC-dependent manner. These findings provide new insights into lipid metabolism and the immunobiology of CRC, suggesting potential targets for the treatment and prevention of CRC.

**Figure:**
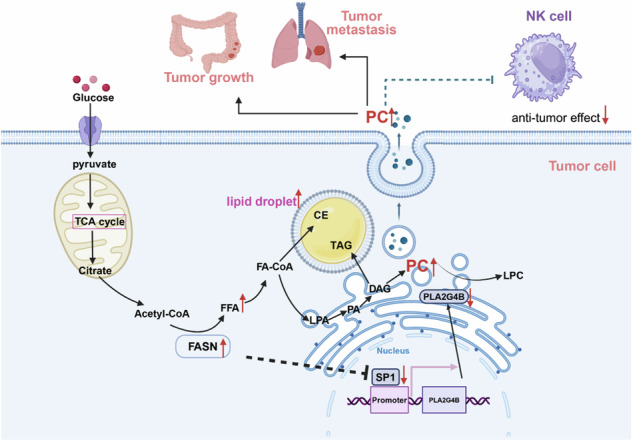
Schematic diagram showing the mechanism by which FASN promotes cancer cell proliferation, metastasis, and PC metabolism in CRC via the SP1/PLA2G4B axis, subsequently suppressing the antitumor response of NK cells in a PC-dependent manner. FFA free fatty acid, LPA lysophosphatidic acid, PA phosphatidate, DAG diglyceride, PC phosphatidylcholine, LPC lysophosphatidylcholine, CE cholesterol ester, TAG triacylglycerol.

Schematic diagram showing the mechanism by which FASN promotes cancer cell proliferation, metastasis, and PC metabolism in CRC via the SP1/PLA2G4B axis, subsequently suppressing the antitumor response of NK cells in a PC-dependent manner. FFA free fatty acid, LPA lysophosphatidic acid, PA phosphatidate, DAG diglyceride, PC phosphatidylcholine, LPC lysophosphatidylcholine, CE cholesterol ester, TAG triacylglycerol.

## Background

Colorectal cancer (CRC) ranks as the third most prevalent malignancy and is the second leading cause of cancer-related death globally [1, 2]. CRC pathogenesis is complex and involves multiple factors, such as gene mutations, intestinal microbial disorders, and metabolic disorders [3, 4]. Rapid tumor growth often results in a nutrient-poor tumor microenvironment (TME), compelling cancer cells to undergo metabolic reprogramming to adapt to these energy constraints [5]. An increasing body of evidence highlights that such aberrant metabolic reprogramming is crucial for the initiation, growth, and metastasis of CRC [6].

Lipid metabolism, a key metabolic signature of cancer cells, has been extensively implicated in cancer initiation, progression, and drug resistance [7]. Many studies have revealed that dynamic changes in lipids, such as triacylglycerol, glycerolipids, sphingolipids, ceramides, and cholesterol, affect the occurrence and progression of various cancers, including CRC [8–10]. Dysregulation of lipid metabolic enzymes, such as fatty acid synthase (FASN) and acyl-CoA oxidase 1, results in lipid metabolism reprogramming in cancers [11]. FASN, a key enzyme in lipid metabolism, catalyzes the de novo synthesis of fatty acids, which supports cell growth and survival [12]. The oncogenic role of FASN in lipid metabolism reprogramming has been well documented across various tumors [13–15]. For example, FASN facilitates lymph node metastasis in cervical cancer by modulating cholesterol reprogramming and then activating the lipid raft-related c-Src/AKT/FAK signaling pathway [16]. FASN, stabilized by ACAT1-mediated GNPAT acetylation, contributes to lipid metabolism reprogramming and hepatocarcinogenesis [17]. However, little is known about the mechanism of FASN in lipid metabolism reprogramming in CRC.

Increasing evidence has indicated that lipid metabolism dysregulation in the TME has the ability to modulate the immune response [18, 19]. Yi et al. reported that polymerase 1 and transcript release factor triggers a cytoplasmic phospholipase A2-mediated phospholipid remodeling pathway, leading to the promotion of tumor proliferation and the inhibition of immune responses in glioblastoma [20]. Cook et al. reported that the silencing of GRP78, an endoplasmic reticulum stress protein, upregulates the intracellular concentrations of essential polyunsaturated fats, including linoleic acid, which further controls tamoxifen sensitivity and antitumor immunity in breast cancer [21]. However, the relationship between FASN-mediated lipid metabolism reprogramming and the immune response in CRC remains unknown.

In the present study, in vitro and in vivo experiments were performed to investigate the roles and molecular mechanisms of FASN in lipid metabolism reprogramming, tumor progression, and the immune response in CRC.

## Results

### Identification of differential lipids in CRC patients

**Fig. 1:**
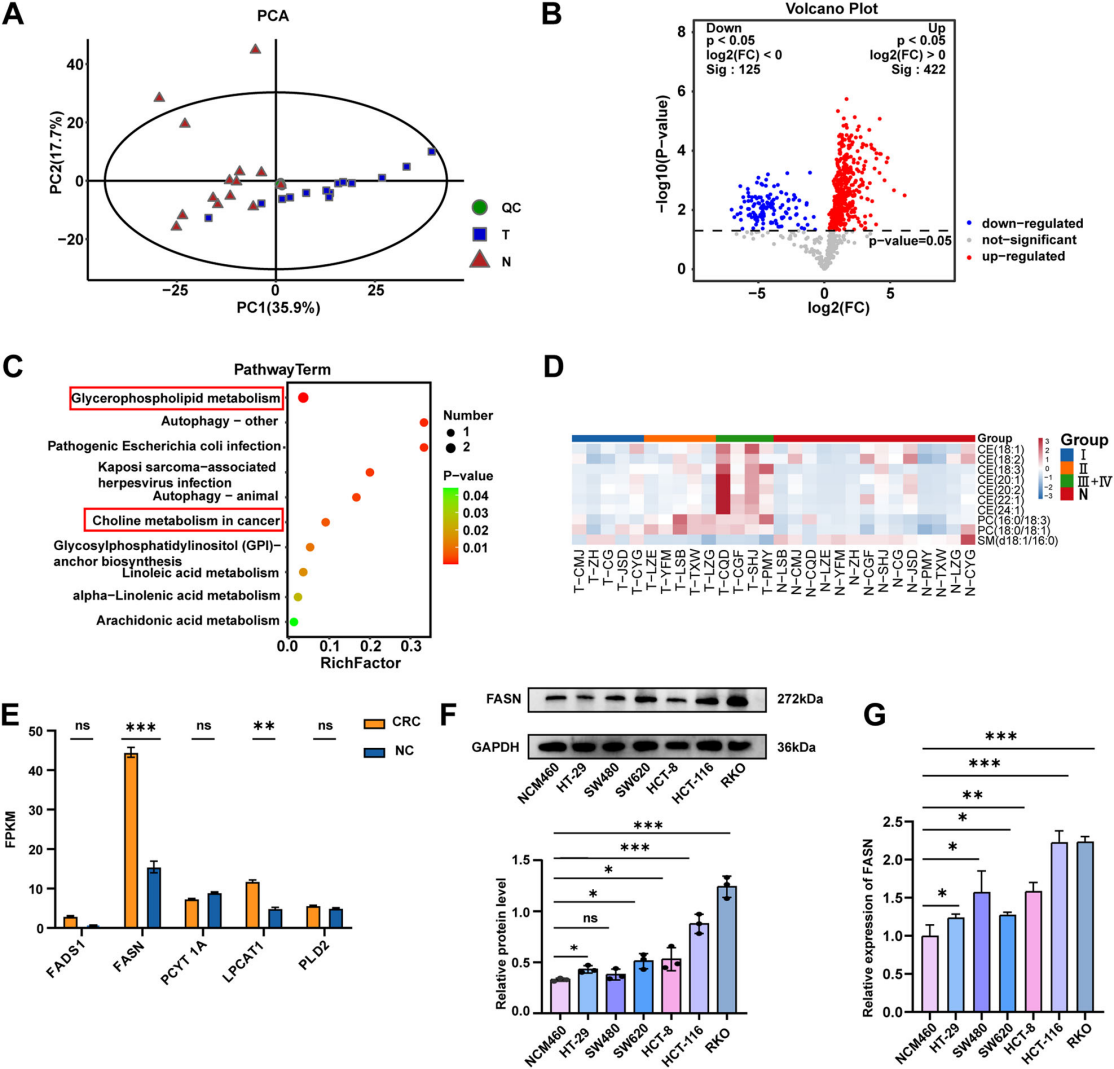
Identification of differential lipids in CRC patients. **A** PCA revealed distinct lipidomic profiles between the cancerous and nontumor tissues of CRC patients. **B** Volcano plot of differentially expressed lipids in cancerous tissues compared with adjacent nontumor tissues. **C** KEGG pathway enrichment analysis of these differentially metabolized lipid concentration pathways. **D** Heatmaps of differential lipids associated with TNM stage. **E** Expression of key enzyme-encoding genes, including FADS1, FASN, PCYT1A, LPCAT1, and PLD2, in patients with CRC in the TCGA database. **F** Western blot analysis was used to measure FASN protein expression in NCM460, HCT-8, HCT-116, HT-29, RKO, SW480, and SW620 cells. GAPDH was used as a loading control. The densities of Western blot bands were quantified using the ImageJ program. **G** The mRNA expression level of FASN in NCM460, HCT-8, HCT-116, HT-29, RKO, SW480, and SW620 cells was detected by RT-qPCR. The experiments were conducted in triplicate. Nonsignificant results are denoted as “ns”, while significance levels are shown as **P < 0.01 and ***P < 0.001.

Cancerous tissue (tumor tissue, T) and adjacent nontumor tissue (normal tissue, N) were collected from 14 CRC patients to perform targeted lipidomics with high coverage (Table S1). Principal component analysis (PCA) revealed distinct lipidomic profiles between the cancerous and nontumor tissues (Fig. 1A). A total of 547 differentially expressed lipids were identified, with 422 upregulated and 125 downregulated in the cancerous tissues compared with the adjacent nontumor tissues (Fig. 1B, Table S2). KEGG pathway enrichment analysis indicated that these differentially metabolized lipids were predominantly involved in glycerophospholipid metabolism and choline metabolism pathways (Fig. 1C). On the basis of their TNM stage, CRC patients were further categorized into phase 1 (stage I), phase 2 (stage II), and phase 3 (stage III + IV) groups. Differential lipids associated with the TNM stage were identified, comprising the following 10 lipids: 7 cholesteryl esters, 2 phosphatidylcholines (PCs), and 1 sphingomyelin (Fig. 1D). Further analyses focused on PCs, which are involved in glycerophospholipid metabolism. We analyzed the total PC level in samples from 14 CRC patients. Tumor tissues exhibited a marked increase in PC levels compared to adjacent normal tissues, and PC levels were positively correlated with TNM stage (Fig. S1A, B). Next, the key enzymes affecting PC metabolism in CRC were investigated. A review of the literature on PC metabolism in various cancers revealed that FADS1 [22], FASN [23–25], PCYT1A [26], LPCAT1 [27–29], and PLD2 [30–32] are involved in PC metabolism in multiple cancers (Table S3). In The Cancer Genome Atlas (TCGA) database, the expression of FASN was greater in CRC patients compared to other key enzyme-encoding genes (Fig. 1E). In addition, the expression levels of FASN in CRC cell lines HCT-8, HCT-116, HT-29, RKO, and SW620 were higher than those in NCM460 intestinal epithelial cell lines (Fig. 1F). We observed that the expression levels of FASN mRNA in CRC cell lines HCT-8, HCT-116, HT-29, RKO, SW480, and SW620 were higher than in normal intestinal epithelial cell lines NCM460 (Fig. 1G). These findings suggest that FASN may be a key gene that potentially regulates PC metabolism in CRC.

### FASN promotes the proliferation, migration, and invasion of CRC cells and the synthesis of PC

**Fig. 2:**
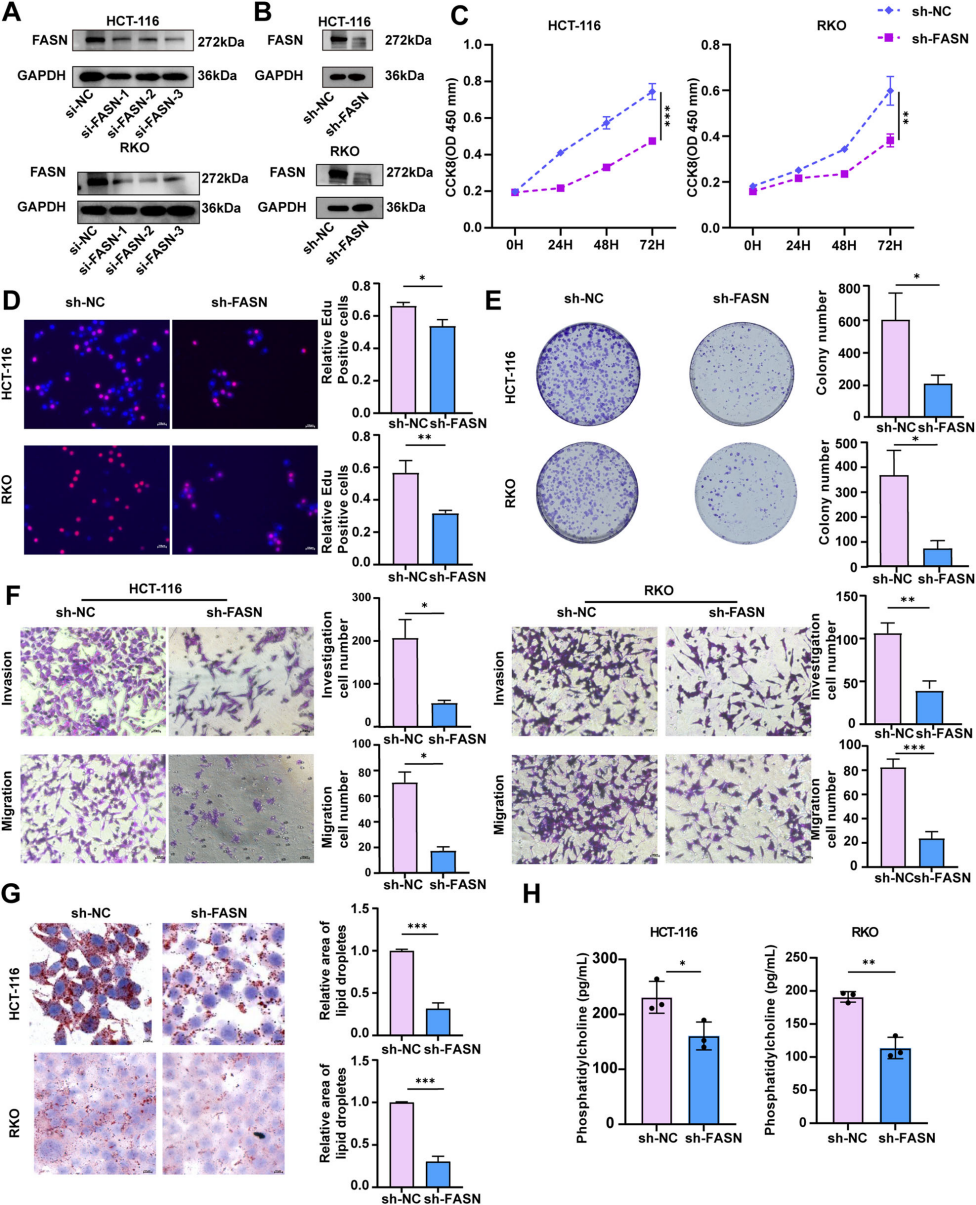
FASN knockdown inhibits the proliferation, migration, and invasion of CRC cells and synthesis of PC. **A** The protein expression of FASN in HCT-116 and RKO cells was examined following transfection with si-FASN-1, si-FASN-2, or si-FASN-3. GAPDH was used as a loading control. **B** FASN protein expression was examined in stable FASN-knockdown HCT-116 and RKO cells. GAPDH was used as a loading control. **C** The proliferation of stable FASN-knockdown HCT-116 and RKO cells was assessed via a CCK-8 assay. **D** EdU analysis was performed to measure the proliferative ability of stable FASN-knockdown HCT-116 and RKO cells. Representative images are presented. Scale bar, 100 μm. The percentage of EdU-positive cells was statistically analyzed and is shown in the bar graph. **E** A colony formation assay was conducted to evaluate the proliferative ability of stable FASN-knockdown HCT-116 and RKO cells. **F** Transwell migration and invasion assays were performed to examine the migratory and invasive capacities of stable FASN-knockdown HCT-116 and RKO cells. **G** Representative images of intracellular lipid droplets in stable FASN-knockdown HCT-116 and RKO cells stained with Oil Red O. **H** The expression levels of PC in the cell supernatants of stable FASN-knockdown HCT-116 and RKO cells were quantified via ELISA. The experiments were conducted in triplicate. The data are presented as the means with standard deviations (SDs), and statistical significance was assessed via Student’s *t*-test. Significance levels are shown as *P < 0.05, **P < 0.01, and ***P < 0.001.

To gain a deeper understanding of the biological role of FASN in CRC, FASN expression was suppressed in RKO and HCT-116 cells. As shown in Figs. 2A and S2A, three small interfering RNAs (siRNAs) targeting FASN (si-FASN-001, si-FASN -002, and si-FASN-003) significantly reduced mRNA levels and protein expression of FASN in HCT-116 and RKO cells. Because si-FASN-001 had the greatest inhibitory effect on FASN expression in CRC cells, the sequence of si-FASN-001 was used to generate a FASN shRNA (sh-FASN) lentivirus and construct stable FASN-knockdown HCT-116 and RKO cells (Fig. 2B). We observed that si-FASN-001, si-FASN -002 and sh-FASN significantly decreased the proliferation of HCT-116 and RKO cells (Figs. 2C–E and S2B–D). Transwell assays confirmed that FASN downregulation inhibited the migratory and invasive capabilities of RKO and HCT-116 cells (Figs. 2F and S2E). Given that PC is a quantitatively important component of lipid droplets [33], Oil Red O staining was used to detect and visualize the lipid droplet content in cells. FASN knockdown significantly decreased the lipid droplet content in HCT-116 and RKO cells (Figs. 2G and S2F). Moreover, Enzyme-linked immunosorbent assay (ELISA) revealed a reduction in PC levels in the cell supernatants of HCT-116 and RKO cells after FASN knockdown (Figs. 2H and S2G).

**Fig. 3:**
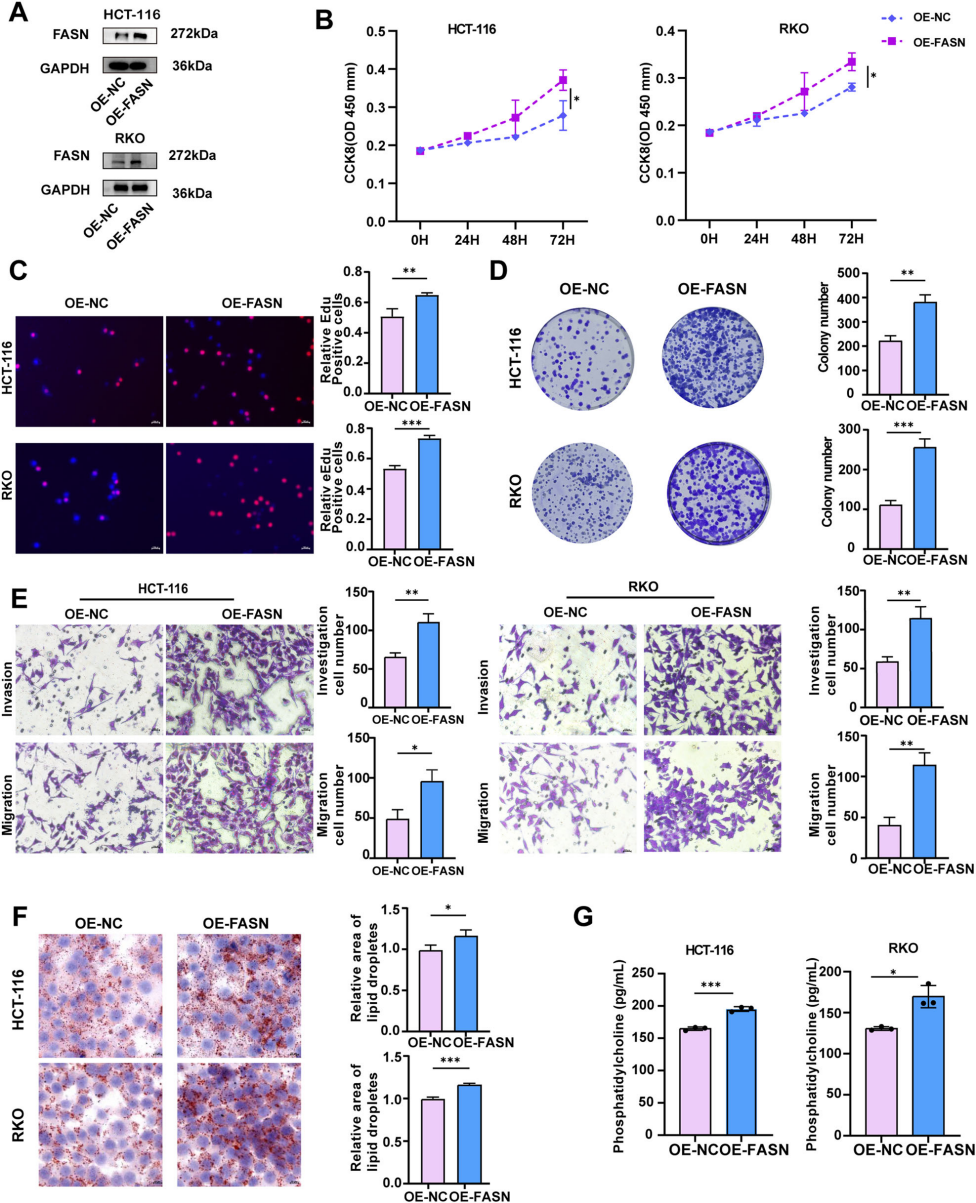
FASN overexpression promotes the proliferation, migration, and invasion of CRC cells and the synthesis of PC. **A** The protein expression of FASN was examined in stable FASN-overexpressing HCT-116 and RKO cells. GAPDH was used as a loading control. **B** The proliferation of stable FASN-overexpressing HCT-116 and RKO cells was assessed via a CCK-8 assay. **C** EdU analysis was performed to measure the proliferative ability of stable FASN-overexpressing HCT-116 and RKO cells. Representative images are presented. Scale bar, 100 μm. The percentage of EdU-positive cells was statistically analyzed and is shown in the bar graph. **D** A colony formation assay was conducted to evaluate the proliferative ability of stable FASN-overexpressing HCT-116 and RKO cells. **E** Transwell migration and invasion assays were performed to examine the migratory and invasive capacities of stable FASN-overexpressing HCT-116 and RKO cells. **F** Representative images of intracellular lipid droplets in stable FASN-overexpressing HCT-116 and RKO cells stained with Oil Red O. **G** The expression level of PC in the cell supernatants of stable FASN-overexpressing HCT-116 and RKO cells was quantified via ELISA. The experiments were conducted in triplicate. The data are presented as the means with standard deviations (SDs), and statistical significance was assessed via Student’s *t*-test. Significance levels are shown as *P < 0.05, **P < 0.01, and ***P < 0.001.

Complementary loss-of-function studies were conducted to investigate the biological significance of FASN in CRC cells. As shown in Fig. 3A, the protein levels of FASN were significantly increased in stable FASN-overexpressing HCT-116 and RKO cells. The CCK-8, colony formation, and EdU assays demonstrated that FASN overexpression significantly increased the proliferation rate of HCT-116 and RKO cells (Fig. 3B–D). Additionally, FASN overexpression enhanced the migration and invasion of HCT-116 and RKO cells (Fig. 3E), as well as significantly increased the levels of lipid droplets and PCs in HCT-116 and RKO cells (Fig. 3F, G).

### FASN promotes the growth and metastasis of CRC cells in vivo

**Fig. 4:**
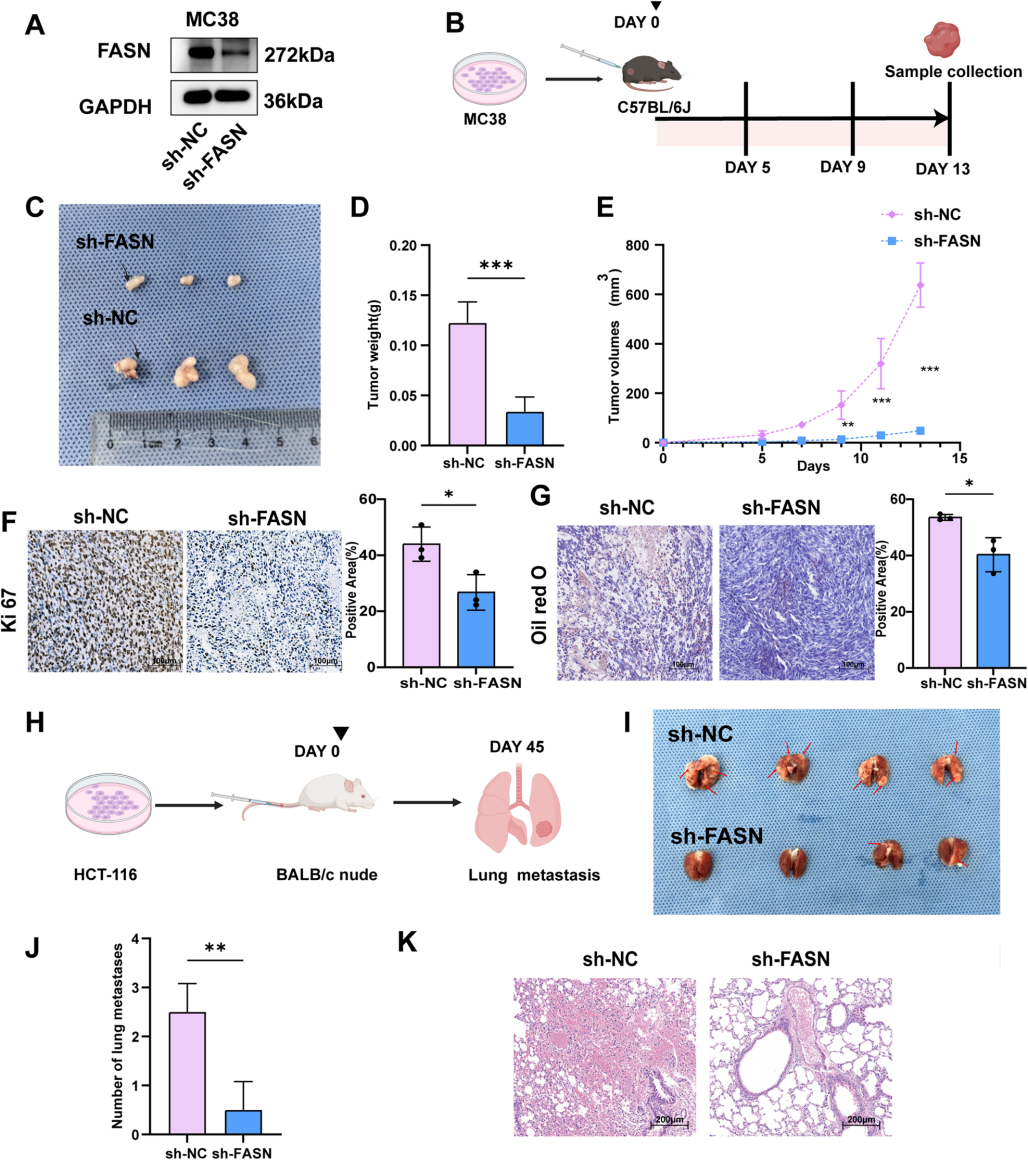
FASN promotes the growth and metastasis of CRC cells in vivo. **A** FASN protein expression was examined in stable FASN knockdown MC38 cells. **B** Schematic diagram of the mouse xenograft tumor model. **C** The image displayed is representative of subcutaneous xenografts from the sh-NC (*n* = 3) and sh-FASN groups (*n* = 3). **D** The weights of subcutaneous xenografts from the sh-NC and sh-FASN groups. **E** The volumes of subcutaneous xenografts from the sh-NC and sh-FASN groups. **F** Representative IHC images of Ki-67 in tumor tissues from the sh-NC and sh-FASN groups. **G** Representative images of intracellular lipid droplets in tumor tissues from the sh-NC and sh-FASN groups. **H** Schematic diagram of the mouse lung metastasis model. **I**, **J** The effects of FASN knockdown on the metastasis of HCT-116 cells in vivo. Representative images of Lungs were observed for metastatic nodules on the surface (**I**). The number of metastatic nodules was statistically analyzed and was shown in the bar graph (**J**). **K** The metastatic nodules were stained by H&E for histological analyses. Scale bar, 200 μm. The data are presented as the means with standard deviations (SDs), and statistical significance was assessed via Student’s *t*-test. Significance levels are shown as **P < 0.01, and ***P < 0.001.

A murine CRC tumor model was established via subcutaneous implantation of FASN-deficient MC38 cells (Fig. 4A, B). Measurement of tumor volume and weight revealed that FASN knockdown inhibited tumor growth in vivo (Fig. 4C–E). Moreover, the expression of Ki-67 was lower in the FASN knockdown group than in the control group (Fig. 4F). Moreover, Oil Red O staining showed that the levels of lipid droplets in the tumor tissues from sh-FASN group were lower than those in the tumor tissues from sh-NC group (Fig. 4G).

To explore the influence of FASN on CRC metastasis in vivo, a mouse lung metastasis model was established (Fig. 4H). As shown in Fig. 4I–K, FASN knockdown significantly reduced the metastatic potential of HCT-116 cells in the lungs of the mice.

### FASN regulates malignant phenotypes and PC Metabolism through PLA2G4B

**Fig. 5:**
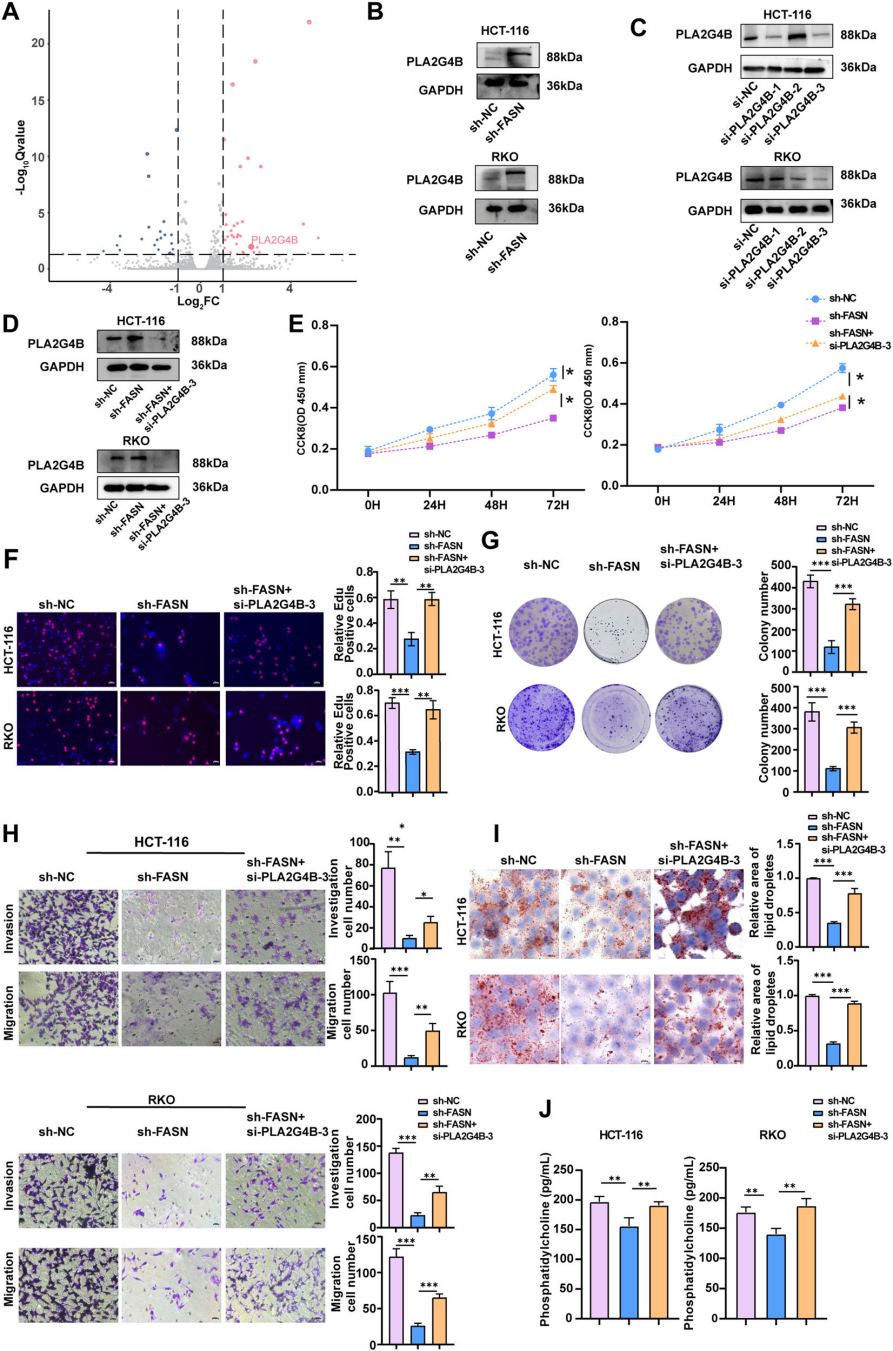
FASN regulates malignant phenotypes and PC metabolism through PLA2G4B. **A** Volcano plot of differentially expressed genes (DEGs) in RKO cells and FASN-knockdown RKO cells. **B** The protein levels of PLA2G4B were measured in stable FASN-knockdown HCT-116 and RKO cells. GAPDH was used as a loading control. **C** The protein expression of PLA2G4B in HCT-116 and RKO cells transfected with si-PLA2G4B-1, si-PLA2G4B-2, or si-PLA2G4B-3 was examined. GAPDH was used as a loading control. **D** The protein levels of PLA2G4B were assessed in stable FASN-knockdown HCT-116 and RKO cells treated with si-PLA2G4B-3. GAPDH was used as a loading control. **E** The proliferation of stable FASN knockdown HCT-116 and RKO cells treated with si-PLA2G4B-3 was evaluated via a CCK-8 assay. **F** EdU analysis was conducted on stable FASN-knockdown HCT-116 and RKO cells treated with si-PLA2G4B-3. **G** Colony formation assays were conducted to evaluate the proliferative ability of stable FASN-knockdown HCT-116 and RKO cells treated with si-PLA2G4B-3. **H** Transwell migration and invasion assays were performed to examine the migratory and invasive capacities of stable FASN-knockdown HCT-116 and RKO cells treated with si-PLA2G4B-3. **I** Representative images of intracellular lipid droplets in stable FASN-knockdown HCT-116 and RKO cells treated with si-PLA2G4B-3 and stained with Oil Red O. **J** The expression levels of PC in the cell supernatants of stable FASN-knockdown HCT-116 and RKO cells treated with si-PLA2G4B-3 were quantified via ELISA. The experiments were conducted in triplicate. The data are presented as the means with standard deviations (SDs), and statistical significance was assessed via Student’s *t*-test. Significance levels are shown as *P < 0.05, **P < 0.01, and ***P < 0.001.

To explore the mechanisms underlying the regulation of the malignant phenotype and PC metabolism of CRC by FASN, RNA-seq analysis was performed on sh-NC and sh-FASN RKO cells. The RNA-seq results revealed 64 DEGs (*Q* value < 0.05 and |log2(foldchange)| > 1) in FASN-deficient, including 37 upregulated genes and 27 downregulated genes (Fig. 5A, Table S4). Among the downregulated DEGs, PLA2G4B is a key enzyme-encoding gene involved in PC metabolism. Analysis of TCGA databased revealed an inverse correlation between FASN and PLA2G4B mRNA expression in CRC (Fig. S3). This negative association between FASN and PLA2G4B was confirmed by Western blot analysis (Fig. 5B). These results suggested that FASN inhibits PLA2G4B expression in CRC.

To explore whether FASN contributes to CRC cell malignant phenotypes and PC metabolism via PLA2G4B, PLA2G4B expression was silenced in HCT-116 and RKO cells using three commercial PLA2G4B siRNAs. si-PLA2G4B-003 had the highest inhibitory effect (Fig. 5C). As shown in Fig. 5D, transfection with si-PLA2G4B-003 reversed the ability of FASN knockdown to promote PLA2G4B expression in CRC cells. Additionally, the deregulation of PLA2G4B counteracted the effects of FASN knockdown on the proliferation, migration, and invasion of RKO and HCT-116 cells (Fig. 5E–H). Furthermore, transfection of sh-FASN cells with si-PLA2G4B resulted in increased lipid droplet content and PC production in CRC cells (Fig. 5I, J).

### FASN Inhibits PLA2G4B Expression Through SP1

**Fig. 6:**
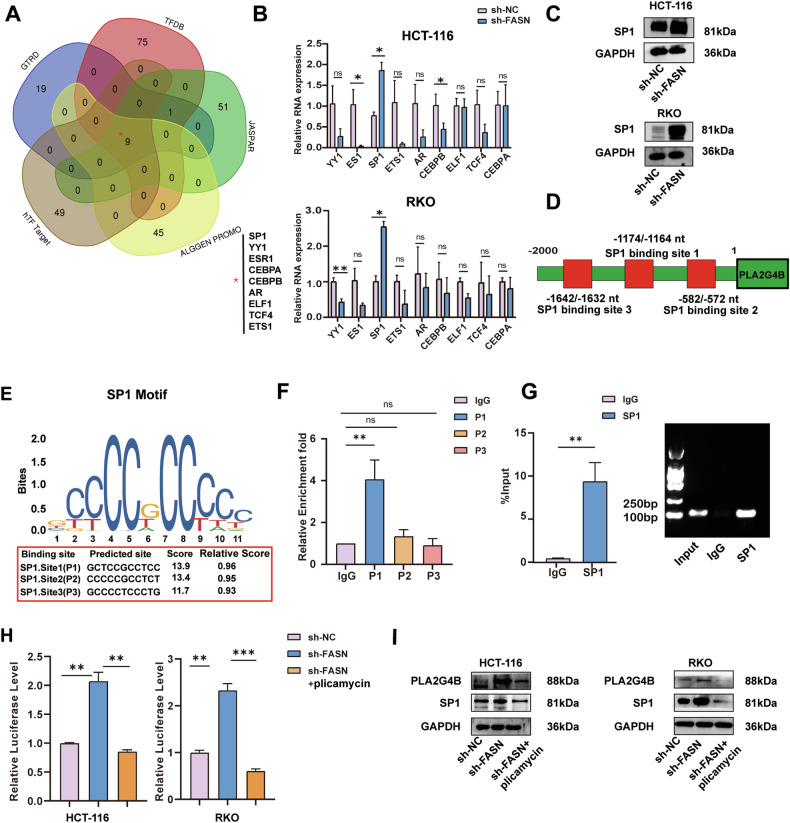
FASN inhibits PLA2G4B expression via SP1. **A** Intersection of PLA2G4B transcription factors was predicted by five databases (JASPAR, TFDB, hTFTarget, ALGGEN PROMO, and GTRD databases). **B** The expression of YY1, SP1, AR, ESR1, ELF1, TCF4, ETS1, CEBPB, and CEBPA was analyzed via qRT‒PCR in stable FASN-knockdown HCT-116 and RKO cells. **C** Western blot analysis was performed to evaluate the expression of SP1 in stable FASN-knockdown CRC cells. GAPDH was used as the control **D**, **E** The potential binding sites of SP1 in the PLA2G4B promoter region were predicted via the JASPAR website, which revealed three binding sites for SP1, namely, P1, P2, and P3. Cut & Run (**F**) and ChIP (**G**) analyses were conducted to assess the binding of SP1 to the PLA2G4B promoter in HCT-116 cells. **H** Luciferase activity, which is responsive to SP1, was measured in FASN-knockdown CRC cells treated with plicamycin. **I** Western blot analysis was performed to evaluate the expression of PLA2G4B and SP1 in stable FASN-knockdown CRC cells treated with plicamycin. GAPDH was used as the control. The experiments were conducted in triplicate. The data are presented as the means with standard deviations (SDs), and statistical significance was assessed via Student’s *t*-test. Nonsignificant results are denoted as “ns”, while significance levels are shown as *P < 0.05, **P < 0.01, and ***P < 0.001.

To examine the regulatory role of FASN in the regulation of PLA2G4B expression in CRC cells, the JASPAR, TFDB, hTFTarget, ALGGEN PROMO, and GTRD databases were used to predict transcription factors that bind to the promoter region of PLA2G4B. As shown in Fig. 6A, nine putative transcription factors, namely, YY1, SP1, AR, ESR1, ELF1, TCF4, ETS1, CEBPB, and CEBPA, were identified. RNA extraction and quantitative real-time PCR (qRT‒PCR) and Western blot analysis revealed a negative correlation between FASN and SP1 in both HCT-116 and RKO cells (Fig. 6B, C), suggesting that FASN may modulate PLA2G4B expression via SP1. The JASPAR website was used to predict the potential binding sites of SP1 in the PLA2G4B promoter region, which revealed three binding sites for SP1, namely, P1(−1174 to −1164 nt), P2(−582 to −572 nt), and P3(−1642 to −1632 nt) (Fig. 6D, E). The cleavage under targets and release using nuclease (Cut & Run) and chromatin immunoprecipitation (ChIP) assay revealed that SP1 bound to the P1 site of the promoter region of PLA2G4B (Fig. 6F, G). Additionally, the luciferase reporter gene assay revealed that FASN knockdown significantly increased the SP1 DNA-binding activity on the PLA2G4B promoter. However, the SP1 inhibitor plicamycin reversed this effect (Fig. 6H). Furthermore, the addition of plicamycin reversed the effect of FASN knockdown on PLA2G4B expression in HCT-116 and RKO cells (Fig. 6I). These results indicated that FASN suppresses PLA2G4B expression in CRC cells by inhibiting SP1 expression.

### FASN suppresses the killing ability of NK cells in a PC-dependent manner

**Fig. 7:**
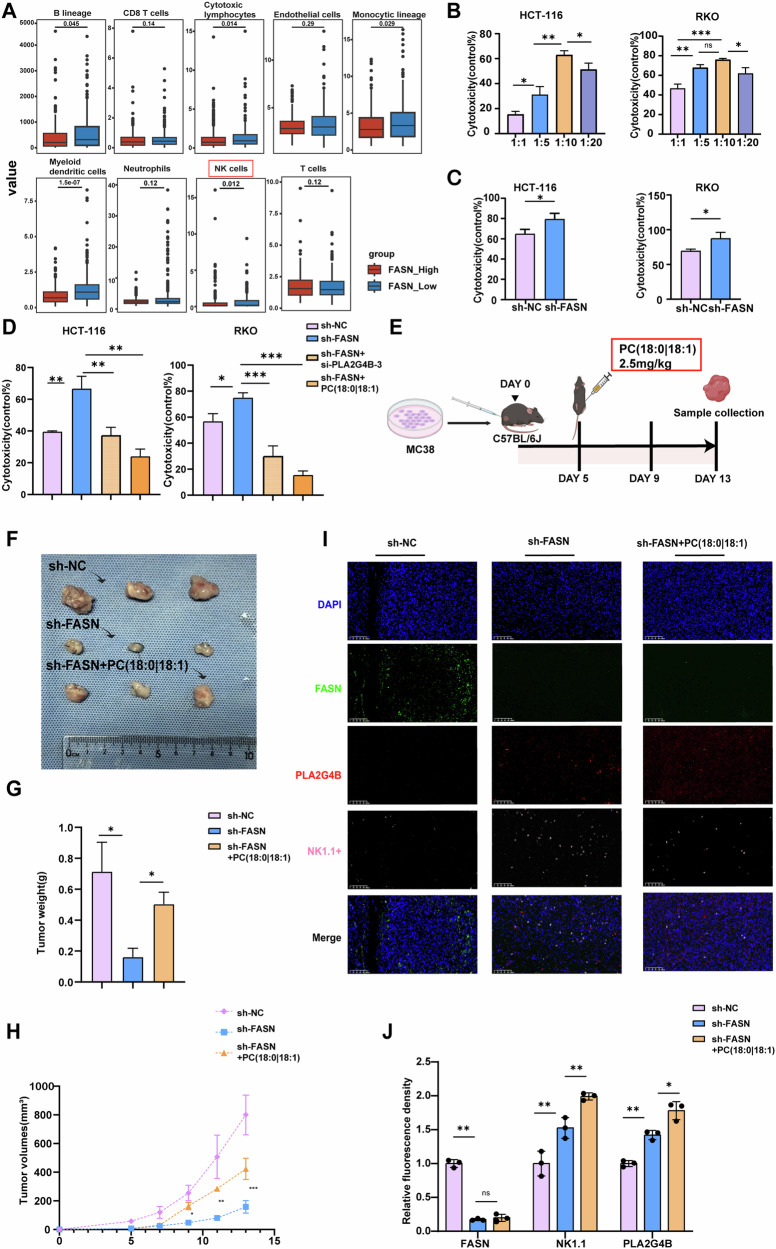
FASN suppresses the killing ability of NK cells in a PC-dependent manner. **A** The distribution of various immune cells in FASN-low and FASN-high CRC tissues. Clinical information for patients with GC was obtained from the TCGA dataset. **B** The cytotoxicity of NK92-MI cells to CRC cells (HCT-116 and RKO cells) at T/E ratios of 1:1, 1:5, 1:10, and 1:20 was evaluated via a CCK-8 assay. **C** The cytotoxicity of NK92-MI cells to FASN-knockdown HCT-116 or RKO cells at T/E ratios of 1:10 was evaluated via a CCK-8 assay. **D** The cytotoxicity of NK92-MI cells to FASN-knockdown HCT-116 or RKO cells transferred with si-PLA2G4B-3 or treated with PC (18:0|18:1) at a concentration of 5 µmol/L was evaluated via a CCK-8 assay. **E** Schematic diagram of C57BL/6J mice bearing established sh-NC, sh-FASN, or sh-FASN tumors injected with PC (18:0|18:1) at 2.5 mg/kg intratumorally at a given time. The representative tumor images (**F**), tumor weights (**G**), and tumor volumes (**H**) of mice harboring sh-NC-, sh-FASN-, or sh-FASN tumors were injected with PC (18:0|18:1). **I**, **J** Effect of PC administration (18:0|18:1) on the number of NK cells in tumor tissues with FASN knockdown. The experiments were conducted in triplicate. The data are presented as the means with standard deviations (SDs), and statistical significance was assessed via Student’s *t*-test. Nonsignificant results are denoted as “ns”, while significance levels are shown as *P < 0.05, **P < 0.01, and ***P < 0.001.

Increasing evidence has shown that PC plays a pivotal role in the communication between cancer cells and immune cells within the TME [34]. Additionally, the present results indicated the PC levels in the cell supernatants of HCT-116 and RKO cells were decreased by FASN knockdown but increased by FASN overexpression (Figs. 2H and 3G). Thus, FASN may regulate the immune response in the TME via PC. To verify this hypothesis, the correlation between FASN expression and immune cell infiltration in CRC was assessed via MCP-counter, which revealed a negative correlation between high FASN expression and the natural killer (NK) immunocytotoxicity score (Fig. 7A). To elucidate the effect of FASN on NK cell cytotoxicity, NK92-MI cells (Effector, E) were cocultured with HCT-116 and RKO cells (Target, T) at T/E ratios of 1:1, 1:5, 1:10, and 1:20. As shown in Fig. 7B, the highest cytotoxic effect of NK92-MI cells on HCT-116 or RKO cells was observed at a T/E ratio of 1:10. Therefore, a T/E ratio of 1:10 was used to examine the effects of FASN on the cytotoxicity of NK92-MI cells. Compared with that in the control group, NK92-MI cell cytotoxicity was significantly increased in FASN-deficient HCT-116 and RKO cells (Fig. 7C).

To further elucidate whether PC is involved in FASN-mediated inhibition of NK cell cytotoxicity, we transfected si-PLA2G4B-3 in sh-FASN and treated CRC cells with exogenous PC (18:0|18:1), respectively. As shown in Fig. 7D, transfection of si-PLA2G4B-3 or treatment with PC (18:0|18:1) at a concentration of 5 µmol/L abolished the effect of FASN knockdown in CRC cells on the cytotoxicity of NK92-MI cells. These results suggest that FASN inhibits the cytotoxic activity of NK cells against CRC cells via PC, warranting further investigation into its underlying mechanism. Additionally, we performed ELISA to quantify the secretion of IFN-γ and granzyme B by NK92mi cells exposed to different treatment conditions (Fig. S4A). The data revealed a significant increase in IFN-γ and granzyme B secretion following FASN knockdown. This increase was partially reversed by transfection with si-PLA2G4B-3 or by the supplementation of exogenous PC (18:0|18:1) (Fig. S4B, C).

Several studies have previously emphasized the critical role of the AKT signaling pathway in regulating NK cell function [35, 36]. Based on these findings, we hypothesized that FASN knockdown might activate the AKT pathway, thereby enhancing NK cell activation. To test this hypothesis, the expression levels of phosphorylated AKT (p-AKT) and total AKT were measured in NK92mi cells treated with conditioned media from CRC cells. The results demonstrated that the AKT activation induced by conditioned media from sh-FASN CRC cells was reversed by conditioned media from FASN knockdown CRC cells transfected with si-PLA2G4B-3 or treated with exogenous PC (18:0|18:1) (Fig. S4D).

The impact of PC (18:0|18:1) was further confirmed in vivo (Fig. 7E). As shown in Fig. 7F–H, intraperitoneal injection of PC (18:0|18:1) reversed the effect of FASN knockdown on MC38 xenograft growth, as evidenced by the tumor image, tumor weight, and tumor volume. Furthermore, the number of NK cells was significantly higher in the tumor tissue from the FASN-deficient group than in that from the control group (Fig. 7I). Administration of PC (18:0|18:1) abolished the influence of FASN knockdown on the number of NK cells in the tumor tissue (Fig. 7I, J). These results indicated that FASN inhibits the ability of NK cells to kill CRC cells via PC.

### FASN expression negatively correlates with PLA2G4B in CRC patients

**Fig. 8:**
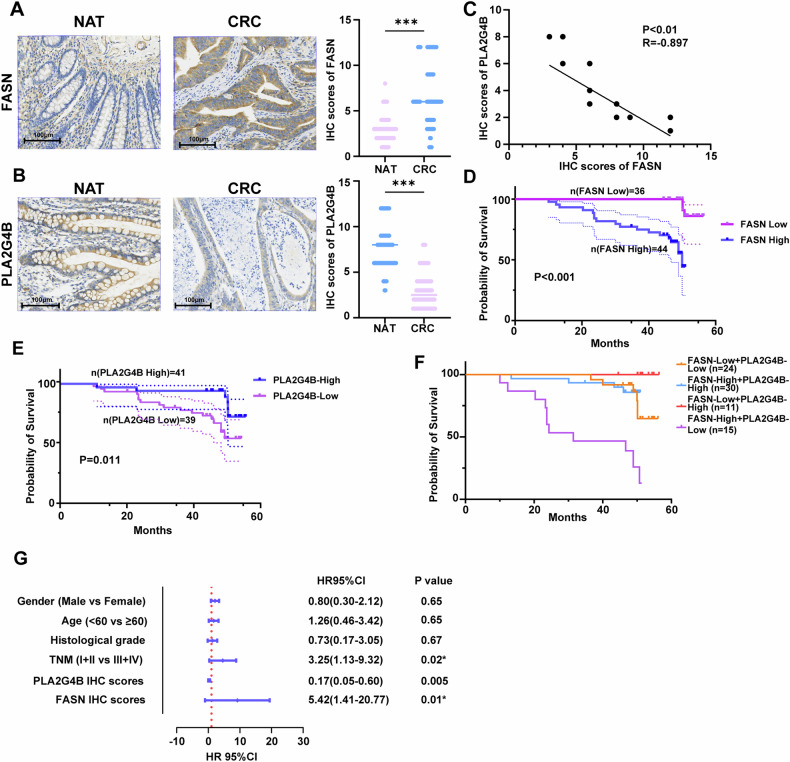
FASN expression is negatively correlated with PLA2G4B in CRC patients. Eighty CRC samples and normal adjacent tissues were subjected to IHC analysis to assess the expression of FASN (**A**) and PLA2G4B (**B**). Representative IHC images showing FASN (**A**) and PLA2G4B (**B**) staining in CRC sections. Scale bar, 100 μm. **C** The correlation between FASN expression and PLA2G4B expression in CRC patients was analyzed. **D** CRC patients with high FASN expression had a significantly poorer prognosis than patients with low FASN expression. **E** CRC patients with high PLA2G4B expression had a significantly better prognosis than patients with low PLA2G4B expression. **F** Kaplan–Meier curves for the overall survival of CRC patients according to the expression of FASN and PLA2G4B in the four patient subtypes. **G** Multivariate Cox regression analysis was conducted to evaluate the association between FASN expression and overall survival, as depicted in the forest plots. Statistical significance was assessed via Student’s *t*-test. Significance levels are shown as ***P < 0.001.

To study the expression of FASN and PLA2G4B, as well as their clinical significance in CRC patients, immunohistochemistry (IHC) was performed, and clinicopathological data were statistically analyzed. Compared with that in normal colon tissues, FASN protein expression was significantly increased in CRC tissues, whereas PLA2G4B expression was significantly decreased in CRC tissues (Fig. 8A, B). Moreover, there was a negative correlation between FASN and PLA2G4B expression in CRC tissues (Fig. 8C). To investigate the clinicopathological characteristics of FASN expression in CRC tissues, CRC patients were divided into a group with high FASN expression (IHC score > 6) and a group with low FASN expression (IHC score ≤ 6). FASN expression was significantly correlated with tumor differentiation and survival (Table S5). Moreover, high FASN expression in CRC patients was associated with poorer overall survival (Fig. 8D). In addition, according to PLA2G4B expression in CRC tissues, CRC patients were divided into a PLA2G4B high-expression group (IHC score > 2) and a PLA2G4B low-expression group (IHC score ≤ 2). PLA2G4B expression was significantly correlated with tumor differentiation and survival (Table S6). K–M analysis revealed that CRC patients expressing high levels of PLA2G4B had higher overall survival (Fig. 8E). Further, the overall survival rate of CRC patients with low FASN expression and high PLA2G4B expression was significantly better than that of patients in the other groups (Fig. 8F). Multivariate Cox regression analysis revealed that TNM stage, high FASN expression, and low PLA2G4B expression were important risk factors for the overall survival of CRC patients (Fig. 8G).

## Discussion

Lipid metabolism reprogramming is a well‐documented phenomenon with great significance in the development and progression of various cancers [11, 37]. With the development of high-throughput lipidomics, specific lipid profiles are increasingly recognized as potential biomarkers for the diagnosis, prognosis, and prediction of diseases [38]. A lipidomic study involving three independent CRC cohorts has revealed alterations in sphingolipid and glycolipid levels, alongside increased expression of lipases in CRC patients [8]. The present study employed LC/MS-based targeted lipidomics using CRC tissue samples, which identified 547 differentially expressed lipids in CRC cancerous tissues. PC, a glycerophospholipid integral to cell membranes and involved in intercellular signaling, has been identified as an early diagnostic marker for several cancers [39, 40]. Lipid mediators derived from PCs, such as prostaglandin E2, platelet-activating factor, and lysophosphatidic acid, are produced by cancer cells and participate in multiple biological processes of cancers [41]. The present study demonstrated that PC was positively associated with the TNM stage in CRC patients, implying that alterations in PC metabolism are associated with CRC progression. Although the diagnostic value and biological functions of lipid mediators derived from PCs in CRC were not explored in the present study, these mediators may be important for CRC diagnosis and progression. Thus, further investigations are needed.

To reveal which key enzymes modulate PC metabolism in CRC, the literature on PC metabolism in various cancers was reviewed, which revealed that FADS1 [22], FASN [23–25], PCYT1A [26], LPCAT1 [27–29], and PLD2 [30–32] regulate PC metabolism in multiple cancers. Among these enzymes, FASN was expressed at the highest level in CRC patients in the TCGA database, suggesting that FASN plays a crucial role in the regulation of PC metabolism in CRC. FASN is a key enzyme in the de novo synthesis of fatty acids and is involved in energy storage, membrane biosynthesis, and the generation of signaling mediators, which are critical for cancer development during tumorigenesis [42]. For example, FASN, which is upregulated in cervical cancer samples, promotes lymph node metastasis via cholesterol reprogramming and lymphangiogenesis [16]. Wu et al. reported that FASN silencing reduces the anoikis resistance of CRC cells and inhibits CRC liver metastases through the ERK1/2 pathway [43]. In the present study, CRC cell proliferation, migration, invasion, and PC metabolism were suppressed by FASN knockdown but enhanced by FASN overexpression. Moreover, FASN silencing reduced tumor growth and lung metastasis in vivo. These results confirmed that FASN acts as a key regulator of cancer cell proliferation, metastasis, and PC metabolism in CRC.

PLA2G4B, a member of the A2 family of cell membrane phospholipases, selectively hydrolyzes glycerophospholipids at the Sn-2 position and preferentially targets arachidonic acid acyl phospholipids [44]. PLA2G4B has been reported to inhibit the breakdown of PC, thereby reducing the production of its derived lipid mediators and slowing the progression of tumors [45]. In the present study, RNA sequencing demonstrated that PLA2G4B was significantly upregulated in FASN-deficient CRC cells. Moreover, PLA2G4B depletion abolished the effects of FASN silencing on CRC cell proliferation, metastasis, and PC metabolism. In addition, there was a negative correlation between FASN and PLA2G4B in CRC tissue specimens, and CRC patients with low FASN expression and high PLA2G4B expression had a better prognosis. These data suggested that PLA2G4B is involved in FASN-mediated modulation of CRC cell proliferation, metastasis, and PC metabolism.

To investigate how FASN regulates PLA2G4B expression in CRC cells, online databases were used to predict transcription factors that regulate PLA2G4B expression. qRT–PCR analysis revealed a negative correlation between FASN and SP1 expression in CRC cells. SP1 is a transcription factor known for its interaction with GC-rich promoter sequences, and it acts as a sequence-specific DNA-binding protein [16, 46] In the present study, Cut & Run and ChIP analyses demonstrated that SP1 could bind to the promoter of PLA2G4B. Moreover, the addition of an SP1 inhibitor abolished the effects of FASN on PLA2G4B expression in CRC cells, as confirmed by luciferase activity assays and Western blot analysis. These findings suggested that FASN regulates PLA2G4B expression in CRC cells through the inhibition of SP1. However, further molecular mechanisms underlying FASN-mediated SP1 modulation deserve extensive study.

Increasing evidence has indicated that tumor cells harness lipid metabolism to influence the activity of stromal and immune cells to their advantage, resist therapy, and promote relapse [6, 47]. Among lipids, PC and its metabolites also play crucial roles in cellular communication between tumor cells and immune cells within the TME [34]. The present study demonstrated that FASN modulated PC metabolism in CRC cells. To investigate whether and how FASN induces an immune response in the TME of CRC via PC, the MCP-counter algorithm was utilized, which revealed that NK cell infiltration was significantly decreased in CRC tissues with high FASN expression, suggesting that FASN may modulate NK cell activity and infiltration in CRC. As cytotoxic lymphocytes, NK cells are involved in viral infection and cancer immunity by killing target cells and producing cytokines [48]. However, the function of tumor-infiltrating NK cells is usually impaired in the immunosuppressive TME [49]. For example, global or myeloid STING knockout leads to a decrease in intrahepatic infiltration and impaired antitumor function of NK cells, exacerbating CRC liver metastasis [50]. In this study, FASN knockdown in CRC cells was found to enhance the cytotoxicity of NK92-MI cells through the activation of the AKT pathway. Furthermore, the effects of FASN knockdown on NK cell cytotoxicity were reversed upon the knockdown of PLA2G4B or the addition of exogenous PC. In vivo experiments also revealed that FASN knockdown increased NK cell infiltration in MC38 xenografts, and the addition of exogenous PC reversed the impact of FASN knockdown on NK cell cytotoxicity and infiltration. These findings suggested that in the TME of CRC, FASN promotes PC metabolism in CRC cells and impairs the cytotoxicity and infiltration of NK cells via PC. Because PC influences other immune cells, including T cells and macrophages [51, 52], FASN-mediated PC metabolism may induce an immune response in CRC through other immune cells.

## Conclusion

In summary, the present study demonstrated that FASN, which is increased in the cancer tissues of CRC patients, promotes cancer cell proliferation, metastasis, and PC metabolism via the SP1/PLA2G4B axis, subsequently suppressing the antitumor response of NK cells in a PC-dependent manner (Graphical Abstract). The present findings not only provide insights into the lipid metabolism and immunobiology of CRC but also highlight promising targets for CRC therapy and prevention.

## Materials and methods

### Lipidomic analysis

Fourteen pairs of CRC tissues and corresponding paracancerous tissues were collected from patients who underwent surgery at the First Affiliated Hospital of Soochow University (Suzhou, China). The patient characteristics are comprehensively outlined in Table S1. The experimental protocol was approved by the Ethics Committee of the First Affiliated Hospital of Soochow University (No. 2024385). Written informed consent was obtained from each patient.

Lipidomic analysis of CRC tissue samples was performed at Shanghai Lumin Biological Technology (Shanghai, China). Briefly, fresh tissue samples were extracted and separated via liquid extraction technology to remove interfering substances. Next, the lipid molecules in the sample were separated via liquid chromatography (LC). The separated lipids were separated by liquid-phase chromatography and subsequently analyzed via a QTRAP® 6500+ tandem mass spectrometer (Sciex, USA) in negative/positive-ion working mode with a time-scheduled multiple reaction monitoring (MRM) method. Finally, the data were analyzed to determine the changes in and correlations among lipid molecules. The lipid internal label information is shown in Table S7.

The raw data files were processed and annotated via MRMPROBS software designed by Tsugawa [53]. PCA and differential lipid analysis were performed using R software (R Foundation for Statistical Computing, Vienna, Austria).

### Cell culture

Human CRC cell lines (NCM460, RKO, HCT-116, HCT-8, HT-29, SW480, and SW620) and mouse MC38 cells (Chinese Academy of Science Cell Bank, Shanghai, China) were cultured in Dulbecco’s Modified Eagle’s Medium (DMEM, EallBio, Beijing, China) supplemented with 10% fetal bovine serum (FBS, Gibco, California, USA) and 1% penicillin‒streptomycin-amphotericin solution (NCM Biotech, Suzhou, China). NK92-MI cells (American Type Culture Collection, ATCC, USA) were cultured in MEM-α medium (EallBio) supplemented with 12.5% FBS, 1% penicillin-streptomycin (NCM Biotech), folic acid (Beyotime, Shanghai, China), β-mercaptoethanol (Sigma‒Aldrich, St. Louis, USA), inositol (Beyotime), and L-glutamine (Gibco). These cells were maintained in a humidified incubator at 37 °C with 5% CO_2_ (Thermo Fisher Scientific, Waltham, USA).

### Cell transfection and lentiviral infection

Three siRNAs targeting mouse FASN (si-FASN-001, si-FASN-002, and si-FASN-003), along with their corresponding negative control (NC) RNAs, were obtained from Suzhou GenePharma (Suzhou, China). Three siRNAs targeting human FASN (si-FASN-001, si-FASN-002, and si-FASN-003) and three siRNAs targeting human PLA2G4B (si-PLA2G4B-001, si-PLA2G4B-002, and si-PLA2G4B-003), along with their corresponding NC RNAs, were obtained from RiboBio (Guangdong, China). The sequences of the above siRNAs are shown in Table S8. FASN and SP1 overexpression plasmids, as well as their respective control plasmids, were acquired from Wuhan Miaoling Biotechnology Company (Wuhan, China). siRNAs or plasmids were transfected via Lipo8000™ Transfection Reagent (Beyotime) according to the manufacturer’s instructions.

The lentiviruses, including one with a short hairpin RNA (shRNA) targeting human FASN using the human si-FASN-001 sequence and one with a shRNA targeting mouse FASN using the mouse si-FASN-003 sequence, were purchased from GenePharma. MC38, HCT-116, and RKO cells were cultured until an exponential growth phase was reached at 40% confluence and were infected with the lentiviral particles at a multiplicity of infection (MOI) of 40.

### RNA extraction and quantitative real-time PCR (qRT–PCR)

Following the manufacturer’s instructions, total RNA was extracted from cells via an RNA-Quick Purification Kit (Yishan, Shanghai, China). One microgram of total RNA from each sample was then used for cDNA synthesis with the HiScript III RT SuperMix Kit (Vazyme, Nanjing, China). Polymerase chain reaction (PCR) assays were conducted on a CFX96 Touch Real-Time PCR system (Bio-Rad, California, USA) under the following conditions: initial denaturation at 95 °C for 5 min, followed by 40 cycles of 95 °C for 10 s and 60 °C for 30 s. The results were analyzed via the 2^−ΔΔCt^ method. The primer sequences designed by GENEWIZ (Suzhou, China) are listed in Table S9.

### Protein extraction and Western blot analysis

Western blot analysis was used to assess protein expression. The cells were lysed via sodium dodecyl sulfate (SDS) lysis buffer (Beyotime) with an ultrasonic cell disruptor on ice at 20% amplitude for 1 min. Total protein concentrations were determined via an Enhanced BCA Protein Assay Kit (Beyotime). Protein samples were then separated on 7.5% SDS-polyacrylamide gels (NCM Biotech) and transferred onto PVDF membranes (GE Healthcare Life Sciences, Pittsburgh, USA). After being blocked with 5% skim milk at room temperature for 1 h, the membranes were incubated with the appropriate primary antibodies at 4 °C overnight. The primary antibodies utilized included an anti-FASN antibody (Proteintech, Wuhan, China), anti-PLA2G4B antibody (Proteintech), anti-SP1 antibody (Proteintech), anti-AKT antibody (Cell Signaling Technology, Danvers, Massachusetts, USA), anti-p-AKT antibody (Cell Signaling Technology), and anti-GAPDH antibody (Proteintech). Horseradish peroxidase (HRP)-conjugated secondary antibodies were subsequently applied for 1 h at room temperature. Bands were visualized via an enhanced chemiluminescence (ECL) reagent (NCM Biotech) and imaged with a ChemiDoc™ MP Imaging System (Bio-Rad).

### CCK-8 assay

Cell proliferation was assessed via a Cell Counting Kit-8 (CCK-8; NCM Biotech). CRC cells were seeded in a 96-well plate at a density of 2000 cells per well. Then, CCK-8 reagent (10 µL) was added to each well at 0, 24, 48, and 72 h. After the plates were incubated at 37 °C for 2 h, the absorbance (OD value) was measured at a wavelength of 450 nm.

### EdU incorporation assay

The cells were cultured in a 24-well plate overnight to prepare for the EdU incorporation experiment. Next, the cells were incubated with medium containing EdU working solution (10 μM, Beyotime) at 37 °C for 2 h. Then, the cells were fixed with 4% paraformaldehyde (Beyotime) and permeabilized with 0.3% Triton X-100 solution. Following permeabilization, the Click Additive Solution was applied, and the cell nuclei were subsequently stained with Hoechst 33342 (Beyotime). EdU-positive cells were quantified via fluorescence microscopy.

### Colony formation assay

CRC cells were seeded in a 12-well plate at a density of 1000 cells per well and cultured for 14 days. The culture supernatant was then removed, and the cells were fixed with 4% paraformaldehyde. The fixed cells were then stained with crystal violet solution (Beyotime), and images were acquired via an inverted microscope.

### Transwell migration and invasion assays

For the migration assay, 400 µL of serum-free culture medium containing 4 × 10^4^ CRC cells was added to the upper chamber of a Transwell apparatus with an 8 µm membrane (Corning). The lower chamber was filled with 400 µL of culture medium supplemented with 20% serum. For the invasion assay, the upper chamber membrane was coated with Matrigel (diluted 1:30; Corning). After incubation at 37 °C for 24–48 h, the cells that had migrated through or invaded the membrane were fixed with 4% paraformaldehyde and stained with crystal violet (Beyotime). Images were then acquired via an inverted microscope.

### Oil Red O staining

Oil Red O staining was performed via an Oil Red O kit according to the manufacturer’s protocol (Solarbio Life Sciences, Beijing, China). In brief, CRC cells were seeded on 14-mm circular coverslips in 24-well plates. After 24 h, the cells were washed twice with PBS and fixed with fixation buffer for 30 min. Then, the cells were washed twice with distilled water and incubated in 60% isopropyl alcohol for 5 min. The cells were incubated with Oil Red O solution for 20 min. After staining, the cells were washed with 60% isopropanol, washed with deionized water, and stained with haematoxylin for 2 min. Excess dye was discarded, and the cells were washed three times. Images were obtained by scanning oil red O-stained cell smears via an Aperio ImageScope (Leica Biosystems Imaging, California, USA). Lipid accumulation was quantified via the amount of oil-stained area via ImageJ, and the results were normalized to the average of the control [54].

### Enzyme-linked immunosorbent assay (ELISA)

The concentrations of human PC in the supernatants of HCT-116 and RKO cells were measured with ELISA kits (Tongwei, Shanghai, China) according to the manufacturer’s instructions. The cells were seeded into 6-well plates, and the supernatant was collected 24 h later and added to microplates precoated with PC-specific antibodies. Next, the samples, standard solutions, and HRP-labeled detection antibodies were added. The plates were then incubated and thoroughly washed. Color development was achieved via the use of a TMB substrate, which converts from blue to yellow by peroxidase under acidic conditions. The intensity of the yellow color is directly proportional to the PC concentration in the sample. The absorbance (OD value) was measured at a wavelength of 450 nm via an enzyme-labeled instrument, and the concentration of each sample was calculated.

The concentrations of human IFN-γ and granzyme B in the supernatant of NK92mi cells were determined by ELISA kit (#EHC102, #EHC117, Neobioscience, Shenzhen, China) according to the manufacturer’s instructions. Absorbance (OD value) was measured at a wavelength of 450 nm using an enzyme-linked immunosorbent assay reader, and the concentration of each analyte was calculated based on a standard curve.

### RNA sequencing and bioinformatic analysis

Total RNA was extracted from FASN knockdown RKO cells and control cells via Total RNA Extraction Reagent (Vazyme). RNA sequencing (RNA-Seq) was conducted by Shanghai Biotechnology (Shanghai, China). Differential gene expression was evaluated according to the criteria of foldchange >2 and *Q* value < 0.05. Volcano plots, Gene Ontology (GO) enrichment analysis, and sum count analysis were performed via the ggplot2, enrichGO, and ggrepel R packages. Differentially expressed genes (DEGs) were identified, and their correlations are detailed in Table S4.

### Promoter prediction analysis

The promoter sequence of the PLA2G4B gene was obtained from the GRCh38/hg38 human genome assembly via the University of California, Santa Cruz (UCSC) Genome Browser at the UCSC. JASPAR, TFDB, hTFTarget, ALGGEN PROMO, and GTRD databases were utilized to predict potential transcription factors and their binding sites. The intersections among these five datasets were visualized via Jvenn, a Venn diagram viewer, with overlaps depicted as circles. The full length of the promoter region sequence was listed in Supplementary Materials. Information on the predicted binding sites can be obtained from the official JASPAR website at https://jaspar.genereg.net/.

### Cleavage under targets and release using nuclease (Cut & Run)

The Cut&Run kit was purchased from Vazyme. Briefly, 1 million HCT-116 cells were captured with BioMag Plus Concanavalin A beads and incubated with anti-SP1 antibody (Proteintech) or control IgG (Beyotime) for 24 h at 4 °C. The cells were washed twice and incubated with pA/G-MNase at a 1:10,000 dilution in DIG wash buffer for 1 h at 4 °C. The cells were then washed twice and resuspended in calcium buffer to activate MNase. Following a 1.5 h incubation on ice, stop solution was added, and the cells were incubated for 30 min in a 37 °C incubator to release cleaved chromatin fragments. The supernatants were collected by centrifugation, and the DNA was extracted. Finally, quantitative detection was performed via PCR.

The search for candidate SP1-binding sites within the PLA2G4B promoter region was performed via the JASPAR database. The primers used for CUT&RUN are shown in Table S9.

### Chromatin immunoprecipitation (ChIP) assay

The ChIP assay was conducted via a BeyoChIP™ ChIP assay kit (Beyotime) following the manufacturer’s instructions. Briefly, to induce crosslinking of the chromatin, the cells were treated with 37% formaldehyde for 10 min. The chromatin was then sonicated to fragment DNA, followed by incubation with either anti-SP1 antibody (Cell Signalling Technology) or control IgG at 4 °C overnight. The immunoprecipitated DNA was then purified and used for PCR. The primer sequences are listed in Supplementary Table S9.

### Dual-luciferase reporter assay

The Dual-Luciferase Reporter Assay System (Yeasen, Shanghai, China) was used to assess the luciferase activity of the PLA2G4B promoter. FASN-deficient HCT-116 and RKO cells were transfected with the PLA2G4B luciferase reporter vector via Lipo8000™ Transfection Reagent and treated with plicamycin, an SP1 inhibitor (MedChemExpress, New Jersey, USA). After incubation for 48 h, the cells were lysed with PLB lysis solution, and the firefly and renilla luciferase activities were measured via a BioTek luminometer. The relative luciferase activity was calculated by dividing the firefly luciferase activity by the renilla luciferase activity, and it was normalized to the relative luciferase activity of sh-NC.

### Immune correlation and immune score in CRC patients

RNA-seq data and corresponding clinical information for CRC patients were obtained from the TCGA database (https://portal.gdc.cancer.gov/). The MCP-counter R package algorithm was used to analyze the expression distribution of various immune cells in FSN-low and FSN-high CRC tissues. Visualizations of the above results were generated via the ggplot2, ggpubr, and ggsci R packages.

### Cytotoxicity assay

CRC cells (Target, T) were pretreated with mitomycin C (10 μg/mL, Med Chem Express, USA) for 2 h. Subsequently, CRC cells and NK92-MI cells (Effector, E) were cocultured in 96-well plates at T/E ratios of 1:1, 1:5, 1:10, and 1:20 for 12 h. After incubation, the culture medium and suspended NK92-MI cells were removed from each well. Fresh medium (100 μL) and 10 μL of CCK-8 solution were then added to each well. After a 2 h incubation, the optical density (OD) at 450 nm was measured. The cytotoxicity of NK92-MI cells against CRC cells was calculated via the following formula: cytotoxicity (%) = 100 × [1−(OD of CRC cells cocultured with NK92-MI cells−OD of medium)/(OD of CRC cells−OD of medium)].

### In vivo mouse experiments

All animal experiments were conducted in accordance with the Institutional Guidelines of the Soochow Animal Care and Use Committee (No. 202407A0365). Six-week-old female BALB/c nude mice were obtained from the Shanghai Laboratory Animal Centre (Shanghai, China). The mice were randomly assigned to two groups, namely, sh-NC and sh-FASN (*n* = 6 per group). A mixture of HCT-116-shNC and HCT-116-shFASN cells in PBS was injected into the tail vein of each mouse at a dose of 2 × 10^6^ cells/100 μL per mouse. After 6 weeks, the mice were euthanized, and their lung tissues were collected for HE staining.

Six-week-old female C57BL/6J mice from the Shanghai Laboratory Animal Centre were divided into the following three groups: MC38-shNC (*n* = 6), MC38-shFASN (*n* = 6), and MC38-shFASN + PC (*n* = 6). Each mouse received a subcutaneous injection of 1 × 10^6^ MC38 cells in 100 μL of PBS. After 5 days, the mice in the MC38-shFASN + PC group (*n* = 6) were intraperitoneally injected with PC (18:0|18:1) (Merck Company, Darmstadt, Germany) at a dose of 2.5 mg/kg for 7 consecutive days. The other groups were injected with an equal volume of normal saline. The volume of the transplanted tumors was measured every 2 days via Vernier calipers. The tumor volume was calculated via the following formula: volume (mm^3^) = 0.5 × *L* (mm) × *S*^2^ (mm^2^); where *S* and *L* represent the minimum and maximum diameters of the tumor, respectively. On the 14th day of the experiment, the mice were euthanized, and the tumor tissues were excised and weighed.

### Multiplex immunohistochemistry (mIHC)

Multiplex immunohistochemistry (mIHC) was performed via a four-label five-color multiple fluorescence staining kit (AiFang Biological, Changsha, China). In brief, xenograft tissue sections were deparaffinized, rehydrated, and treated with citrate antigen repair buffer (pH 6.0). Following incubation with 1% hydrogen peroxide to inhibit endogenous peroxidases, the slides were blocked with 3% bovine serum albumin (BSA) for 30 min and stained with primary antibody overnight at 4 °C. After washing with PBS, the xenograft tissue sections were incubated with a secondary antibody at room temperature for 50 min, followed by incubation with tyramide signal amplification dye at room temperature for 10 min. Serial staining cycles were repeated until the identification of interesting targets. Finally, the slides were stained with DAPI and sealed with a coverslip. Multispectral images were obtained via a Vectra 3 automated quantitative pathology imaging system (Akoya Biosciences, Marlborough, USA). The following primary and secondary antibodies were used: anti-FASN antibody (Proteintech), anti-PLA2G4B antibody (Proteintech), anti-NK1.1 antibody (Cell Signaling Technology), and anti-rat/rabbit Polymer-HRP (Aifang Biotechnology).

### CRC clinical specimens and immunohistochemistry

The CRC tissue microarrays (TMAs; Aifang Biotechnology) contained 80 CRC tissue samples and 80 normal adjacent tissue (NAT) samples, along with corresponding follow-up data. Detailed clinicopathologic information for each patient is provided in Tables S5–S6. IHC experiments were performed according to previously described methods. The TMAs were deparaffinized, rehydrated, and stained with specific primary antibodies at 4 °C overnight. Then, the TMAs were incubated with a secondary antibody labeled with HRP at 37 °C for 1 h, followed by staining with 3,3′-diaminobenzidine (DAB). All the tissue sections were analyzed in a blinded manner by two experienced pathologists. The IHC score was calculated by multiplying the intensity of staining (negative, 0; mild, 1; moderate, 2; and severe, 3) by the area of staining (0, ≤25%; 1, >25% and ≤50%; 2, >50% and ≤75%; 3, >75%) [20].

### Statistical analysis

Statistical analysis was conducted via GraphPad Prism 8.0. Differences between the two groups were compared via Student’s *t*-test. One-way analysis of variance was used to compare three or more independent conditions or groups. The chi-square test was used to examine the correlation of FASN and PLA2G4B expression with the clinicopathological features of CRC patients. Survival analysis was performed via the Kaplan–Meier method with the log-rank test. All values are expressed as the mean ± standard deviation (SD), and the data represent the results of at least three independent experiments. Statistical significance was determined as a *P* value of <0.05.

## Supplementary information

Supplementary Material—Tables

Figure S1

Figure S2

Figure S3

Figure S4

Supplement Material—Figure legend

Original Data File

## Data Availability

The authors declare that all data generated or analyzed during this study are included in this published article. The data presented in this study are available on request from the corresponding authors.

## References

[1] Siegel RL, Miller KD, Wagle NS, Jemal A. Cancer statistics, 2023. CA Cancer J Clin. 2023;73:17–48. 36633525 10.3322/caac.21763

[2] Chen J, Zhu H, Yin Y, Jia S, Luo X. Colorectal cancer: metabolic interactions reshape the tumor microenvironment. Biochim Biophys Acta Rev Cancer. 2022;1877:188797. 36100193 10.1016/j.bbcan.2022.188797

[3] Bray F, Ferlay J, Soerjomataram I, Siegel RL, Torre LA, Jemal A. Global cancer statistics 2018: GLOBOCAN estimates of incidence and mortality worldwide for 36 cancers in 185 countries. CA Cancer J Clin. 2018;68:394–424. 30207593 10.3322/caac.21492

[4] Grothey A, Fakih M, Tabernero J. Management of BRAF-mutant metastatic colorectal cancer: a review of treatment options and evidence-based guidelines. Ann Oncol. 2021;32:959–67. 33836264 10.1016/j.annonc.2021.03.206

[5] Sedlak JC, Yilmaz ÖH, Roper J. Metabolism and colorectal cancer. Annu Rev Pathol Mech Dis. 2023;18:467–92. 10.1146/annurev-pathmechdis-031521-041113 PMC9877174 36323004

[6] Yang K, Wang X, Song C, He Z, Wang R, Xu Y, et al. The role of lipid metabolic reprogramming in tumor microenvironment. Theranostics. 2023;13:1774–808. 37064872 10.7150/thno.82920 PMC10091885

[7] Chen D, Zhou X, Yan P, Yang C, Li Y, Han L, et al. Lipid metabolism reprogramming in colorectal cancer. J Cell Biochem. 2023;124:3–16. 36334309 10.1002/jcb.30347

[8] Ecker J, Benedetti E, Kindt ASD, Höring M, Perl M, Machmüller AC, et al. The colorectal cancer lipidome: identification of a robust tumor-specific lipid species signature. Gastroenterology. 2021;161:910–23.e19. 34000281 10.1053/j.gastro.2021.05.009

[9] Hung C-Y, Yeh T-S, Tsai C-K, Wu R-C, Lai Y-C, Chiang M-H, et al. Glycerophospholipids pathways and chromosomal instability in gastric cancer: global lipidomics analysis. World J Gastrointest Oncol. 2019;11:181–94. 30918592 10.4251/wjgo.v11.i3.181 PMC6425327

[10] Tan SLW, Israeli E, Ericksen RE, Chow PKH, Han W. The altered lipidome of hepatocellular carcinoma. Semin Cancer Biol. 2022;86:445–56. 35131480 10.1016/j.semcancer.2022.02.004

[11] Cheng C, Geng F, Cheng X, Guo D. Lipid metabolism reprogramming and its potential targets in cancer. Cancer Commun. 2018;38:27. 10.1186/s40880-018-0301-4 PMC5993136 29784041

[12] Fhu CW, Ali A. Fatty acid synthase: an emerging target in cancer. Molecules. 2020;25:3935. 32872164 10.3390/molecules25173935 PMC7504791

[13] Crunkhorn S. Breast cancer: FASN inhibitor increases survival. Nat Rev Drug Discov. 2016;15:532. 27469233 10.1038/nrd.2016.148

[14] Li J, Huang Q, Long X, Zhang J, Huang X, Aa J, et al. CD147 reprograms fatty acid metabolism in hepatocellular carcinoma cells through Akt/mTOR/SREBP1c and P38/PPARα pathways. J Hepatol. 2015;63:1378–89. 26282231 10.1016/j.jhep.2015.07.039

[15] Wei W, Qin B, Wen W, Zhang B, Luo H, Wang Y, et al. FBXW7β loss-of-function enhances FASN-mediated lipogenesis and promotes colorectal cancer growth. Signal Transduct Target Ther. 2023;8:187. 37202390 10.1038/s41392-023-01405-8 PMC10195794

[16] Du Q, Liu P, Zhang C, Liu T, Wang W, Shang C, et al. FASN promotes lymph node metastasis in cervical cancer via cholesterol reprogramming and lymphangiogenesis. Cell Death Dis. 2022;13:488. 35597782 10.1038/s41419-022-04926-2 PMC9124199

[17] Gu L, Zhu Y, Lin X, Tan X, Lu B, Li Y. Stabilization of FASN by ACAT1-mediated GNPAT acetylation promotes lipid metabolism and hepatocarcinogenesis. Oncogene. 2020;39:2437–49. 31974474 10.1038/s41388-020-1156-0

[18] Yu W, Lei Q, Yang L, Qin G, Liu S, Wang D, et al. Contradictory roles of lipid metabolism in immune response within the tumor microenvironment. J Hematol Oncol. 2021;14:187. 34742349 10.1186/s13045-021-01200-4 PMC8572421

[19] Zipinotti Dos Santos D, de Souza JC, Pimenta TM, da Silva Martins B, Junior RSR, Butzene SMS, et al. The impact of lipid metabolism on breast cancer: a review about its role in tumorigenesis and immune escape. Cell Commun Signal. 2023;21:161. 37370164 10.1186/s12964-023-01178-1 PMC10304265

[20] Yi K, Zhan Q, Wang Q, Tan Y, Fang C, Wang Y, et al. PTRF/cavin-1 remodels phospholipid metabolism to promote tumor proliferation and suppress immune responses in glioblastoma by stabilizing cPLA2. Neuro Oncol. 2021;23:387–99. 33140095 10.1093/neuonc/noaa255 PMC7992898

[21] Cook KL, Soto-Pantoja DR, Clarke PAG, Cruz MI, Zwart A, Wärri A, et al. Endoplasmic reticulum stress protein GRP78 modulates lipid metabolism to control drug sensitivity and antitumor immunity in breast cancer. Cancer Res. 2016;76:5657–70. 27698188 10.1158/0008-5472.CAN-15-2616 PMC5117832

[22] Molendijk J, Kolka CM, Cairns H, Brosda S, Mohamed A, Shah AK, et al. Elevation of fatty acid desaturase 2 in esophageal adenocarcinoma increases polyunsaturated lipids and may exacerbate bile acid‐induced DNA damage. Clin Transl Med. 2022;12:e810. 35560527 10.1002/ctm2.810 PMC9099135

[23] Ross J, Najjar AM, Sankaranarayanapillai M, Tong WP, Kaluarachchi K, Ronen SM. Fatty acid synthase inhibition results in a magnetic resonance–detectable drop in phosphocholine. Mol Cancer Ther. 2008;7:2556–65. 18723500 10.1158/1535-7163.MCT-08-0015 PMC2553361

[24] Mao X, Lei H, Yi T, Su P, Tang S, Tong Y, et al. Lipid reprogramming induced by the TFEB-ERRα axis enhanced membrane fluidity to promote EC progression. J Exp Clin Cancer Res. 2022;41:28. 35045880 10.1186/s13046-021-02211-2 PMC8767755

[25] Bartolacci C, Andreani C, Vale G, Berto S, Melegari M, Crouch AC, et al. Targeting de novo lipogenesis and the Lands cycle induces ferroptosis in KRAS-mutant lung cancer. Nat Commun. 2022;13:4327. 35882862 10.1038/s41467-022-31963-4 PMC9325712

[26] Yu J, Wu C, Wu Q, Huang J, Fu W, Xie X, et al. PCYT1A suppresses proliferation and migration via inhibiting mTORC1 pathway in lung adenocarcinoma. Biochem Biophys Res Commun. 2020;529:353–61. 32703435 10.1016/j.bbrc.2020.05.164

[27] Uehara T, Kikuchi H, Miyazaki S, Iino I, Setoguchi T, Hiramatsu Y. et al. Overexpression of lysophosphatidylcholine acyltransferase 1 and concomitant lipid alterations in gastric cancer. Ann Surg Oncol. 2016;23(Suppl 2):S206–13. 25752890 10.1245/s10434-015-4459-6

[28] Liu Y, Yang C, Zhang Z, Jiang H. Gut microbiota dysbiosis accelerates prostate cancer progression through increased LPCAT1 expression and enhanced DNA repair pathways. Front Oncol. 2021;11:679712. 34221998 10.3389/fonc.2021.679712 PMC8249243

[29] Morita Y, Sakaguchi T, Ikegami K, Goto-Inoue N, Hayasaka T, Hang VT, et al. Lysophosphatidylcholine acyltransferase 1 altered phospholipid composition and regulated hepatoma progression. J Hepatol. 2013;59:292–9. 23567080 10.1016/j.jhep.2013.02.030

[30] Cho JH, Hong S-K, Kim E-Y, Park S-Y, Park C-H, Kim JM, et al. Overexpression of phospholipase D suppresses taxotere-induced cell death in stomach cancer cells. Biochim Biophys Acta. 2008;1783:912–23. 18190795 10.1016/j.bbamcr.2007.11.019

[31] Borel M, Cuvillier O, Magne D, Mebarek S, Brizuela L. Increased phospholipase D activity contributes to tumorigenesis in prostate cancer cell models. Mol Cell Biochem. 2020;473:263–79. 32661773 10.1007/s11010-020-03827-2

[32] Saito M, Iwadate M, Higashimoto M, Ono K, Takebayashi Y, Takenoshita S. Expression of phospholipase D2 in human colorectal carcinoma. Oncol Rep. 2007;18:1329–34. 17914593

[33] Lee J, Ridgway ND. Phosphatidylcholine synthesis regulates triglyceride storage and chylomicron secretion by Caco2 cells. J Lipid Res. 2018;59:1940–50. 30115754 10.1194/jlr.M087635 PMC6168309

[34] Saito RdF, Andrade LNdS, Bustos SO, Chammas R. Phosphatidylcholine-derived lipid mediators: the crosstalk between cancer cells and immune cells. Front Immunol. 2022;13:768606. 35250970 10.3389/fimmu.2022.768606 PMC8889569

[35] Ma S, Han J, Li Z, Xiao S, Zhang J, Yan J, et al. An XBP1s-PIM-2 positive feedback loop controls IL-15-mediated survival of natural killer cells. Sci Immunol. 2023;8:eabn7993. 36897958 10.1126/sciimmunol.abn7993 PMC10216801

[36] Song H, Song J, Cheng M, Zheng M, Wang T, Tian S, et al. METTL3-mediated m6A RNA methylation promotes the anti-tumour immunity of natural killer cells. Nat Commun. 2021;12:5522. 34535671 10.1038/s41467-021-25803-0 PMC8448775

[37] Koundouros N, Poulogiannis G. Reprogramming of fatty acid metabolism in cancer. Br J Cancer. 2020;122:4–22. 31819192 10.1038/s41416-019-0650-z PMC6964678

[38] Butler LM, Perone Y, Dehairs J, Lupien LE, de Laat V, Talebi A, et al. Lipids and cancer: emerging roles in pathogenesis, diagnosis and therapeutic intervention. Adv Drug Deliv Rev. 2020;159:245–93. 32711004 10.1016/j.addr.2020.07.013 PMC7736102

[39] Zhao Z, Xiao Y, Elson P, Tan H, Plummer SJ, Berk M, et al. Plasma lysophosphatidylcholine levels: potential biomarkers for colorectal cancer. J Clin Oncol. 2007;25:2696–701. 17602074 10.1200/JCO.2006.08.5571

[40] Kühn T, Floegel A, Sookthai D, Johnson T, Rolle-Kampczyk U, Otto W, et al. Higher plasma levels of lysophosphatidylcholine 18:0 are related to a lower risk of common cancers in a prospective metabolomics study. BMC Med. 2016;14:13. 26817443 10.1186/s12916-016-0552-3 PMC4730724

[41] Linkous A, Yazlovitskaya E. Cytosolic phospholipase A2 as a mediator of disease pathogenesis. Cell Microbiol. 2010;12:1369–77. 20642808 10.1111/j.1462-5822.2010.01505.x

[42] Raab S, Gadault A, Very N, Decourcelle A, Baldini S, Schulz C, et al. Dual regulation of fatty acid synthase (FASN) expression by O-GlcNAc transferase (OGT) and mTOR pathway in proliferating liver cancer cells. Cell Mol Life Sci. 2021;78:5397–413. 34046694 10.1007/s00018-021-03857-z PMC11072354

[43] Wu J, Liu F, Guo X, Cui C. FASN promotes anoikis resistance in colorectal liver metastases through the ERK1/2 pathway. Biochem Biophys Res Commun. 2024;736:150494. 39116680 10.1016/j.bbrc.2024.150494

[44] Gao Y, Lu J, Bao X, Yi X, Peng C, Chen W, et al. Inhibition of phospholipases suppresses progression of psoriasis through modulation of inflammation. Exp Biol Med. 2021;246:1253–62. 10.1177/1535370221993424 PMC8371307 33641447

[45] Recuero SdC, Viana NI, Reis ST, Mendes KT, Talib LL, Gattaz WF, et al. Phospholipase A2 expression in prostate cancer as a biomarker of good prognosis: a comprehensive study in patients with long follow-up. Urologia. 2024;91:720–6. 39051490 10.1177/03915603241257362

[46] Briggs MR, Kadonaga JT, Bell SP, Tjian R. Purification and biochemical characterization of the promoter-specific transcription factor, Sp1. Science. 1986;234:47–52. 3529394 10.1126/science.3529394

[47] Martin-Perez M, Urdiroz-Urricelqui U, Bigas C, Benitah SA. The role of lipids in cancer progression and metastasis. Cell Metab. 2022;34:1675–99. 36261043 10.1016/j.cmet.2022.09.023

[48] Björkström NK, Strunz B, Ljunggren H-G. Natural killer cells in antiviral immunity. Nat Rev Immunol. 2022;22:112–23. 34117484 10.1038/s41577-021-00558-3 PMC8194386

[49] Cózar B, Greppi M, Carpentier S, Narni-Mancinelli E, Chiossone L, Vivier E. Tumor-infiltrating natural killer cells. Cancer Discov. 2021;11:34–44. 33277307 10.1158/2159-8290.CD-20-0655 PMC7611243

[50] Sun Y, Hu H, Liu Z, Xu J, Gao Y, Zhan X, et al. Macrophage STING signaling promotes NK cell to suppress colorectal cancer liver metastasis via 4-1BBL/4-1BB co-stimulation. J Immunother Cancer. 2023;11:e006481. 36927529 10.1136/jitc-2022-006481 PMC10030919

[51] Chen Y, Chen K, Zhu H, Qin H, Liu J, Cao X. Methyltransferase Setd2 prevents T cell-mediated autoimmune diseases via phospholipid remodeling. Proc Natl Acad Sci USA. 2024;121:e2314561121. 38359295 10.1073/pnas.2314561121 PMC10895270

[52] Petkevicius K, Virtue S, Bidault G, Jenkins B, Çubuk C, Morgantini C, et al. Accelerated phosphatidylcholine turnover in macrophages promotes adipose tissue inflammation in obesity. eLife. 2019;8:e47990. 31418690 10.7554/eLife.47990 PMC6748830

[53] Tsugawa H, Arita M, Kanazawa M, Ogiwara A, Bamba T, Fukusaki E. MRMPROBS: a data assessment and metabolite identification tool for large-scale multiple reaction monitoring based widely targeted metabolomics. Anal Chem. 2013;85:5191–9. 23581547 10.1021/ac400515s

[54] Mehlem A, Hagberg CE, Muhl L, Eriksson U, Falkevall A. Imaging of neutral lipids by oil red O for analyzing the metabolic status in health and disease. Nat Protoc. 2013;8:1149–54. 23702831 10.1038/nprot.2013.055

