# Supplementary material for: FASN promotes lipid metabolism and progression in colorectal cancer via the SP1/PLA2G4B axis
Source: Cell Death Discov. 2025 Mar 28;11:122. doi: 10.1038/s41420-025-02409-9 (PMC11950308; doi:10.1038/s41420-025-02409-9)
Supplement: Supplementary file 1 — Supplementary Material—Tables [file 41420_2025_2409_MOESM1_ESM.pdf]

# FASN promotes lipid metabolism and progression in colorectal cancer via the SP1/PLA2G4B axis

## Tables

**Table S1. The clinical features of 14 CRC patients, used for Lipidomic analysis, were shown.**

| Characteristics | Count(n=14) | |
|---|---|---|
| | N | % |
| Sex | | |
| Female | 5 | 36% |
| male | 9 | 64% |
| Age | | |
| <60 | 4 | 29% |
| ≥60 | 10 | 71% |
| TNM Stage | | |
| I-II | 10 | 71% |
| III-IV | 4 | 29% |
| Tumor differentiatinon | | |
| Well and moderately differentiated | 9 | 64% |
| Poorly differentiated | 5 | 36% |
| Lymph node metastasis | | |
| + | 2 | 17% |
| - | 12 | 82% |

**Table S2. The information of 547 types of differentially expressed lipid identified**

| Lipid | P-value | log2(FC) | VIP | Up/Down |
|---|---|---|---|---|
| LPI(16:1) | 0.0004443 | 10.364559 | 0.0133798 | Up |
| PE(16:0p/18:3) | 0.0032302 | 6.0995061 | 0.0267445 | Up |
| PE(16:1/16:1) | 0.0021667 | 5.2910941 | 0.166879 | Up |
| PC(14:0/14:0) | 0.0001756 | 4.8151855 | 0.2096135 | Up |
| LPI(20:2) | 0.0002452 | 4.7293556 | 0.0181051 | Up |
| PE(14:0/16:1) | 0.0009986 | 4.577693 | 0.0412825 | Up |
| PC(14:0/16:1) | 0.0001294 | 4.2922909 | 0.2231894 | Up |
| PC(16:1/20:1) | 0.0001278 | 4.2212746 | 0.3867818 | Up |
| PE(16:1/20:1) | 8.418E-06 | 4.2101002 | 0.131312 | Up |
| PE(14:0/18:1) | 0.0005636 | 4.1978673 | 0.2143081 | Up |
| PE(16:0p/16:1) | 0.000122 | 3.9612415 | 0.2345777 | Up |
| PE(16:0/16:1) | 0.0001846 | 3.9576074 | 0.4285748 | Up |
| Hex2Cer(d18:1/16:0) | 0.0308199 | 3.9357538 | 0.2711414 | Up |
| PE(16:1/22:3) | 0.0003998 | 3.8186926 | 0.1557685 | Up |
| PE(16:1/22:1) | 9.205E-05 | 3.8077456 | 0.0549096 | Up |
| PC(16:1/16:1) | 8.277E-05 | 3.6365534 | 0.4011242 | Up |
| PA(18:2/20:5) | 0.0050745 | 3.6361428 | 0.0223483 | Up |
| PC(14:0/18:1) | 8.137E-05 | 3.6046602 | 0.4110826 | Up |
| PE(16:1/18:1) | 6.308E-05 | 3.5551856 | 0.4211621 | Up |
| PE(16:1/20:5) | 0.0152556 | 3.5206304 | 0.0256987 | Up |
| PE(16:1/22:2) | 0.0012429 | 3.4592981 | 0.0589326 | Up |
| PE(14:0/18:0) | 1.992E-05 | 3.4204498 | 0.0593639 | Up |
| LPC(14:1) | 0.0043769 | 3.3068929 | 0.0049335 | Up |
| PG(16:0/16:1) | 0.0042578 | 3.2662275 | 0.0316287 | Up |
| PC(16:1/20:3) | 0.0017917 | 3.2600062 | 0.102038 | Up |
| PC(16:1/20:2) | 6.643E-05 | 3.1997391 | 0.1390266 | Up |
| PC(16:1/22:6) | 0.0002325 | 3.1369958 | 0.0812644 | Up |
| Hex2Cer(d18:2/16:0) | 0.0410038 | 3.1171599 | 0.024393 | Up |
| Hex2Cer(d18:1/24:0) | 0.0273423 | 3.1024671 | 0.031517 | Up |
| Hex2Cer(d18:1/24:1) | 0.0165747 | 3.0978829 | 0.0468149 | Up |
| PE(14:0/16:0) | 0.0452057 | 3.0518694 | 0.0576489 | Up |
| PI(18:1/20:5) | 0.0003214 | 3.0305351 | 0.0620151 | Up |
| PE(16:1/18:2) | 0.0004191 | 3.0071444 | 0.1849295 | Up |
| PE(16:1/22:4) | 6.114E-05 | 2.9672662 | 0.0540396 | Up |
| MAG(16:1) | 0.0011596 | 2.93189 | 0.0156153 | Up |
| PC(14:0/16:0) | 5.943E-05 | 2.9281847 | 0.6482884 | Up |
| PC(14:0/18:0) | 0.0014321 | 2.9255133 | 0.2248203 | Up |
| PE(14:0/18:2) | 0.0005479 | 2.8771164 | 0.0621538 | Up |
| PC(14:0/18:3) | 7.634E-05 | 2.8701842 | 0.0432413 | Up |
| Hex2Cer(d18:1/18:0) | 0.0338856 | 2.834172 | 0.0289431 | Up |
| LPG(16:1) | 0.0038101 | 2.816046 | 0.0123368 | Up |

| | | | | |
|---|---|---|---|---|
| PI(18:0/20:1) | 0.0009425 | 2.8031905 | 0.085397 | Up |
| PG(18:1/22:6) | 0.0014547 | 2.7966077 | 0.0323555 | Up |
| Hex2Cer(d16:1/24:1) | 0.0328736 | 2.7827102 | 0.0077658 | Up |
| PC(16:1/18:1) | 3.461E-05 | 2.7584477 | 0.6974237 | Up |
| PC(14:0/18:2) | 0.0001808 | 2.6975209 | 0.2440688 | Up |
| Cholesterol sulfate | 0.0016053 | 2.6692609 | 0.4479803 | Up |
| LPE(16:1) | 0.0024619 | 2.663631 | 0.0645385 | Up |
| LPI(22:4) | 0.0016106 | 2.6625711 | 0.0145858 | Up |
| Hex2Cer(d18:1/22:0) | 0.0252765 | 2.6339429 | 0.0212319 | Up |
| PC(16:0/16:1) | 1.526E-05 | 2.6275012 | 0.7883382 | Up |
| PI(18:1/20:2) | 0.0024291 | 2.6166565 | 0.1521897 | Up |
| PC(18:2/22:3) | 0.0004926 | 2.6097953 | 0.1451702 | Up |
| PC(18:1/22:3) | 0.0002607 | 2.5478753 | 0.0930961 | Up |
| Hex2Cer(d18:2/24:1) | 0.0194849 | 2.5435 | 0.0118657 | Up |
| PE(16:1/20:4) | 0.0001035 | 2.5307045 | 0.0924297 | Up |
| LPC(14:0) | 2.003E-05 | 2.5226833 | 0.0642177 | Up |
| PC(16:1/22:4) | 0.000426 | 2.4828167 | 0.0604123 | Up |
| PE(16:0p/20:3) | 0.0021428 | 2.4520332 | 0.4124247 | Up |
| CE(20:5) | 0.0010311 | 2.4003614 | 0.3599149 | Up |
| PE(18:0p/16:1) | 3.579E-05 | 2.3978927 | 0.2115911 | Up |
| PA(18:2/22:5) | 0.0089636 | 2.3476967 | 0.0560364 | Up |
| LPE(17:1) | 0.0011597 | 2.3474872 | 0.0243442 | Up |
| CE(22:6) | 0.0164582 | 2.3336039 | 0.3586246 | Up |
| PC(18:2/22:2) | 7.389E-06 | 2.3146961 | 0.115375 | Up |
| LPS(18:2) | 0.0030534 | 2.3073077 | 0.0127301 | Up |
| LPE(24:2) | 0.0032921 | 2.292483 | 0.0209824 | Up |
| CE(22:4) | 0.0117253 | 2.2747543 | 0.40943 | Up |
| CE(24:1) | 0.0454795 | 2.2662894 | 0.2804271 | Up |
| LPS(22:4) | 0.0023515 | 2.2411129 | 0.0095398 | Up |
| LPC(O-24:1) | 0.0001993 | 2.2405553 | 0.1067941 | Up |
| LPI(18:2) | 0.0002506 | 2.2338223 | 0.0338346 | Up |
| PI(16:0/20:1) | 0.0006136 | 2.2087157 | 0.0644372 | Up |
| PA(18:1/22:6) | 0.0005712 | 2.1908543 | 0.124068 | Up |
| MAG(20:1) | 0.003382 | 2.178784 | 0.0274812 | Up |
| PA(16:0/20:5) | 0.0170337 | 2.174307 | 0.0240153 | Up |
| LPG(16:0) | 0.0014308 | 2.1741359 | 0.0143906 | Up |
| LPE(22:2) | 0.0058794 | 2.1661547 | 0.0189164 | Up |
| Hex3Cer(d18:1/16:0) | 5.221E-05 | 2.1569006 | 0.0595816 | Up |
| PC(16:1/20:5) | 0.0052142 | 2.1566247 | 0.0308524 | Up |
| LPE(24:1) | 0.0071827 | 2.1340843 | 0.0368589 | Up |
| PS(16:0/16:1) | 0.0102251 | 2.1237746 | 0.0665217 | Up |
| LPI(20:1) | 0.0031624 | 2.1134025 | 0.0083083 | Up |
| LPC(24:2) | 6.185E-05 | 2.0908607 | 0.0242723 | Up |
| CE(18:3) | 0.0073734 | 2.0760127 | 0.3758954 | Up |

| | | | | |
|---|---|---|---|---|
| PC(18:1/20:3) | 0.0014505 | 2.0727064 | 0.6215862 | Up |
| PA(18:2/22:6) | 0.0005316 | 2.0712264 | 0.0683007 | Up |
| PE(18:1/22:5) | 3.049E-05 | 2.0641669 | 0.2750732 | Up |
| Acar(14:0) | 0.0119858 | 2.0554615 | 0.0231243 | Up |
| PI(18:0/20:3) | 0.0005 | 2.0233151 | 0.5333171 | Up |
| LPC(24:1) | 0.0001596 | 2.0202928 | 0.034703 | Up |
| PA(16:0/16:1) | 0.0004266 | 2.016934 | 0.0209466 | Up |
| PC(16:1/18:3) | 0.0005404 | 2.0085674 | 0.0694877 | Up |
| PC(18:1/22:4) | 0.0024464 | 1.9977561 | 0.2649276 | Up |
| LPE(14:0) | 0.000362 | 1.9976738 | 0.02169 | Up |
| SM(d18:1/26:2) | 0.0007841 | 1.9941481 | 0.2231983 | Up |
| CE(22:5) | 0.0169396 | 1.9869998 | 0.3995098 | Up |
| PS(18:0/16:1) | 0.0058778 | 1.9848253 | 0.2309971 | Up |
| LPC(17:1) | 0.0004042 | 1.9581198 | 0.0869181 | Up |
| PE(18:1p/18:3) | 0.0028426 | 1.9571193 | 0.0543977 | Up |
| PE(16:0/22:0) | 3.503E-05 | 1.9544152 | 0.0237059 | Up |
| Cer(d18:2/14:0) | 7.676E-05 | 1.9474026 | 0.056482 | Up |
| LPI(18:1) | 0.0078552 | 1.9382131 | 0.0322412 | Up |
| LPE(20:5) | 0.0057812 | 1.9347374 | 0.0051028 | Up |
| PI(16:0/16:1) | 0.0077328 | 1.9298777 | 0.1551177 | Up |
| SM(d18:1/20:2) | 6.688E-05 | 1.9197542 | 0.108053 | Up |
| LPI(20:0) | 0.0004692 | 1.9186667 | 0.0075738 | Up |
| PE(18:1/20:2) | 4.568E-05 | 1.9177759 | 0.3283818 | Up |
| LPC(22:3) | 0.008329 | 1.9117374 | 0.0173907 | Up |
| PE(18:2/20:5) | 0.0018939 | 1.9008072 | 0.027607 | Up |
| LPC(22:2) | 0.0004045 | 1.8998375 | 0.0286915 | Up |
| MAG(18:1) | 0.0021698 | 1.8977392 | 0.1102492 | Up |
| LPI(20:4) | 0.0009236 | 1.8929809 | 0.0392016 | Up |
| PE(18:1/22:6) | 8.289E-05 | 1.8658552 | 0.3296324 | Up |
| LPG(22:6) | 0.0162095 | 1.8654605 | 0.0106657 | Up |
| PE(16:0/22:5) | 3.939E-05 | 1.860249 | 0.1776993 | Up |
| Acar(16:1) | 0.0217488 | 1.8596746 | 0.0176314 | Up |
| PC(16:1/18:2) | 6.74E-05 | 1.8540003 | 0.4083335 | Up |
| PE(18:1/22:1) | 0.0032001 | 1.8304098 | 0.2779653 | Up |
| LPC(O-24:2) | 0.0091697 | 1.8294852 | 0.0500914 | Up |
| PI(16:0/20:2) | 0.0024407 | 1.8285159 | 0.1669085 | Up |
| PC(16:0/16:2) | 0.000168 | 1.8192084 | 0.0943911 | Up |
| PE(18:1/20:1) | 0.0005559 | 1.8147227 | 0.3857924 | Up |
| LPE(22:3) | 0.0011047 | 1.8147162 | 0.0106442 | Up |
| CE(17:1) | 0.0141629 | 1.7749571 | 0.0606584 | Up |
| PE(18:1/18:3) | 0.0068858 | 1.7694841 | 0.073086 | Up |
| PI(18:0/18:1) | 0.0009319 | 1.7627663 | 0.6880603 | Up |
| LPS(20:3) | 0.0018296 | 1.7380518 | 0.0095231 | Up |
| PE(18:1p/20:3) | 0.0106767 | 1.7370696 | 0.2763008 | Up |

| | | | | |
|---|---|---|---|---|
| PG(18:1/18:3) | 0.0082786 | 1.7350701 | 0.0442743 | Up |
| PC(18:1/22:6) | 0.0004677 | 1.7289119 | 0.2291675 | Up |
| PE(16:0/20:3) | 0.0004682 | 1.7280714 | 0.2081088 | Up |
| PE(18:0/22:3) | 0.002478 | 1.7210188 | 0.0469948 | Up |
| PC(16:0/20:1) | 0.0001325 | 1.7202704 | 0.4643166 | Up |
| PC(16:1/20:4) | 1.686E-05 | 1.7170262 | 0.1387381 | Up |
| PE(18:1/20:5) | 0.0031339 | 1.7053116 | 0.0826124 | Up |
| DAG(16:0/22:0) | 0.0003799 | 1.7044763 | 0.058311 | Up |
| PE(18:0/20:5) | 0.0003177 | 1.7039141 | 0.0921908 | Up |
| Acar(20:2) | 0.0034227 | 1.7023413 | 0.0138901 | Up |
| PE(18:1/22:4) | 2.988E-05 | 1.7009044 | 0.1943698 | Up |
| PC(16:0/22:0) | 1.812E-06 | 1.693933 | 0.0523587 | Up |
| PG(18:1/20:4) | 0.0013214 | 1.6868881 | 0.0455822 | Up |
| PE(18:0/18:3) | 0.0010591 | 1.67451 | 0.0822211 | Up |
| PC(18:2/20:2) | 0.0009739 | 1.6725927 | 0.8139257 | Up |
| PE(18:1p/20:2) | 0.0003468 | 1.6708089 | 0.0770805 | Up |
| PC(16:0/22:4) | 0.002618 | 1.668194 | 0.4726491 | Up |
| SM(d18:1/26:0) | 0.0025932 | 1.661908 | 0.0833084 | Up |
| Cer(d18:1/26:0) | 1.345E-05 | 1.6599398 | 0.0891125 | Up |
| PE(18:0/20:1) | 0.000181 | 1.6583187 | 0.1112366 | Up |
| LPC(O-22:1) | 0.0008108 | 1.6520895 | 0.0447929 | Up |
| LPC(16:1) | 0.0005396 | 1.6508503 | 0.0983233 | Up |
| PE(18:1/18:1) | 0.0002003 | 1.642274 | 1.7601997 | Up |
| PE(18:0/22:5) | 0.0001404 | 1.6354315 | 0.1148768 | Up |
| PE(18:0/20:3) | 0.0009785 | 1.6241947 | 0.2396338 | Up |
| Acar(12:0) | 0.029096 | 1.6206416 | 0.0094384 | Up |
| PE(16:0p/18:2) | 0.0002926 | 1.6138635 | 0.5627448 | Up |
| PI(18:1/22:4) | 0.0016527 | 1.6051169 | 0.0799487 | Up |
| PE(16:0/20:5) | 0.0059513 | 1.6031672 | 0.0677064 | Up |
| Acar(20:1) | 0.0003924 | 1.6030459 | 0.0242907 | Up |
| PI(18:1/20:3) | 0.0044749 | 1.5998221 | 0.1611552 | Up |
| MAG(18:2) | 0.0063592 | 1.5925764 | 0.0353419 | Up |
| PC(18:1/20:5) | 0.0107379 | 1.5908901 | 0.1107172 | Up |
| PG(18:0/18:1) | 0.0002823 | 1.5895303 | 0.1104584 | Up |
| PI(18:0/22:5) | 0.0012654 | 1.5843704 | 0.1811175 | Up |
| LPG(22:5) | 0.0017782 | 1.5823963 | 0.0063638 | Up |
| LPE(20:2) | 0.0044894 | 1.5691623 | 0.0391034 | Up |
| LPC(O-20:1) | 0.0032342 | 1.5677318 | 0.0419269 | Up |
| Acar(24:1) | 0.0005716 | 1.565301 | 0.0055674 | Up |
| PE(18:0/20:2) | 4.267E-05 | 1.5622242 | 0.1248965 | Up |
| LPG(20:1) | 0.00104 | 1.5585043 | 0.0094853 | Up |
| HexCer(d18:1/16:0) | 0.0004947 | 1.556896 | 0.3791832 | Up |
| Hex2Cer(d18:1/20:0) | 0.0321928 | 1.5566303 | 0.0112556 | Up |
| LPS(18:1) | 0.0087596 | 1.5541354 | 0.0373074 | Up |

| | | | | |
|---|---|---|---|---|
| PC(18:1/20:2) | 0.0003248 | 1.5456112 | 0.4837812 | Up |
| PE(16:0p/20:2) | 0.0001947 | 1.537488 | 0.1250008 | Up |
| Acar(16:0) | 0.0051957 | 1.5365988 | 0.0604684 | Up |
| PE(16:0p/20:5) | 0.0005156 | 1.5343202 | 0.3665057 | Up |
| Acar(18:0) | 0.0034091 | 1.5338255 | 0.0546992 | Up |
| DAG(16:0/20:1) | 0.0285919 | 1.5319176 | 0.1766288 | Up |
| CE(22:0) | 0.0294843 | 1.5284164 | 0.0616604 | Up |
| LPE(20:1) | 0.0107386 | 1.5264546 | 0.0756815 | Up |
| LPC(20:1) | 0.0057505 | 1.5263501 | 0.1106453 | Up |
| PE(16:0/20:1) | 0.0007979 | 1.5262548 | 0.1001808 | Up |
| PE(18:2/18:3) | 0.0437155 | 1.5185909 | 0.045226 | Up |
| PC(18:0/22:1) | 0.0006586 | 1.5158548 | 0.0333482 | Up |
| Acar(18:1) | 0.0107492 | 1.5139247 | 0.0388539 | Up |
| PE(16:0p/16:0) | 0.00016 | 1.5121059 | 0.2661989 | Up |
| PG(18:0/20:4) | 0.0002566 | 1.4938723 | 0.0846928 | Up |
| LPC(24:4) | 0.0025521 | 1.4878788 | 0.0159439 | Up |
| CE(16:2) | 0.0315154 | 1.4865949 | 0.065593 | Up |
| HexCer(d18:2/24:0) | 8.175E-06 | 1.4862863 | 0.0139173 | Up |
| LPE(18:3) | 0.0209455 | 1.4835321 | 0.0091877 | Up |
| HexCer(d18:1/24:1) | 6.834E-05 | 1.4803082 | 0.0717043 | Up |
| PG(16:0/18:3) | 0.0041521 | 1.4671258 | 0.0044405 | Up |
| LPS(24:1) | 0.0301407 | 1.4659498 | 0.0090138 | Up |
| PG(18:2/20:5) | 0.0291665 | 1.4640361 | 0.0046018 | Up |
| PE(18:2/22:6) | 0.0003597 | 1.4591227 | 0.0731694 | Up |
| PE(18:1p/22:6) | 1.438E-05 | 1.4587212 | 0.2115463 | Up |
| PC(18:0/20:0) | 0.021057 | 1.4540551 | 0.0126425 | Up |
| LPC(24:0) | 0.000419 | 1.4459514 | 0.0342592 | Up |
| DAG(18:0/20:1) | 0.005226 | 1.4426215 | 0.0748034 | Up |
| LPC(22:4) | 0.0021113 | 1.4397185 | 0.0824708 | Up |
| PE(16:0/20:0) | 4.394E-05 | 1.4088578 | 0.0262869 | Up |
| LPC(22:1) | 0.0039244 | 1.3956517 | 0.0371768 | Up |
| PC(18:2/22:4) | 6.196E-05 | 1.3945918 | 0.0904809 | Up |
| LPI(18:0) | 0.0032014 | 1.3849931 | 0.0944831 | Up |
| LPE(22:4) | 0.0009901 | 1.3822452 | 0.0221634 | Up |
| PC(18:1/22:5) | 0.003126 | 1.381982 | 0.0862618 | Up |
| LPE(18:1) | 0.0082861 | 1.3814734 | 0.2287999 | Up |
| DAG(16:0/20:0) | 0.0058866 | 1.3708979 | 0.0752117 | Up |
| Acar(18:2) | 0.0160442 | 1.3698202 | 0.0174237 | Up |
| LPI(16:0) | 0.0060167 | 1.3693422 | 0.0300624 | Up |
| LPG(18:0) | 0.0028045 | 1.3682775 | 0.0137774 | Up |
| PE(16:0p/22:6) | 3.705E-05 | 1.3672449 | 0.2896556 | Up |
| Acar(22:0) | 0.0194101 | 1.3631972 | 0.0052951 | Up |
| LPG(18:1) | 0.0035536 | 1.3504403 | 0.0575177 | Up |
| PI(16:0/20:3) | 0.0139577 | 1.3502046 | 0.166773 | Up |

| | | | | |
|---|---|---|---|---|
| PE(18:0/18:2) | 2.957E-05 | 1.3495967 | 0.6938136 | Up |
| PG(16:0/20:4) | 0.0063174 | 1.3489327 | 0.0257557 | Up |
| TAG50:5(16:0) | 0.0013413 | 1.3476291 | 0.0135067 | Up |
| HexCer(d18:1/24:0) | 4.902E-05 | 1.3459851 | 0.0736808 | Up |
| FA(16:1) | 7.244E-05 | 1.3436652 | 0.2061986 | Up |
| LPE(22:1) | 0.0283319 | 1.3433531 | 0.0413572 | Up |
| FA(20:2) | 6.45E-05 | 1.3374983 | 0.2241928 | Up |
| LPE(24:0) | 0.0099707 | 1.3352416 | 0.0310613 | Up |
| LPG(18:3) | 0.0049802 | 1.3292427 | 0.0061364 | Up |
| LPE(22:6) | 0.0014834 | 1.3252984 | 0.0096234 | Up |
| MAG(20:2) | 0.0031037 | 1.3169286 | 0.0129565 | Up |
| LPC(15:0) | 0.0023711 | 1.3047104 | 0.0758737 | Up |
| LPE(20:3) | 0.0181368 | 1.3029775 | 0.0130901 | Up |
| PE(18:0p/20:3) | 0.0148779 | 1.3016121 | 0.248503 | Up |
| PE(16:0/20:2) | 0.0008612 | 1.2997864 | 0.1111013 | Up |
| PC(18:1/18:1) | 0.0006315 | 1.2955489 | 2.2974851 | Up |
| PA(16:0/18:0) | 0.0006571 | 1.2945195 | 0.1356861 | Up |
| PC(16:0/18:3) | 0.001897 | 1.2907973 | 0.2165627 | Up |
| PA(18:1/22:5) | 0.0046465 | 1.2893249 | 0.6844239 | Up |
| PE(18:2/20:4) | 0.001197 | 1.2859878 | 0.113238 | Up |
| LPC(20:5) | 0.0120831 | 1.2844721 | 0.0126035 | Up |
| PC(16:0/16:0) | 0.002126 | 1.284443 | 2.548765 | Up |
| DAG(16:0/20:2) | 0.0006 | 1.2765556 | 0.1314839 | Up |
| FA(20:1) | 0.001458 | 1.2751889 | 0.2046964 | Up |
| FA(24:1) | 0.0006482 | 1.2705694 | 0.1043394 | Up |
| PE(18:1/20:4) | 0.0001867 | 1.2693066 | 0.3449746 | Up |
| DAG(16:0/22:2) | 0.0228715 | 1.2674235 | 0.0432612 | Up |
| LPC(20:2) | 0.0020596 | 1.2650623 | 0.0720195 | Up |
| PC(16:0/20:2) | 0.0011244 | 1.2603262 | 0.4744387 | Up |
| PE(18:1p/20:1) | 0.0095495 | 1.260182 | 0.1083152 | Up |
| PE(18:0/22:4) | 0.003985 | 1.2578496 | 0.2744524 | Up |
| PC(18:2/22:5) | 0.0012228 | 1.2567638 | 0.0696681 | Up |
| PS(18:1/18:2) | 0.0325029 | 1.2367204 | 0.0842086 | Up |
| Acar(20:0) | 0.0019021 | 1.2343876 | 0.0162392 | Up |
| PE(16:0/18:1) | 0.0009254 | 1.2306073 | 0.7008411 | Up |
| PE(16:0p/18:1) | 2.871E-05 | 1.2231947 | 1.3577871 | Up |
| dhCer(d18:0/24:1) | 0.0090295 | 1.2224024 | 0.1267836 | Up |
| FA(22:4) | 0.0075357 | 1.2208733 | 0.3043509 | Up |
| PS(18:0/18:0) | 0.0004753 | 1.217673 | 0.0879639 | Up |
| PE(18:1p/18:1) | 0.0001134 | 1.217494 | 0.472772 | Up |
| PG(18:2/20:4) | 0.0097735 | 1.2113119 | 0.0273562 | Up |
| PE(18:1p/22:5) | 0.0001284 | 1.2068698 | 0.086987 | Up |
| PE(18:1p/18:2) | 0.0029152 | 1.2067515 | 0.3275752 | Up |
| PE(18:2p/22:6) | 0.0005681 | 1.2061895 | 0.0948616 | Up |

| | | | | |
|---|---|---|---|---|
| PE(18:0/18:0) | 0.0002053 | 1.2054992 | 0.0684982 | Up |
| PE(18:1/18:2) | 0.0010919 | 1.1966855 | 0.5694573 | Up |
| PC(18:2/20:3) | 0.0039781 | 1.1928952 | 0.1854381 | Up |
| HexCer(d18:2/18:0) | 5.524E-05 | 1.192757 | 0.0198595 | Up |
| FA(22:3) | 0.0012413 | 1.1899546 | 0.0510573 | Up |
| PE(18:0/18:1) | 9.246E-05 | 1.1852764 | 0.7568423 | Up |
| Sphinganine | 0.0009396 | 1.1797448 | 0.0089916 | Up |
| DAG(18:1/20:3) | 0.0087051 | 1.1782911 | 0.1080403 | Up |
| PC(18:2/22:6) | 0.0037384 | 1.1774066 | 0.1336459 | Up |
| LPC(20:3) | 0.007769 | 1.1758718 | 0.0874311 | Up |
| Acar(16:3) | 0.0294994 | 1.1709429 | 0.0014375 | Up |
| PE(18:1p/20:4) | 0.000124 | 1.1700063 | 0.4790978 | Up |
| PG(16:0/18:0) | 0.0073486 | 1.1695414 | 0.0281417 | Up |
| PG(18:1/18:2) | 0.002219 | 1.1605027 | 0.1015906 | Up |
| FA(24:0) | 9.564E-05 | 1.1562034 | 0.0597653 | Up |
| PC(16:0/20:3) | 0.0255958 | 1.1554095 | 0.3218607 | Up |
| PE(18:2/18:2) | 0.0291489 | 1.1531877 | 0.2259893 | Up |
| PI(18:0/22:6) | 0.0028469 | 1.1516731 | 0.1636656 | Up |
| PI(16:0/22:4) | 0.0010011 | 1.1512509 | 0.0867902 | Up |
| dhCer(d18:1/24:0) | 0.000225 | 1.1481404 | 0.1414269 | Up |
| LPS(24:0) | 0.0472246 | 1.1402349 | 0.0080587 | Up |
| Cer(d18:2/16:0) | 0.0020577 | 1.1361591 | 0.186785 | Up |
| LPE(20:4) | 0.0006429 | 1.1352013 | 0.0390403 | Up |
| PE(16:0p/20:1) | 0.0029482 | 1.1317643 | 0.171148 | Up |
| PE(16:0p/20:4) | 5.804E-05 | 1.1304367 | 0.5919054 | Up |
| PE(16:0p/22:5) | 0.0003858 | 1.128285 | 0.1283603 | Up |
| LPC(18:1) | 0.0062837 | 1.1220101 | 0.7845058 | Up |
| PC(16:0/18:0) | 3.253E-05 | 1.1206265 | 0.6050095 | Up |
| PC(18:1/18:3) | 0.0171009 | 1.1134676 | 0.1608818 | Up |
| PE(18:1p/20:5) | 0.0077078 | 1.1124407 | 0.1631717 | Up |
| PE(18:1p/22:4) | 0.0006572 | 1.1091618 | 0.2952346 | Up |
| PG(18:0/20:3) | 0.0165844 | 1.0976595 | 0.0102506 | Up |
| PE(18:1p/16:0) | 0.0052148 | 1.0950472 | 0.370177 | Up |
| FA(20:3) | 0.0176413 | 1.0906958 | 0.119014 | Up |
| Cer(d18:2/17:0) | 0.0075768 | 1.0906348 | 0.0236274 | Up |
| DAG(18:0/20:0) | 0.0031999 | 1.0892189 | 0.0360216 | Up |
| PE(16:0/18:2) | 0.0009991 | 1.0883632 | 0.2950668 | Up |
| Cer(d18:2/26:0) | 0.0035749 | 1.0868164 | 0.0139948 | Up |
| Hex3Cer(d18:1/24:1) | 0.0040498 | 1.0842321 | 0.0189552 | Up |
| PA(18:2/22:4) | 0.0060847 | 1.0841063 | 0.0268393 | Up |
| DAG(18:2/20:4) | 0.0094909 | 1.0813702 | 0.0701578 | Up |
| PG(18:0/20:2) | 0.0187913 | 1.0811659 | 0.0168273 | Up |
| PG(16:0/18:2) | 0.0075505 | 1.0791499 | 0.0468039 | Up |
| Acar(22:1) | 0.0083965 | 1.0780434 | 0.0091904 | Up |

| | | | | |
|---|---|---|---|---|
| LPG(20:3) | 0.015308 | 1.068496 | 0.010575 | Up |
| DAG(18:2/20:2) | 0.0086787 | 1.0661146 | 0.0674233 | Up |
| LPE(18:2) | 0.0128945 | 1.0637015 | 0.0513498 | Up |
| PI(18:1/20:4) | 0.0109971 | 1.0627532 | 0.2599514 | Up |
| PA(18:0/22:6) | 0.0005322 | 1.0561203 | 0.1263575 | Up |
| HexCer(d16:1/24:0) | 0.0118745 | 1.0440381 | 0.0051212 | Up |
| PI(18:1/18:2) | 0.0442988 | 1.0390745 | 0.2667896 | Up |
| PE(16:0/18:0) | 4.607E-06 | 1.0376946 | 0.1447058 | Up |
| LPC(O-18:1) | 0.0009726 | 1.0323338 | 0.1013116 | Up |
| Cer(d18:1/24:0) | 0.0003275 | 1.0316654 | 0.3389899 | Up |
| dhCer(d18:1/24:1) | 0.0013573 | 1.0316613 | 0.1648832 | Up |
| LPS(16:0) | 0.0425803 | 1.024074 | 0.0062803 | Up |
| PI(18:0/20:4) | 0.0040339 | 1.0207895 | 1.1426637 | Up |
| LPG(22:4) | 0.0193498 | 1.0157394 | 0.0119139 | Up |
| SM(d18:1/14:0) | 0.0002192 | 1.0150371 | 0.2095681 | Up |
| PE(18:0/22:6) | 0.0002265 | 1.0143549 | 0.3372723 | Up |
| LPS(20:4) | 0.0011658 | 1.0142079 | 0.0080151 | Up |
| PI(18:1/22:6) | 0.0040867 | 1.0124004 | 0.1315395 | Up |
| LPE(19:0) | 0.0195215 | 1.0085376 | 0.0167021 | Up |
| PC(18:0/18:1) | 0.0002852 | 1.0059011 | 0.5981057 | Up |
| PE(16:0p/22:4) | 0.0016463 | 1.0041969 | 0.5077223 | Up |
| PE(18:0p/20:2) | 0.0021589 | 1.0030602 | 0.0927909 | Up |
| LPC(18:3) | 0.0102792 | 1.0027772 | 0.0201716 | Up |
| PE(16:0/20:4) | 0.0006594 | 0.9999545 | 0.3263212 | Up |
| PE(18:0/22:1) | 0.0096464 | 0.9947719 | 0.0216219 | Up |
| PE(16:0/22:4) | 0.0002643 | 0.9936267 | 0.1202175 | Up |
| LPC(22:5) | 0.0266327 | 0.9902426 | 0.0209448 | Up |
| LPG(18:2) | 0.0209895 | 0.982944 | 0.0330665 | Up |
| Cer(d19:1/26:0) | 0.0023134 | 0.9817551 | 0.0067503 | Up |
| LPE(18:0) | 0.0059563 | 0.9776179 | 0.2706187 | Up |
| Cer(d20:1/26:0) | 0.0033527 | 0.9733035 | 0.0079722 | Up |
| PA(18:0/18:0) | 0.0007191 | 0.9626044 | 0.0661546 | Up |
| PE(16:0/16:0) | 0.0022626 | 0.9610548 | 0.1649364 | Up |
| PS(18:1/22:4) | 0.0235869 | 0.9542649 | 0.1674833 | Up |
| PC(18:2/20:5) | 0.0311564 | 0.9483474 | 0.0399364 | Up |
| PE(16:0p/18:0) | 0.0001978 | 0.9403512 | 0.0541827 | Up |
| PA(16:0/20:1) | 0.0383329 | 0.9362779 | 0.2110153 | Up |
| FA(17:1) | 0.0064224 | 0.9239896 | 0.0405485 | Up |
| SM(d18:1/26:1) | 0.0009854 | 0.9207893 | 0.154063 | Up |
| PI(16:0/22:5) | 0.0155062 | 0.920741 | 0.0626277 | Up |
| HexCer(d18:2/22:0) | 0.0007463 | 0.9202199 | 0.0080617 | Up |
| Cer(d18:1/24:1) | 0.0018312 | 0.9191951 | 0.4063105 | Up |
| DAG(18:2/20:3) | 0.0437083 | 0.9191211 | 0.0727388 | Up |
| Cer(d20:1/24:1) | 0.0372055 | 0.913254 | 0.036466 | Up |

| | | | | |
|---|---|---|---|---|
| PA(18:0/18:1) | 0.0013602 | 0.9085311 | 0.0439781 | Up |
| PC(16:0/22:6) | 0.0017589 | 0.9050374 | 0.2163011 | Up |
| PC(18:0/20:5) | 0.018223 | 0.9014426 | 0.0554481 | Up |
| DAG(18:0/20:2) | 0.0014065 | 0.9009807 | 0.0772618 | Up |
| FA(22:5) | 0.005035 | 0.8984494 | 0.0987443 | Up |
| PI(18:0/18:2) | 0.0239134 | 0.8979689 | 0.7238762 | Up |
| DAG(16:0/20:3) | 0.0136216 | 0.8978466 | 0.1348376 | Up |
| dhCer(d18:0/16:0) | 0.045344 | 0.8971373 | 0.2033752 | Up |
| DAG(16:0/18:0) | 0.0050101 | 0.8943667 | 0.2633851 | Up |
| PC(16:0/20:5) | 0.0104273 | 0.8920177 | 0.1049307 | Up |
| PI(16:0/22:6) | 0.0202849 | 0.885959 | 0.0900434 | Up |
| SM(d18:1/18:2) | 0.0316427 | 0.8776 | 0.0269712 | Up |
| FA(22:6) | 0.0114247 | 0.865032 | 0.2292697 | Up |
| LPC(O-18:0) | 0.0109579 | 0.8545191 | 0.0836235 | Up |
| LPE(15:0) | 0.0400054 | 0.8501184 | 0.0103296 | Up |
| PE(18:0/22:0) | 0.046074 | 0.8498421 | 0.0070708 | Up |
| PG(18:0/20:1) | 0.0185771 | 0.84863 | 0.015003 | Up |
| PE(18:0p/18:2) | 0.0219267 | 0.8371042 | 0.4892193 | Up |
| PA(16:0/20:0) | 0.0050289 | 0.8326748 | 0.2472896 | Up |
| HexCer(d18:1/22:0) | 0.0015057 | 0.8276176 | 0.0372651 | Up |
| Hex3Cer(d18:1/24:0) | 0.0219439 | 0.8270852 | 0.0080893 | Up |
| PC(16:0/22:5) | 0.0036065 | 0.8228071 | 0.0984858 | Up |
| PE(16:0/22:1) | 0.0301556 | 0.8200005 | 0.0187683 | Up |
| PI(16:0/20:4) | 0.0141054 | 0.8171067 | 0.231356 | Up |
| dhCer(d18:0/22:0) | 0.0393173 | 0.8168472 | 0.0538667 | Up |
| SM(d18:1/14:1) | 0.0044373 | 0.8118385 | 0.0312226 | Up |
| PC(18:1/20:4) | 0.0059829 | 0.8113704 | 0.2580323 | Up |
| LPC(19:0) | 0.0140034 | 0.8104563 | 0.0184381 | Up |
| PC(18:0/22:6) | 0.0237241 | 0.809597 | 0.2180369 | Up |
| PA(16:0/18:1) | 0.0066421 | 0.8085593 | 0.0576812 | Up |
| LPE(17:0) | 0.0352724 | 0.8037469 | 0.0359725 | Up |
| PE(18:0/20:4) | 0.0009165 | 0.8024415 | 0.505108 | Up |
| dhCer(d18:0/24:0) | 0.0381388 | 0.794589 | 0.1210637 | Up |
| Cer(d16:1/24:1) | 0.0386239 | 0.7938574 | 0.0499975 | Up |
| LPE(16:0) | 0.0472964 | 0.7871512 | 0.1316781 | Up |
| PC(18:2/20:4) | 0.008012 | 0.7865059 | 0.2091416 | Up |
| PC(18:1/18:2) | 0.0035512 | 0.7812114 | 0.8179328 | Up |
| Cer(d19:1/24:0) | 0.0220823 | 0.7721917 | 0.0281795 | Up |
| DAG(16:0/20:5) | 0.0460464 | 0.770046 | 0.0168314 | Up |
| LPG(20:4) | 0.0343559 | 0.7691332 | 0.009893 | Up |
| SM(d18:1/22:1) | 0.0336447 | 0.762501 | 0.172857 | Up |
| PE(18:0p/16:0) | 0.0094567 | 0.7613471 | 0.1829501 | Up |
| SM(d18:1/16:1) | 0.001313 | 0.7570402 | 0.2073494 | Up |
| PE(18:0p/18:1) | 0.0048436 | 0.7537647 | 0.7419784 | Up |

| | | | | |
|---|---|---|---|---|
| LPC(18:0) | 0.0169386 | 0.7359292 | 0.233323 | Up |
| LPC(O-22:0) | 0.0177938 | 0.7275816 | 0.0341387 | Up |
| PE(18:2p/20:4) | 0.0262835 | 0.7242082 | 0.143283 | Up |
| Cer(d18:1/23:0) | 0.0154471 | 0.7132536 | 0.1181628 | Up |
| Cer(d19:1/24:1) | 0.0272388 | 0.7100664 | 0.0304491 | Up |
| PS(18:1/20:4) | 0.033105 | 0.708218 | 0.0517043 | Up |
| FA(18:1) | 0.0010616 | 0.7065138 | 0.7220195 | Up |
| LPC(16:0) | 0.0499472 | 0.688225 | 0.6661865 | Up |
| LPC(22:0) | 0.0260323 | 0.6855043 | 0.0095433 | Up |
| PE(16:0/22:2) | 0.0166754 | 0.684157 | 0.0893203 | Up |
| PE(18:0/20:0) | 0.0210539 | 0.6735257 | 0.0083955 | Up |
| MAG(20:0) | 0.010077 | 0.6700171 | 0.0159561 | Up |
| dhCer(d18:1/22:0) | 0.0459708 | 0.6484157 | 0.0429131 | Up |
| LPC(20:4) | 0.048947 | 0.6415682 | 0.0696999 | Up |
| Cer(d18:1/22:0) | 0.0448591 | 0.6319052 | 0.1162227 | Up |
| DAG(18:0/18:0) | 0.014729 | 0.6257507 | 0.0956582 | Up |
| PC(18:0/22:5) | 0.0124731 | 0.6237322 | 0.0733025 | Up |
| PC(16:0/18:2) | 0.010242 | 0.5950465 | 0.8002865 | Up |
| Cer(d17:1/24:1) | 0.0398994 | 0.5933385 | 0.0450734 | Up |
| PE(18:0p/20:1) | 0.0370776 | 0.5897032 | 0.0839915 | Up |
| PE(18:0p/22:6) | 0.0241669 | 0.5626401 | 0.4410054 | Up |
| PE(18:1/22:3) | 0.0354462 | 0.5596273 | 0.091613 | Up |
| PE(18:1/22:2) | 0.0181452 | 0.5465968 | 0.2260181 | Up |
| SM(d18:1/22:2) | 0.012096 | 0.5009225 | 0.5135187 | Up |
| DAG(18:0/20:3) | 0.0487648 | 0.4695546 | 0.0867807 | Up |
| FA(18:2) | 0.0373144 | 0.4439758 | 0.4299711 | Up |
| SM(d18:1/24:0) | 0.0297084 | 0.4414728 | 0.3401384 | Up |
| MAG(18:0) | 0.0407242 | 0.3925382 | 0.1012218 | Up |
| PE(16:1/20:3) | 0.0140907 | 0.3743967 | 0.0389917 | Up |
| Sph(d16:1) | 0.0460927 | -0.694844 | 0.0055448 | Down |
| SM(d18:1/20:0) | 0.0171975 | -0.797637 | 0.1893513 | Down |
| SM(d18:1/16:0) | 0.0005522 | -1.081779 | 0.9641053 | Down |
| TAG49:0(18:0) | 0.0316735 | -1.269749 | 0.1294351 | Down |
| PE(18:0p/20:0) | 0.0058613 | -1.330397 | 0.0097932 | Down |
| SM(d18:1/20:1) | 0.0066585 | -1.346695 | 0.1112435 | Down |
| TAG53:4(20:4) | 0.019325 | -1.449423 | 0.1191318 | Down |
| TAG54:0(16:0) | 0.0428405 | -1.517079 | 0.0871293 | Down |
| TAG58:7(20:4) | 0.0403357 | -1.71218 | 0.3338925 | Down |
| TAG56:4(20:4) | 0.0047638 | -1.894439 | 0.3303268 | Down |
| TAG47:0(16:0) | 0.0315344 | -1.988443 | 0.3922254 | Down |
| TAG58:7(18:0) | 0.0138208 | -2.093006 | 0.1769962 | Down |
| TAG55:5(20:4) | 0.0084429 | -2.131712 | 0.2072214 | Down |
| TAG53:1(16:0) | 0.0147269 | -2.145609 | 0.3061976 | Down |
| TAG51:0(16:0) | 0.0038167 | -2.196827 | 0.3987795 | Down |

| | | | | |
|---|---|---|---|---|
| TAG49:0(16:0) | 0.0031337 | -2.29436 | 0.4254585 | Down |
| TAG56:5(20:4) | 0.0044665 | -2.329186 | 0.9534639 | Down |
| TAG58:7(22:6) | 0.0189465 | -2.589002 | 0.683506 | Down |
| TAG55:7(22:6) | 0.0091614 | -2.699224 | 0.2555589 | Down |
| TAG56:5(18:0) | 0.0045125 | -2.700356 | 0.8681481 | Down |
| TAG58:7(16:0) | 0.0049533 | -2.73416 | 0.3137486 | Down |
| TAG50:0(18:0) | 0.0005752 | -2.804193 | 1.4504545 | Down |
| TAG58:9(20:4) | 0.0130657 | -2.852996 | 0.7999161 | Down |
| TAG56:6(18:0) | 0.0028705 | -2.881022 | 0.5442706 | Down |
| TAG58:6(18:0) | 0.0118773 | -2.920766 | 0.6628162 | Down |
| TAG58:10(20:4) | 0.0175355 | -3.086072 | 0.7826264 | Down |
| TAG60:10(22:6) | 0.0068341 | -3.174441 | 0.5748294 | Down |
| TAG49:1(16:0) | 0.0193636 | -3.206032 | 1.0233679 | Down |
| TAG56:4(18:0) | 0.0179237 | -3.227635 | 1.3310581 | Down |
| TAG49:2(16:0) | 0.0358933 | -3.242114 | 0.441946 | Down |
| TAG58:6(16:0) | 0.0025635 | -3.253375 | 0.3957715 | Down |
| TAG52:4(20:4) | 0.0024954 | -3.277836 | 1.0409865 | Down |
| TAG51:1(16:0) | 0.008355 | -3.32232 | 0.8933623 | Down |
| TAG52:3(18:0) | 0.0366522 | -3.382839 | 0.710814 | Down |
| TAG46:0(18:0) | 0.0366998 | -3.484037 | 0.1833561 | Down |
| TAG54:4(22:4) | 0.00143 | -3.522665 | 0.7296693 | Down |
| TAG54:6(22:6) | 0.0039182 | -3.596063 | 1.0242385 | Down |
| TAG58:8(22:6) | 0.0288619 | -3.630836 | 1.6454804 | Down |
| TAG56:6(20:4) | 0.0230271 | -3.690315 | 2.1489475 | Down |
| TAG46:0(16:0) | 0.0103296 | -3.787118 | 1.0503717 | Down |
| TAG54:4(16:0) | 0.001589 | -3.808448 | 2.1847787 | Down |
| TAG54:4(18:0) | 0.0044907 | -3.845293 | 2.538743 | Down |
| TAG51:2(16:0) | 0.0138762 | -3.848419 | 1.1307492 | Down |
| TAG58:6(22:4) | 0.0062622 | -3.893633 | 1.5957824 | Down |
| TAG56:4(16:0) | 0.0084326 | -3.909889 | 1.0057463 | Down |
| TAG57:10(22:6) | 0.0270308 | -3.935033 | 0.0076808 | Down |
| TAG52:2(18:0) | 0.0051299 | -3.967745 | 3.2449525 | Down |
| TAG54:5(20:4) | 0.0065848 | -3.967833 | 2.8189537 | Down |
| TAG60:11(22:6) | 0.0083304 | -4.012014 | 0.7768116 | Down |
| TAG56:9(20:5) | 0.0276266 | -4.046434 | 0.173447 | Down |
| TAG54:5(18:0) | 0.0261416 | -4.04776 | 0.8059245 | Down |
| TAG53:1(18:0) | 0.0029843 | -4.088023 | 1.052 | Down |
| TAG52:0(18:0) | 0.0076576 | -4.103871 | 2.393498 | Down |
| TAG58:7(22:4) | 0.0064814 | -4.117101 | 1.5332055 | Down |
| TAG56:7(22:6) | 0.0062217 | -4.142826 | 2.6131835 | Down |
| TAG54:0(18:0) | 0.0039833 | -4.195323 | 1.6908922 | Down |
| TAG56:5(22:4) | 0.0006295 | -4.277181 | 2.0889687 | Down |
| TAG54:3(18:0) | 0.0060233 | -4.298172 | 4.967326 | Down |
| TAG54:3(16:0) | 0.0034063 | -4.318613 | 2.7274294 | Down |

| | | | | |
|---|---|---|---|---|
| TAG54:5(16:0) | 0.0055678 | -4.330681 | 2.4238858 | Down |
| TAG56:3(18:0) | 0.0080436 | -4.34663 | 2.0383126 | Down |
| TAG52:3(16:0) | 0.008895 | -4.395897 | 7.7731207 | Down |
| TAG56:5(16:0) | 0.0009545 | -4.410027 | 1.8750717 | Down |
| TAG53:4(16:0) | 0.001529 | -4.432995 | 0.3705553 | Down |
| TAG52:5(20:5) | 0.0081691 | -4.475743 | 0.3623933 | Down |
| TAG50:1(16:0) | 0.016079 | -4.583875 | 6.756147 | Down |
| TAG50:2(16:0) | 0.0135328 | -4.59587 | 6.6004681 | Down |
| TAG52:1(18:0) | 0.0067292 | -4.608549 | 5.4915719 | Down |
| TAG54:8(20:5) | 0.0286751 | -4.63338 | 0.1495504 | Down |
| TAG44:0(16:0) | 0.011446 | -4.659299 | 0.6936226 | Down |
| TAG52:2(16:0) | 0.021101 | -4.662885 | 8.425913 | Down |
| TAG48:1(16:0) | 0.011223 | -4.671264 | 3.3138206 | Down |
| TAG56:8(20:5) | 0.0423842 | -4.672839 | 0.4341234 | Down |
| TAG52:4(16:0) | 0.0094032 | -4.676424 | 6.6309095 | Down |
| TAG52:0(16:0) | 0.0049356 | -4.695765 | 2.071811 | Down |
| TAG56:6(16:0) | 0.0014143 | -4.704211 | 2.2608801 | Down |
| TAG54:2(18:0) | 0.0119988 | -4.725839 | 6.7066166 | Down |
| TAG54:1(18:0) | 0.0075478 | -4.730332 | 5.0943433 | Down |
| TAG56:6(22:4) | 0.0013132 | -4.774388 | 2.1138534 | Down |
| TAG54:5(20:5) | 0.0171286 | -4.792495 | 0.2922142 | Down |
| TAG42:0(16:0) | 0.0037969 | -4.81156 | 0.2762499 | Down |
| TAG52:1(16:0) | 0.0085952 | -4.817101 | 5.7319825 | Down |
| TAG53:2(16:0) | 0.0146026 | -4.858626 | 1.3339786 | Down |
| TAG40:0(16:0) | 0.0060196 | -4.933909 | 0.1260205 | Down |
| TAG54:5(22:4) | 0.0037361 | -4.941478 | 1.0589209 | Down |
| TAG52:4(22:4) | 0.0008142 | -4.999992 | 0.5622879 | Down |
| TAG54:6(20:4) | 0.0057999 | -5.006873 | 3.5121398 | Down |
| TAG58:9(22:6) | 0.0178421 | -5.070732 | 2.4014358 | Down |
| TAG54:1(16:0) | 0.0223629 | -5.077367 | 2.6674831 | Down |
| TAG54:6(20:5) | 0.0244415 | -5.09689 | 0.9106137 | Down |
| TAG56:7(16:0) | 0.0011893 | -5.108945 | 2.0190456 | Down |
| TAG54:6(16:0) | 0.0040837 | -5.12225 | 2.0298075 | Down |
| TAG56:2(18:0) | 0.0311544 | -5.178288 | 2.9979477 | Down |
| TAG53:0(16:0) | 0.0151915 | -5.215255 | 0.4018012 | Down |
| TAG50:6(20:4) | 0.003577 | -5.242246 | 0.2564503 | Down |
| TAG54:2(16:0) | 0.0203031 | -5.243482 | 4.0173705 | Down |
| TAG42:1(16:0) | 0.0164339 | -5.247351 | 0.2061049 | Down |
| TAG44:1(16:0) | 0.0063268 | -5.29496 | 0.5383962 | Down |
| TAG50:3(16:0) | 0.0170341 | -5.302398 | 5.3299143 | Down |
| TAG52:7(22:6) | 0.01358 | -5.308668 | 0.3652456 | Down |
| TAG50:5(20:4) | 0.0073991 | -5.380502 | 0.3492021 | Down |
| TAG54:8(22:6) | 0.0121941 | -5.38789 | 0.5642115 | Down |
| TAG54:7(20:5) | 0.0415561 | -5.391697 | 0.5509651 | Down |

| | | | | |
|---|---|---|---|---|
| TAG46:2(16:0) | 0.0037272 | -5.486814 | 0.7666633 | Down |
| TAG46:1(16:0) | 0.0176031 | -5.514245 | 1.8241614 | Down |
| TAG56:8(22:6) | 0.0048663 | -5.533525 | 3.4781598 | Down |
| TAG45:1(16:0) | 0.0146327 | -5.628581 | 0.2835338 | Down |
| TAG50:5(20:5) | 0.0141082 | -5.635817 | 0.1315363 | Down |
| TAG52:5(20:4) | 0.0092906 | -5.710864 | 2.2186834 | Down |
| TAG55:1(16:0) | 0.0035019 | -5.723715 | 1.7604408 | Down |
| TAG54:7(22:6) | 0.0151904 | -5.745846 | 1.8291821 | Down |
| TAG56:7(22:4) | 0.0049895 | -5.757821 | 1.0546748 | Down |
| TAG56:8(16:0) | 0.0026338 | -5.77446 | 1.7866952 | Down |
| TAG48:2(16:0) | 0.0092274 | -5.78808 | 3.6276013 | Down |
| TAG50:4(20:4) | 0.0019536 | -5.804625 | 0.900717 | Down |
| TAG52:6(22:6) | 0.0052202 | -6.058824 | 1.0899594 | Down |
| TAG44:2(16:0) | 0.0036989 | -6.149027 | 0.6797291 | Down |
| TAG52:6(16:0) | 0.0431222 | -6.210219 | 0.4831912 | Down |
| TAG49:3(16:0) | 0.0081393 | -6.312337 | 0.5696367 | Down |
| TAG54:7(20:4) | 0.021548 | -6.327261 | 2.4651762 | Down |
| TAG52:5(16:0) | 0.0127731 | -6.365432 | 4.0218157 | Down |
| TAG46:3(16:0) | 0.001576 | -6.372825 | 0.4974707 | Down |
| TAG52:6(20:4) | 0.0141209 | -6.594541 | 2.0070726 | Down |
| TAG50:4(16:0) | 0.0175271 | -6.822303 | 2.0677328 | Down |
| TAG48:3(16:0) | 0.0097633 | -7.03226 | 2.5485899 | Down |

**Table S3. Enzymes were reported to regulate PC metabolism in cancers.**

| Gene | Cancer | Article | Function |
|---|---|---|---|
| FADS1 | EC | Molendijk, J [22] et al. | Fatty acid desaturases |
| FASN | LC; EC; OV | Ross, J [23] et al;<br>Mao X [24] et al;<br>Bartolacci C [25] et al | A rate-limiting enzyme in FA synthesis |
| PCYT1A | LC | Yu J [26] et al. | A rate-limiting enzyme in phosphatidylcholine synthesis |
| LPCAT1 | HCC; PC; GC; | Uehara T [27]et al<br>Liu Y [28]et al,<br>Morita Y [29] et al. | A key Enzyme that converts LPC into PC |
| PLD2 | EC; GC; CRC | Cho JH [30] et al;<br>Satio M [31] et al;<br>Boral M [32]et al | An enzyme that hydrolyzes phosphatidylcholine |

*Esophageal cancer:EC; Lung cancer:LC; ovarian cancer:OV; hepatocellular carcinoma:HCC; prostate cancer:PC; cervical cancer,:CC;Breast cancer:BC; lysophosphatidylcholine:LPC; monounsaturated fatty acids:MUFA

**Table S4. The information of differentially expressed gene identified**

| Gene | log2FC | Pvalue | Up/Down |
|---|---|---|---|
| PLOD2 | 4.813491 | 5.39E-27 | Up |
| BCL2L11 | 2.424146 | 3.33E-23 | Up |
| TNC | 1.422043 | 5.59E-21 | UP |
| GTPBP10 | -1.06439 | 8.13E-17 | DOWN |
| SENP3 | 1.038277 | 7.24E-16 | UP |
| SENP3-EIF4A1 | -2.36752 | 1.61E-14 | DOWN |
| ANXA10 | 2.102245 | 4.59E-14 | UP |
| PRNP | 1.756299 | 2.93E-13 | UP |
| EIF2S3B | 2.666661 | 3.23E-13 | UP |
| SCAT8 | -2.30611 | 2.85E-12 | DOWN |
| S100A10 | 1.110734 | 1.20E-08 | UP |
| AC089983.1 | -1.62962 | 6.29E-08 | DOWN |
| YJEFN3 | 1.853149 | 7.08E-08 | UP |
| NDP | 1.629426 | 1.11E-07 | UP |
| P2RX5-TAX1BP3 | 4.555235 | 1.30E-07 | UP |
| PPP1R18 | 1.131466 | 1.63E-07 | UP |
| RBM38 | 1.482955 | 2.06E-07 | UP |
| KCNRG | -2.29777 | 2.93E-07 | DOWN |
| CELSR3 | 1.349182 | 6.12E-07 | UP |
| PPARG | -1.79122 | 8.20E-07 | DOWN |
| ZBTB38 | 1.659297 | 1.63E-06 | UP |
| SAMD12 | -1.5878 | 1.78E-06 | DOWN |
| HBE1 | -1.29409 | 1.82E-06 | DOWN |
| GPC6 | 1.378206 | 2.03E-06 | UP |
| RTL10 | 1.648229 | 2.06E-06 | UP |
| AC092718.3 | -3.56746 | 2.57E-06 | DOWN |
| RASGRP2 | 1.76814 | 2.67E-06 | UP |
| CAMKK2 | 1.198469 | 3.00E-06 | UP |
| CYP2S1 | 1.616001 | 3.51E-06 | UP |
| RN7SL2 | 1.463134 | 3.49E-06 | UP |
| GPRIN3 | 5.215728 | 4.16E-06 | UP |
| AL360181.3 | -1.76199 | 4.54E-06 | DOWN |
| ZNF264 | -2.61226 | 5.66E-06 | DOWN |
| DNAL1 | -1.92395 | 6.93E-06 | DOWN |
| RPL17-C18orf32 | 1.082834 | 7.08E-06 | UP |
| AC139530.2 | #NAME? | 1.29E-05 | DOWN |
| EID3 | -1.28933 | 1.78E-05 | DOWN |
| AC138811.2 | 1.586498 | 2.00E-05 | UP |
| PRH1 | -2.04319 | 2.57E-05 | DOWN |
| GPRASP2 | -3.70482 | 2.73E-05 | DOWN |
| PLA2G4B | 2.246222 | 3.82E-05 | UP |

| | | | |
|---|---|---|---|
| LONRF2 | 2.511714 | 4.42E-05 | UP |
| PDE10A | 2.165504 | 4.83E-05 | UP |
| LOX | 1.060321 | 5.23E-05 | UP |
| STEAP2 | -1.22804 | 7.20E-05 | DOWN |
| C17orf100 | -1.86928 | 7.87E-05 | DOWN |
| MMP19 | -4.30744 | 0.000115 | DOWN |
| MIR210HG | 1.860758 | 0.000139 | UP |
| TAS2R3 | 2.352486 | 0.000145 | UP |
| AQP3 | 1.885656 | 0.000146 | UP |
| MPZL2 | 2.610376 | 0.000152 | UP |
| CACNG7 | 1.323951 | 0.000167 | UP |
| ADAMTS16 | -1.3538 | 0.000171 | DOWN |
| AC090970.2 | 2.355789 | 0.000178 | UP |
| AC026464.2 | 4.763057 | 0.000199 | UP |
| GAS6-AS1 | -1.40505 | 0.000221 | DOWN |
| DYSF | 1.914412 | 0.000225 | UP |
| THBS1 | 1.620344 | 0.000248 | UP |
| TMEM199 | -1.25688 | 0.000256 | DOWN |
| ARX | -10000 | 0.008556132 | DOWN |
| AC018638.8 | -10000 | 0.019626067 | DOWN |
| C7orf57 | -3.581389663 | 0.020679675 | DOWN |
| AC005899.8 | -10000 | 0.0161796 | DOWN |
| AL662884.1 | -6.09946 | 0.000261 | DOWN |

**Table S5. The expression of FASN with clinicopathological characteristics of CRC patients from CRC TMAs.**

| Variables | Low FASN (n=34) | High FASN (n=46) | P value |
|---|---|---|---|
| Sex | | | |
| Female | 14 | 22 | 0.554 |
| Male | 20 | 24 | |
| Age | | | |
| <60 | 19 | 19 | 0.196 |
| ≥60 | 15 | 27 | |
| TNM stage | | | |
| I-II | 20 | 21 | 0.244 |
| III-IV | 14 | 25 | |
| Tumor differentiation | | | |
| Well and moderately differentiated | 32 | 36 | $0.049^{*}$ |
| Poorly differentiated | 2 | 10 | |
| Death | | | |
| + | 5 | 17 | $0.019^{*}$ |
| - | 31 | 29 | |

**Table S6. The expression of PLA2G4B with clinicopathological characteristics of CRC patients from CRC TMAs**

| Variables | Low PLA2G4B (n=39) | High PLA2G4B (n=41) | P value |
|---|---|---|---|
| Sex | | | |
| Female | 17 | 19 | 0.3113 |
| Male | 12 | 22 | |
| Age | | | |
| <60 | 17 | 21 | 0.4946 |
| ≥60 | 22 | 20 | |
| TNM stage | | | |
| I-II | 19 | 22 | 0.6586 |
| III-IV | 20 | 19 | |
| Tumor differentiation | | | |
| Well and moderately differentiated | 29 | 39 | 0.0093* |
| Poorly differentiated | 10 | 2 | |
| Death | | | |
| + | 6 | 16 | 0.0179* |
| - | 33 | 25 | |

**Table S7. The information of lipids internal label**

| Number | Molecular formula | Concentration（ng/ml） | Category |
|---|---|---|---|
| TG 14:0-13:0-14:0-d5.IS | C44H79D5O6 | 25 | TAG |
| TG 14:0-15:1-14:0-d5.IS | C46H81D5O6 | 50 | |
| TG 14:0-17:1-14:0-d5.IS | C48H85D5O6 | 75 | |
| TG 16:0-15:1-16:0-d5.IS | C50H89D5O6 | 100 | |
| TG 16:0-17:1-16:0-d5.IS | C52H93D5O6 | 125 | |
| TG 16:0-19:2-16:0-d5.IS | C54H95D5O6 | 100 | |
| TG 18:1-17:1-18:1-d5.IS | C56H97D5O6 | 75 | |
| TG 18:1-19:2-18:1-d5.IS | C58H99D5O6 | 50 | |
| TG 18:1-21:2-18:1-d5.IS | C60H103D5O6 | 25 | |
| 17:0-14:1 DG-d5.IS | C34H59D5O5 | 25 | DAG&MAG |
| 17:0-16:1 DG-d5.IS | C36H63D5O5 | 50 | |
| 17:0-18:1 DG-d5.IS | C38H67D5O5 | 75 | |
| 17:0-20:3 DG-d5.IS | C40H67D5O5 | 50 | |
| 17:0-22:4 DG-d5.IS | C42H69D5O5 | 25 | |
| 14:1 cholesteryl-d7 ester.IS | C41H63D7O2 | 25 | CE |
| 16:1 cholesteryl-d7 ester.IS | C43H67D7O2 | 50 | |
| 18:1 cholesteryl-d7 ester.IS | C45H71D7O2 | 75 | |
| 20:3 cholesteryl-d7 ester.IS | C47H71D7O2 | 50 | |
| 22:4 cholesteryl-d7 ester.IS | C49H73D7O2 | 25 | |
| C16:1 Ceramide-d7 (d18:1-d7/16:1).IS | C34H58D7NO3 | 75 | CER&HexCer-NS&Hex2Cer&Hex3Cer&CerP&dhCer |
| C18:1 Ceramide-d7 (d18:1-d7/18:1).IS | C36H62D7NO3 | 50 | |
| C20:1 Ceramide-d7 (d18:1-d7/20:1).IS | C38H66D7NO3 | 25 | |
| C22:1 Ceramide-d7 (d18:1-d7/22:1).IS | C40H70D7NO3 | 50 | |

| | | | |
|---|---|---|---|
| C24:1 Ceramide-d7 (d18:1-d7/24:1).IS | C42H74D7NO3 | 75 | |
| 16:1 SM (d18:1/16:1)-d9.IS | C39H68D9N2O6P | 75 | |
| 18:1 SM (d18:1/18:1)-d9.IS | C41H72D9N2O6P | 50 | |
| 20:1 SM (d18:1/20:1)-d9.IS | C43H76D9N2O6P | 25 | SM&Sphinganine&SPH |
| 22:1 SM (d18:1/22:1)-d9.IS | C45H80D9N2O6P | 50 | |
| 24:1 SM (d18:1/24:1)-d9.IS | C47H84D9N2O6P | 75 | |
| 17:0-14:1 PC-d5.IS | C39H71D5NO8P | 50 | |
| 17:0-16:1 PC-d5.IS | C41H75D5NO8P | 100 | |
| 17:0-18:1 PC-d5.IS | C43H79D5NO8P | 150 | PC |
| 17:0-20:3 PC-d5.IS | C45H79D5NO8P | 100 | |
| 17:0-22:4 PC-d5.IS | C47H81D5NO8P | 50 | |
| 15:0 Lyso PC-d5.IS | C23H43D5NO7P | 25 | |
| 17:0 Lyso PC-d5.IS | C25H47D5NO7P | 50 | LPC&LPC-O&LPC-P |
| 19:0 Lyso PC-d5.IS | C27H51D5NO7P | 25 | |
| 17:0-14:1 PE-d5.IS | C36H65D5NO8P | 25 | |
| 17:0-16:1 PE-d5.IS | C38H69D5NO8P | 50 | |
| 17:0-18:1 PE-d5.IS | C40H73D5NO8P | 75 | PE&PE-P |
| 17:0-20:3 PE-d5.IS | C42H73D5NO8P | 50 | |
| 17:0-22:4 PE-d5.IS | C44H75D5NO8P | 25 | |
| 17:0-14:1 PS-d5.IS | C37H64D5NNaO10P | 25 | |
| 17:0-16:1 PS-d5.IS | C39H68D5NNaO10P | 50 | |
| 17:0-18:1 PS-d5.IS | C41H72D5NNaO10P | 75 | PS |
| 17:0-20:3 PS-d5.IS | C43H72D5NNaO10P | 50 | |
| 17:0-22:4 PS-d5.IS | C45H74D5NNaO10P | 25 | |
| 17:0-14:1 PG-d5.IS | C37H65D5NaO10P | 25 | |
| 17:0-16:1 PG-d5.IS | C39H69D5NaO10P | 50 | |
| 17:0-18:1 PG-d5.IS | C41H73D5NaO10P | 75 | PG |
| 17:0-20:3 PG-d5.IS | C43H73D5NaO10P | 50 | |
| 17:0-22:4 PG-d5.IS | C45H75D5NaO10P | 25 | |
| 17:0-14:1 PI-d5.IS | C40H73D5NO13P | 25 | |
| 17:0-16:1 PI-d5.IS | C42H77D5NO13P | 50 | |
| 17:0-18:1 PI-d5.IS | C44H81D5NO13P | 75 | PI |
| 17:0-20:3 PI-d5.IS | C46H81D5NO13P | 50 | |
| 17:0-22:4 PI-d5.IS | C48H83D5NO13P | 25 | |
| 15:0 Lyso PE-d5 | C20H37D5NO7P | 25 | |
| 17:0 Lyso PE-d5 | C22H41D5NO7P | 50 | LPE |
| 19:0 Lyso PE-d5 | C24H45D5NO7P | 25 | |
| 15:0 Lyso PS-d5.IS | C21H36D5NNaO9P | 25 | LPS |

**Table S8. The sequences of the siRNAs**

| siRNAs | Sequence |
|---|---|
| si-FASN-001(human) | GCATCAATGTCCTGCTGAA |
| si-FASN-002(human) | GCGTTGACCTGGTCTTGAA |
| si-FASN-003(human) | GCATGGCTATCTTCCTGAA |
| si-FASN-NC (human) | UUCUCCGAACGUGUCACGUTT<br>ACGUGACACGUUCGGAGAATT |
| si-FASN-001(mouse) | GGAUCAACCUGCUCCUGAATT<br>UUCAGGAGCAGGUUGAUCCTT |
| si-FASN-002(mouse) | CCGUGUGACCGCCAUCUAUTT<br>AUAGAUGGCGGUCACACGGTT |
| si-FASN-003(mouse) | GCAGUGGCCUGGAAUCCAUTT<br>AUGGAUUCCAGGCCACUGCTT |
| si-FASN-NC (mouse) | UUCUCCGAACGUGUCACGUTT<br>ACGUGACACGUUCGGAGAATT |
| si-PLA2G4B-001(human) | GGGTTAGAAAGCTGACAGT |
| si-PLA2G4B-002(human) | GCTGACAGTCCTTGATCCT |
| si-PLA2G4B-003(human) | CCTGGCTCTGGGTTAGAAA |
| si-PLA2G4B-NC (human) | GGCTCTAGAAAAGCCTATGC |

**Table S9. Primers utilized for qRT-PCR and PCR**

| Gene | Forward (5' to 3') | Reverse (5' to 3') |
|---|---|---|
| GAPDH | GGAGCGAGATCCCTCCAAAAT | GGCTGTTGTCATACTTCTCATGG |
| FASN | AAGGACCTGTCTAGGTTTGATGC | TGGCTTCATAGGTGACTTCCA |
| ELF1 | TGTCCAACAGAACGACCTAGT | GGCAGGAAAAATAGCTGGATCAC |
| TCF4 | CAAGCACTGCCGACTACAATA | CCAGGCTGATTCATCCCACTG |
| CEBPB | CTTCAGCCCGTACCTGGAG | GGAGAGGAAGTCGTGGTGC |
| AR | CCAGGGACCATGTTTTGCC | CGAAGACGACAAGATGGACAA |
| ETS1 | GATAGTTGTGATCGCCTCACC | GTCCTCTGAGTCGAAGCTGTC |
| ES1 | CCCACTCAACAGCGTGTCTC | CGTCGATTATCTGAATTTGGCCT |
| CEBPA | GCCAAGAAGTCGGTGGACAA | ATTGTCACTGGTCAGCTCCA |
| YY1 | AAGAGCGGCAAGAAGAGTTAC | CAACCACTGTCTCATGGTCAATA |
| SP1 | GTGGAGGCAACATCATTGCTG | GCCACTGGTACATTGGTCACAT |
| P1 (-1164/-1167) | AGATGGGGTCTCGCTCTGTC | GAGGCTGAGGCAGAATAGTGT |
| P2 (-572/-582) | GTTACTGTCCTCCCCCAGTTC | GTGTGGAGACCAACTCTTTCCT |
| P3 (-1632/-1642) | GGCCTTGACTGATGGAGCAC | CTCAAGCCATGCTGGACCC |

The full length sequence of the promoter is as follows：

>NC_000015.10:41836847-41838846 Homo sapiens chromosome 15, GRCh38.p14 Primary Assembly

CAGGCCCTTCGCTGCACGGTGCGGGCCGGTGAGATGCTCTATCTGCCGGC
TCTGTGGTTCCACCACGTCCAGCAGTCCCAGGGCTGCATCGCAGGTGAAG
AGTTGCCCAGGCCGCCTGGGGAGAGGCCCTGTCAAGGTCCAGCAGGGCCT
CTGGGGGGAGGCTTGGGAGGCTCTAGGTCAGAAGAGGGATCTTCATGCTC
AGATCCCCGTTCTTCCCACAGTGAATTTCTGGTATGACATGGAATACGACC
TCAAGTATAGTTACTTCCAGCTGCTCGACTCCCTCACCAAGGCTTCAGGCC
TTGACTGATGGAGCACTGGTGAACACCACCAAGCACGCCTCGGGGGACGG
AGCCAGCCCCTCCCTGGCCAGGTCAATTCTCGAGAGAGCCTGGAGTGTGC
ATGCTGGCTGCTGGCCCCGGGTCCAGCATGGCTTGAGATCAGCTTTGGAG
GATCTTGGAATGTGGTCATAAGGACTCAAGGTGCCAGGCAGGTCTGGGTG
AGGGTTCTCAGGAAGTTGCCACACAGGTGAGCAGAGTGGGGATCAGGTG
CAGCGGCACCTCTCCCCAGCGCTGTGATGTTGGGCGAGTCACTGCGTCTC
GGGCATTGGTGTCCTGTCAGTAAAGAGATAATAATGGCTGTACCTCGCGG
GGCTGTTGTGGGCTTGGAGATGATGTCTATGAGGACCAGCATGGAGCTGG
CACACAGGACATGTTGAATAAAAGGTAGCTGTGAGTCGTATGTCCTTTTTT
TTTTTTTTTAAGATGGGGTCTCGCTCTGTCACCCAGGCTGGAGTGCAGTG
GTGTGATGTCAGCTCACTGCAAGCTCCGCCTCCCAGGTTCACACTATTCTG
CCTCAGCCTCCCAAGTAGCTGGGACTACAGGTGCGTGCCACCATGCCCGG
CTAATTTTTTTGTATTTTTAGTAGAGACGGGGTTTCACCGTGTTAGCCAGT
ATGGTCTTGATCTCCTGACCTCGTGATCCACCTGCCTCGGCCTCCCAAAAG
TGCTGGGATTACAGGTGTGAGCTGCTGCGCCTGGCTTATGAGTCGTATGTT
CTGATCCTCCCTCTTGAAGTTGCCTTCTGTGGTCTAAGGAGGGCCTGAAGG
TTCAGGTAAAAACTTCAGGGTGACCTTCACTGGGGGTGAGGGCTGGATCC
CAGCCTGGGCCCAAAGAGCCGTCAGCTGCCCAAGTCCCGCTGTCCATGAG
AGTCACCGCAGCCCCTCCCTGGGACAAGCAAGCAGACCTGAGTCTTGTAG
CTCTCTGGTCCGGACCTCTTTGCCCAGGACCTTGAGAGCTATTCCTAGCTC
TCCTATGGTTACTGTCCTCCCCCAGTTCAGGGGCAGCAGGTGGGACCTGGT
GCCCTGGGGATAACCCCTGTTTCTCCCATAACAGGCACAGGCAGGAAGGG
ACGGAAGCCCCCGCCTCTCCTGGGGCTGTCCCTCTGAGGAAAGAGTTGGT
CTCCACACGCTGACCCCCCCACAAACCATGCCCTGGAGGCAGAAGAACCC
CCTGCCCCTGAGTGCCAACCCACAGGCCTCATCCCTGGCCACTCAGCACCT
AGCTTTGAAGGGCTGTTTTATGTGACAGCCACTCCCCTGCCTGTCGTGAGG
GGGCCCGGGTGTTCATCTCAGATTGATGGATCCCTGCCATCAAGACTGGG
CATTCCTGTCCAACAGGTGCCAGAGTTGCGAAAGGCCTGTGACAGGGAAC
TCCACTCTTCCCTTGGCTGCTGTTCTGGGACTCACCCCTGCTTTCCTTCTGC
TCAGCCCCTGGCAGCAAGCTCTCCAGGCTGGGATTGCAGGGCTGGGTGGG
GCAGGCCCAGCTGGTAAAGGCTGGCGAGTGCCACAGAGGTATCAGGAGC
TCTAGTATAGGCTTAGGGTGCCTCATTTCCTGGACAGGTGGCTGGTTCAGG
AGTGGGTGTGGAGCTTAGGTGGAGCAGAGGCGGCGGTAGGAGGGACTT
GGGACCAATTGGGACATCACATCCCTGGCTCTGGGTT
