# Supplementary figures and images for: FASN promotes lipid metabolism and progression in colorectal cancer via the SP1/PLA2G4B axis
Source: Cell Death Discov. 2025 Mar 28;11:122. doi: 10.1038/s41420-025-02409-9 (PMC11950308; doi:10.1038/s41420-025-02409-9)

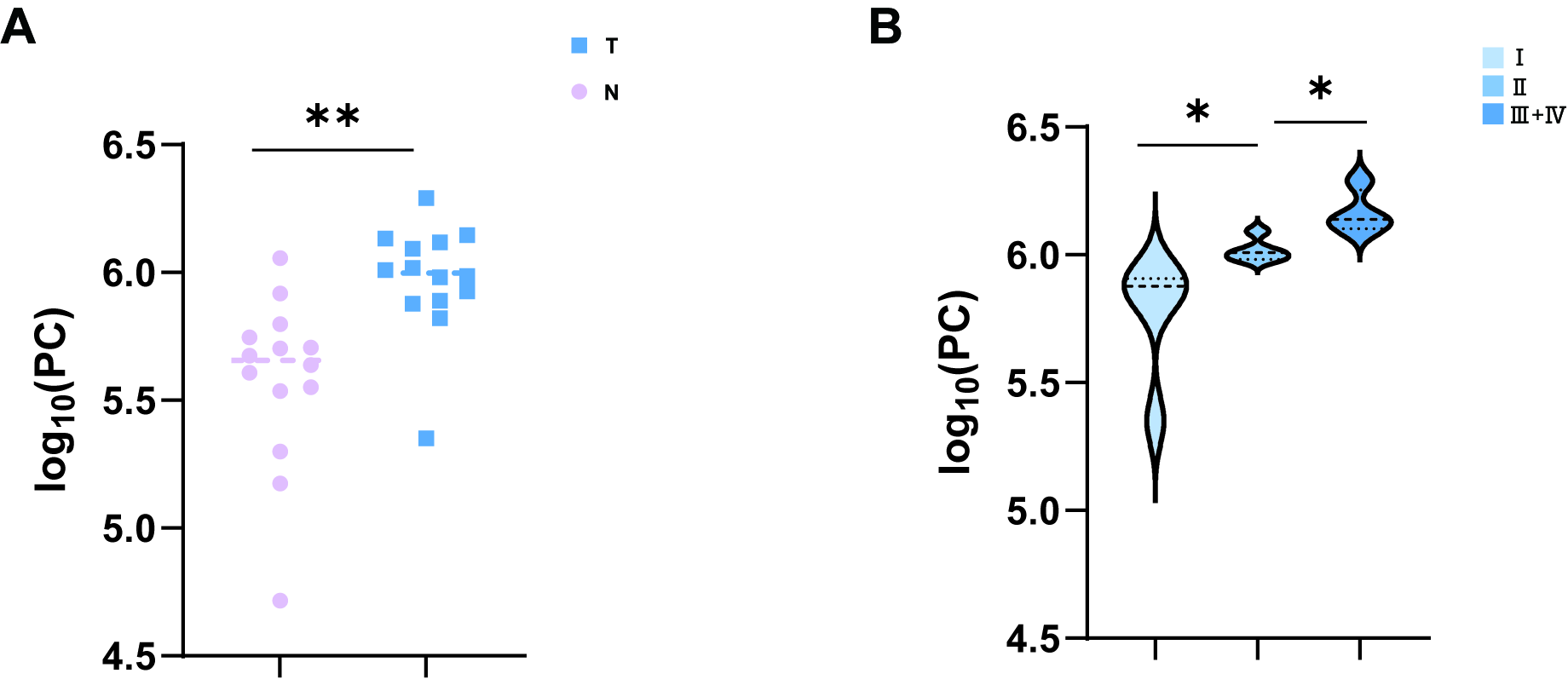

Supplement: Supplementary file 2 — Figure S1 [file 41420_2025_2409_MOESM2_ESM.tif]

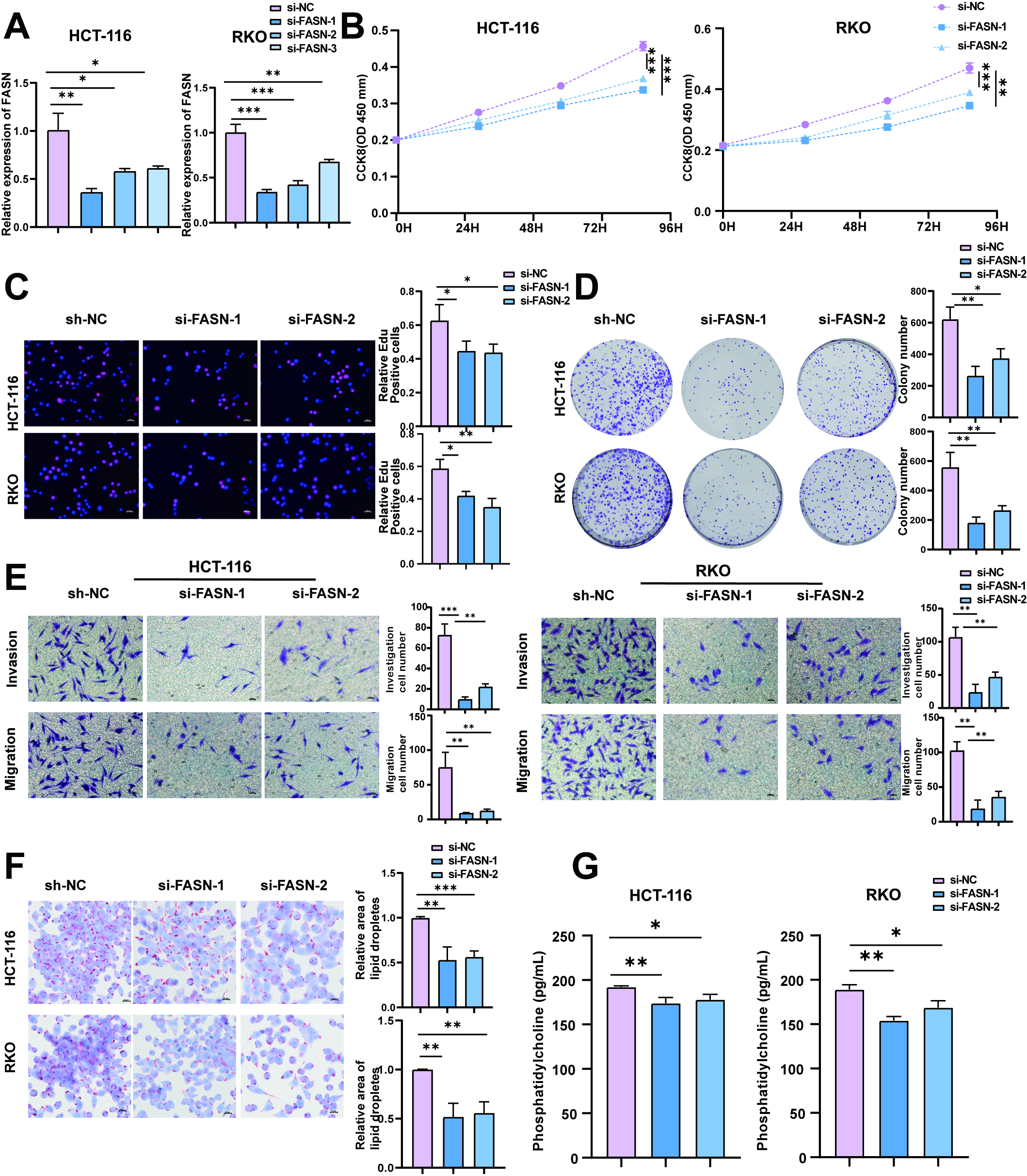

Supplement: Supplementary file 3 — Figure S2 [file 41420_2025_2409_MOESM3_ESM.tif]

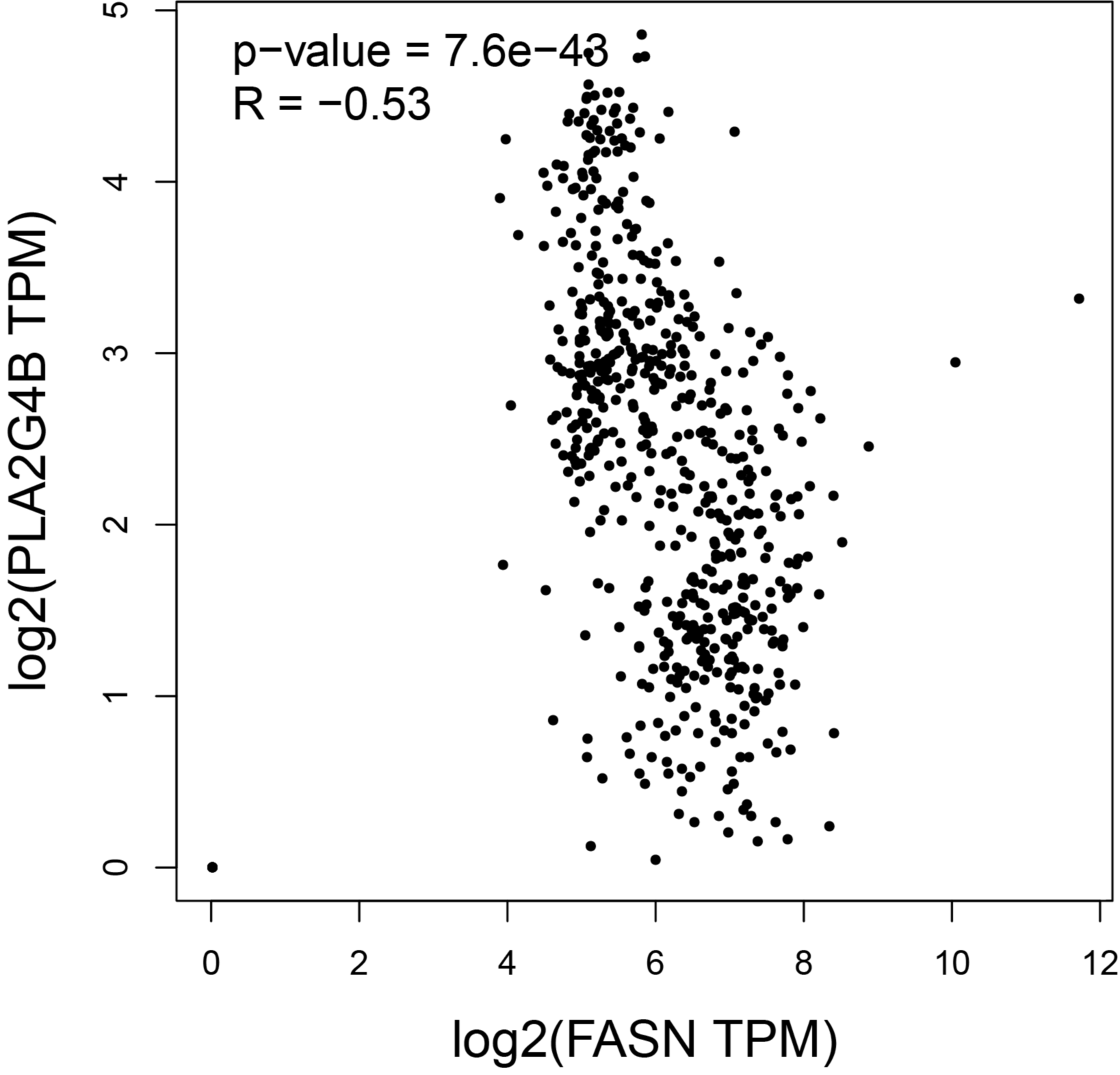

Supplement: Supplementary file 4 — Figure S3 [file 41420_2025_2409_MOESM4_ESM.tif]

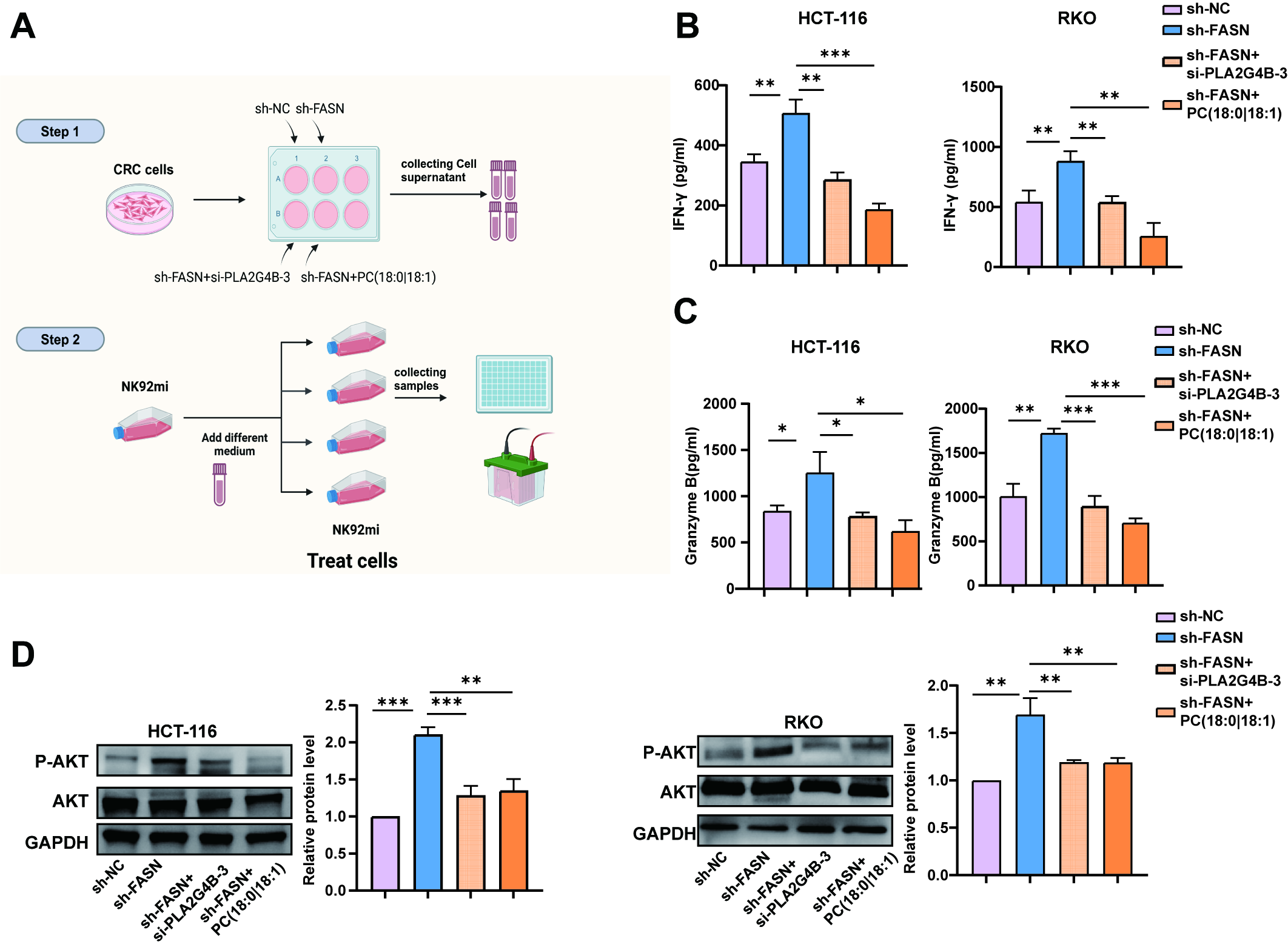

Supplement: Supplementary file 5 — Figure S4 [file 41420_2025_2409_MOESM5_ESM.tif]
