# Supplementary material for: FASN promotes lipid metabolism and progression in colorectal cancer via the SP1/PLA2G4B axis
Source: Cell Death Discov. 2025 Mar 28;11:122. doi: 10.1038/s41420-025-02409-9 (PMC11950308; doi:10.1038/s41420-025-02409-9)
Supplement: Supplementary file 6 — Supplement Material—Figure legend [file 41420_2025_2409_MOESM6_ESM.docx]

**Table legend**

**Table S1. The clinical features of 14 CRC patients, used for Lipidomic analysis, were shown.**

**Table S2. The information of 547 types of differentially expressed lipid identified**

**Table S3. Enzymes were reported to regulate PC metabolism in cancers.**

**Table S4. The information of differentially expressed gene identified**

**Table S5. The expression of FASN with clinicopathological characteristics of CRC patients from CRC TMAs.**

**Table S6. The expression of PLA2G4B with clinicopathological characteristics of CRC patients from CRC TMAs**

**Table S7.** **The information of lipids internal label**

**Table S8. The sequences of the siRNAs**

**Table S9. Primers utilized for qRT-PCR and PCR**

**Figure legend**

**Figure S1** A Compared to adjacent normal tissues (N), the total PC levels in tumour tissue (T) from 14 CRC patients were detected.

B The total level of PC in tumour tissue of 14 patients with CRC at different stages.

The data are presented as the means with standard deviations (SDs), and statistical significance was assessed via Student's t test. Significance levels are shown as **P< 0.01, and ***P< 0.001.

**Figure S2. FASN knockdown inhibits the proliferation, migration, and invasion of CRC cells and synthesis of PC**

A The mRNA expression level of FASN in HCT-116 and RKO cells was examined by RT-qPCR following transfection with si-FASN-1, si-FASN-2, or si-FASN-3.

B The proliferation of HCT-116 and RKO cells was assessed after transfection with si-FASN-1 and si-FASN-2 using a CCK-8 assay

C EdU analysis was performed to measure the proliferative ability of HCT-116 and RKO cells transfected with si-FASN-1 and si-FASN-2. Representative images are presented. Scale bar, 100 µm. The percentage of EdU-positive cells was statistically analysed and is shown in the bar graph.

D A colony formation assay was conducted to evaluate the proliferative ability of HCT-116 and RKO cells transfected with si-FASN-1 and si-FASN-2.

E Transwell migration and invasion assays were performed to examine the migratory and invasive capacities of HCT-116 and RKO cells transfected with si-FASN-1 and si-FASN-2.

F Representative images of intracellular lipid droplets in HCT-116 and RKO cells transfected with si-FASN-1 and si-FASN-2 stained with Oil Red O.

G The expression levels of PC in the cell supernatants of HCT-116 and RKO cells transfected with si-FASN-1 and si-FASN-2 were quantified via ELISA.

The experiments were conducted in triplicate. The data are presented as the means with standard deviations (SDs), and statistical significance was assessed via Student's t test. Significance levels are shown as *P< 0.05, **P< 0.01, and ***P< 0.001.

**Figure S3 TCGA databased revealed an inverse correlation between FASN and PLA2G4B mRNA expression in CRC.**

**Figure S4** FASN suppresses the killing ability of NK cells via AKT pathway in a PC-dependent manner

**A** Schematic diagram of follow-up experiments after treatment of NK-92mi cells using conditioned media from CRC cells treated with sh-NC, sh-FASN, sh-FASN+si-PLA2G4B-3, or sh-FASN+PC (18:0|18:1).

**B-C** The expression level of IFN-γ (B) and granzyme B (C) in the cell supernatants of NK-92mi cells treated with conditioned medium from CRC cells treated with sh-NC, sh-FASN, sh-FASN+si-PLA2G4B-3, or sh-FASN+PC (18:0|18:1) was quantified via ELISA

**D** Western blot analysis was performed to evaluate the expression of AKT and p-AKT in NK-92mi cells treated with conditioned medium from CRC cells treated with sh-NC, sh-FASN, sh-FASN+si-PLA2G4B-3, or sh-FASN+PC (18:0|18:1). GAPDH was used as the control.

The data are presented as the means with standard deviations (SDs), and statistical significance was assessed via Student's t test. Significance levels are shown as **P< 0.01, and ***P< 0.001.
