# Supplementary material for: FASN promotes lipid metabolism and progression in colorectal cancer via the SP1/PLA2G4B axis
Source: Cell Death Discov. 2025 Mar 28;11:122. doi: 10.1038/s41420-025-02409-9 (PMC11950308; doi:10.1038/s41420-025-02409-9)

Figure1F

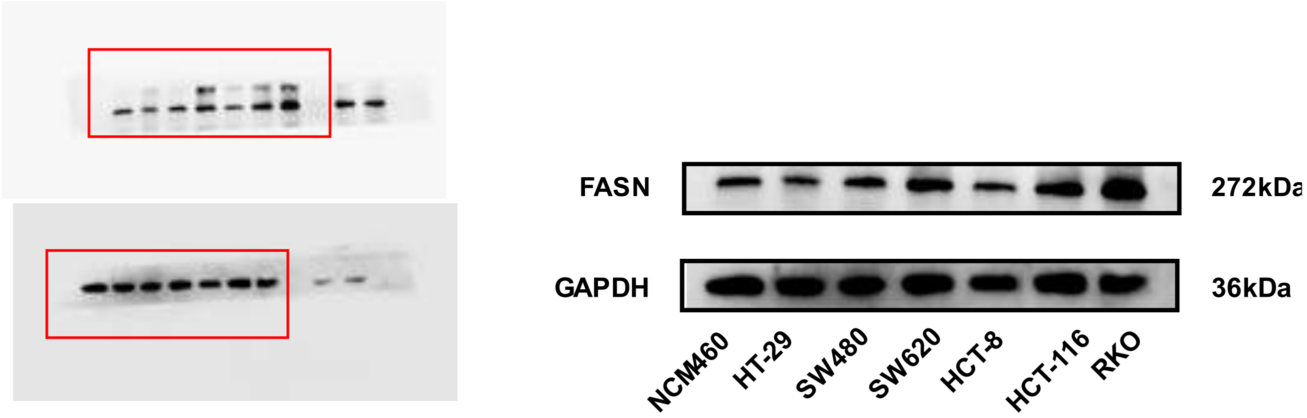

Figure 2A

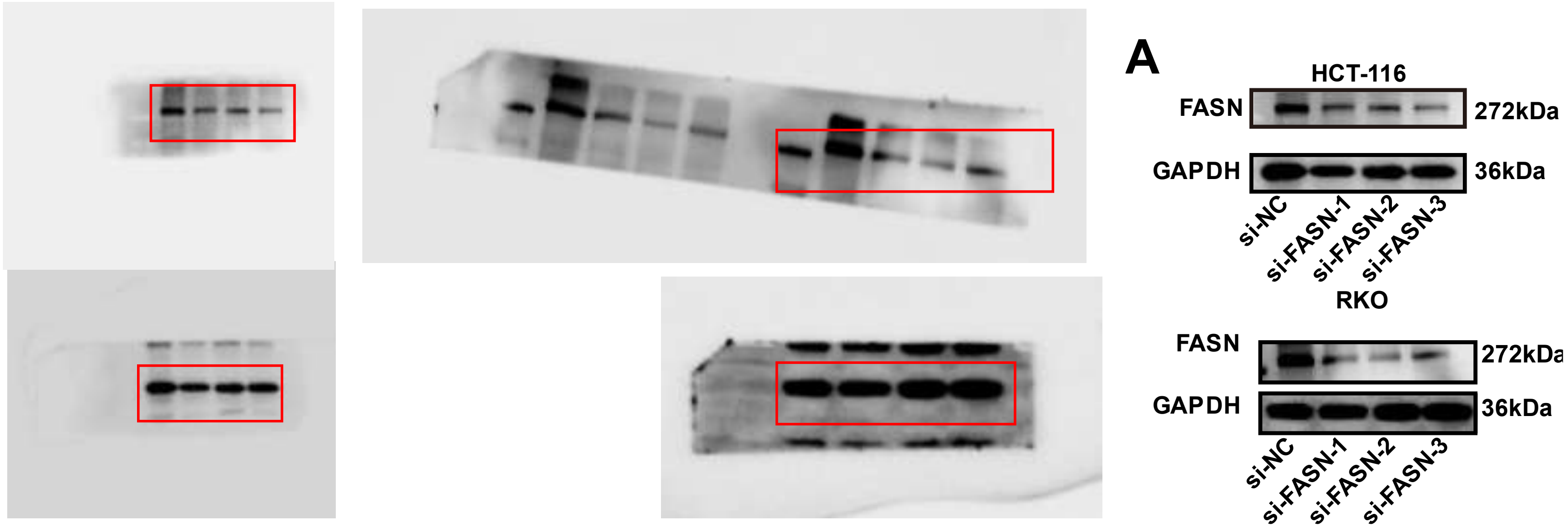

Figure 2B

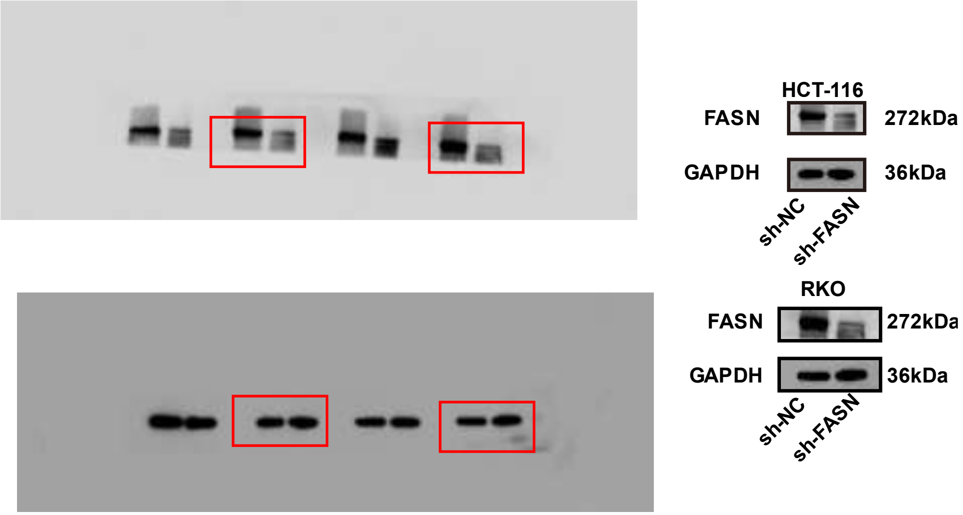

Figure 4A

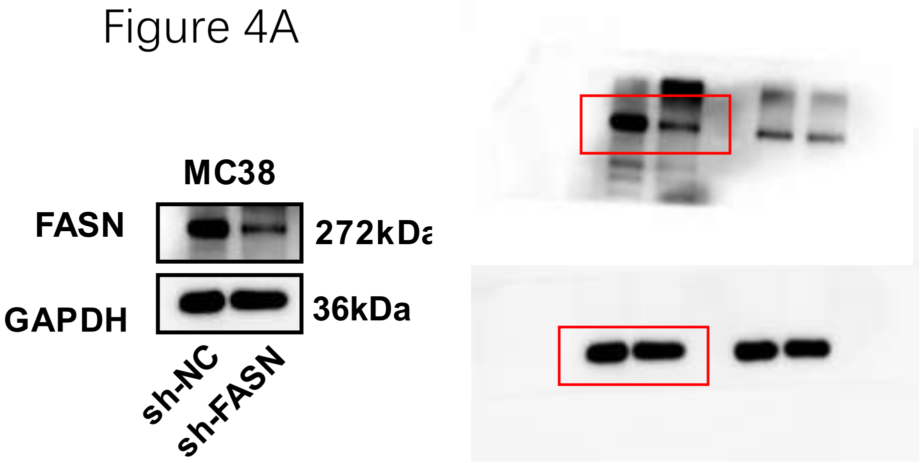

Figure 3A

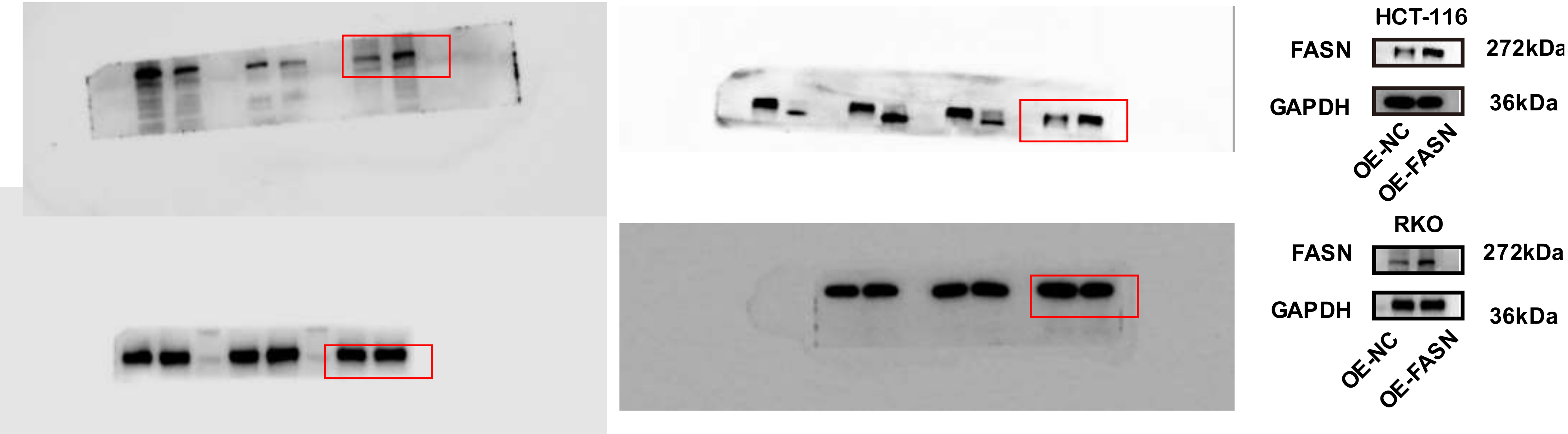

Figure 5B

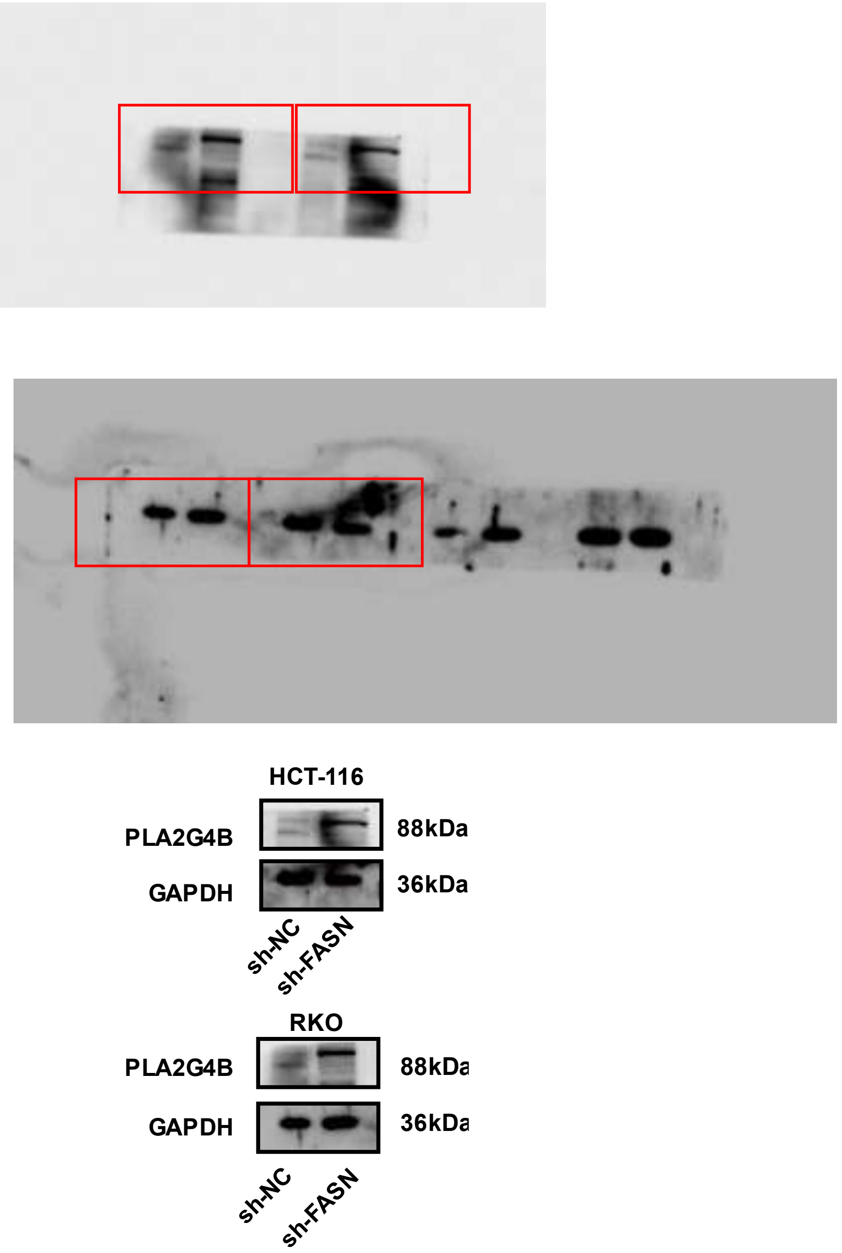

Figure 5C

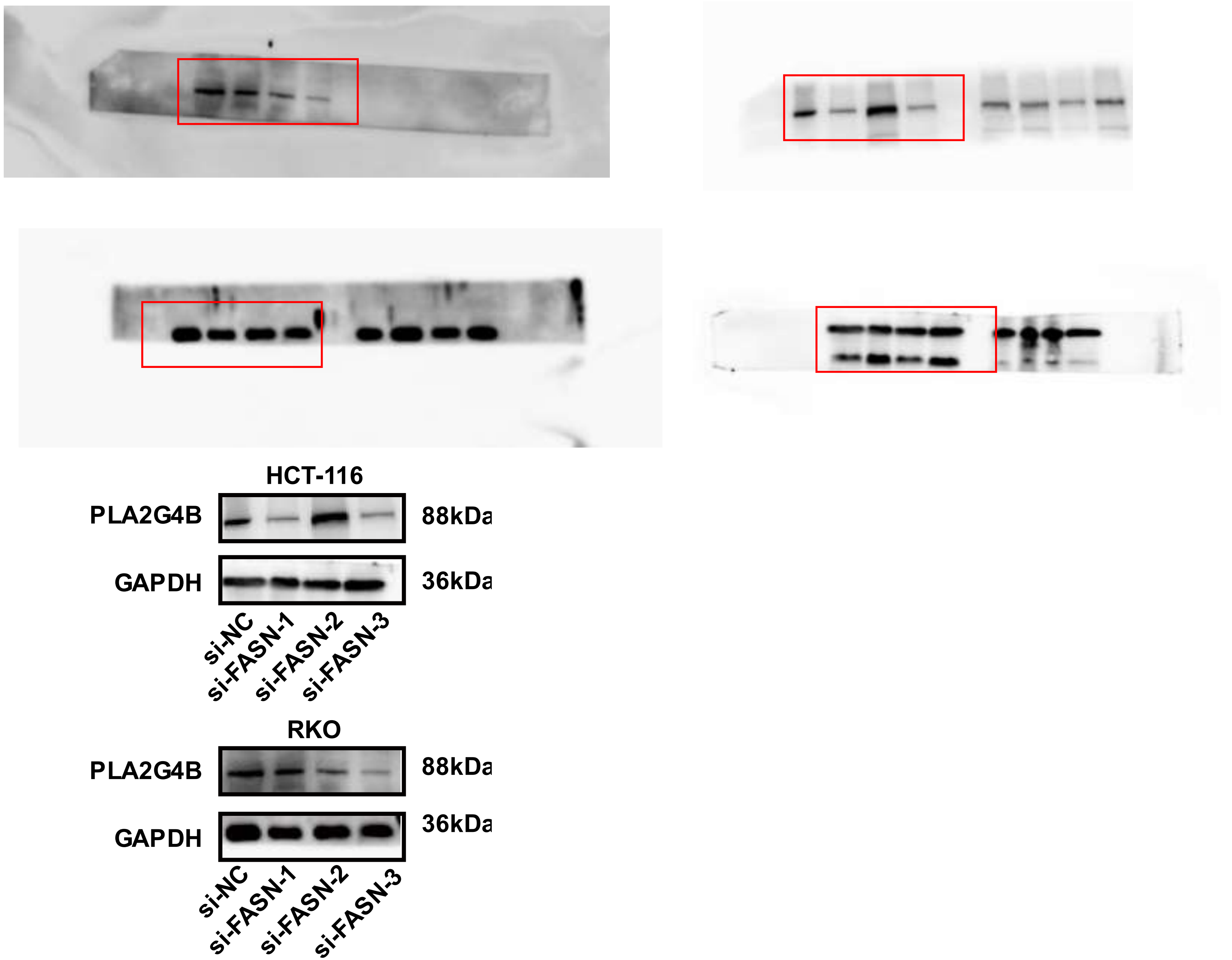

Figure 5d

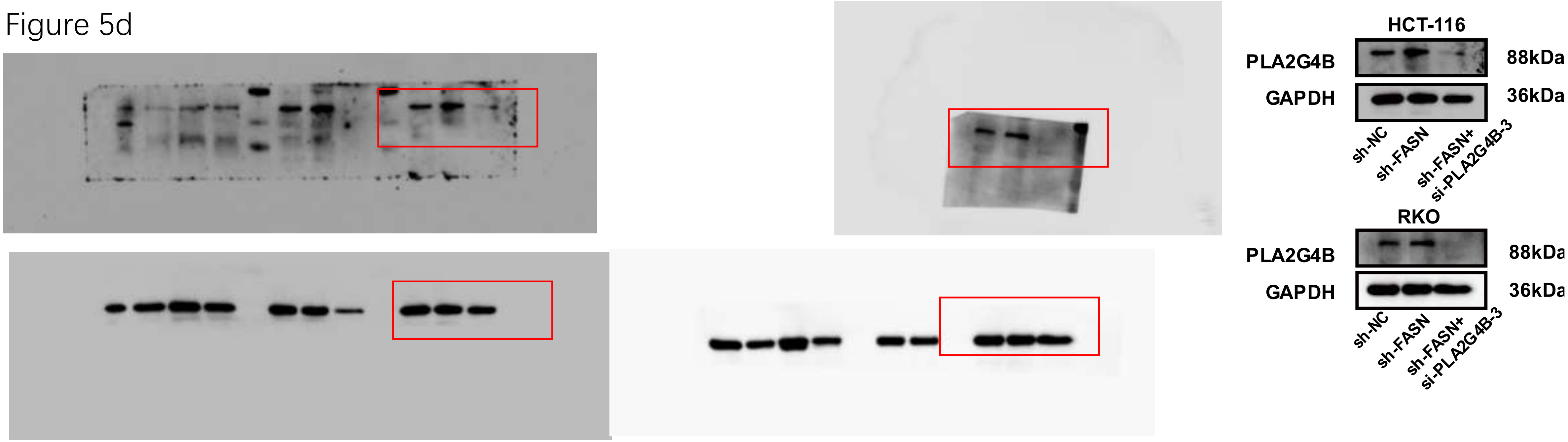

Figure 6C

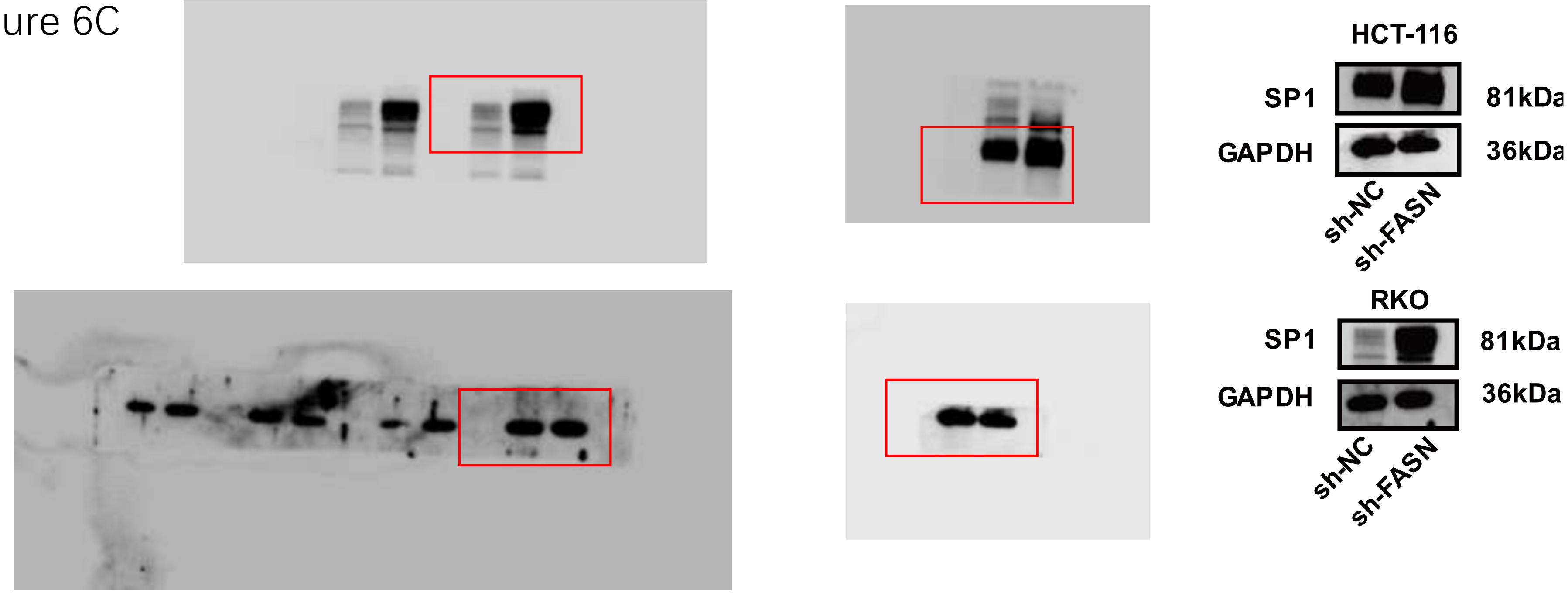

Figure 6I

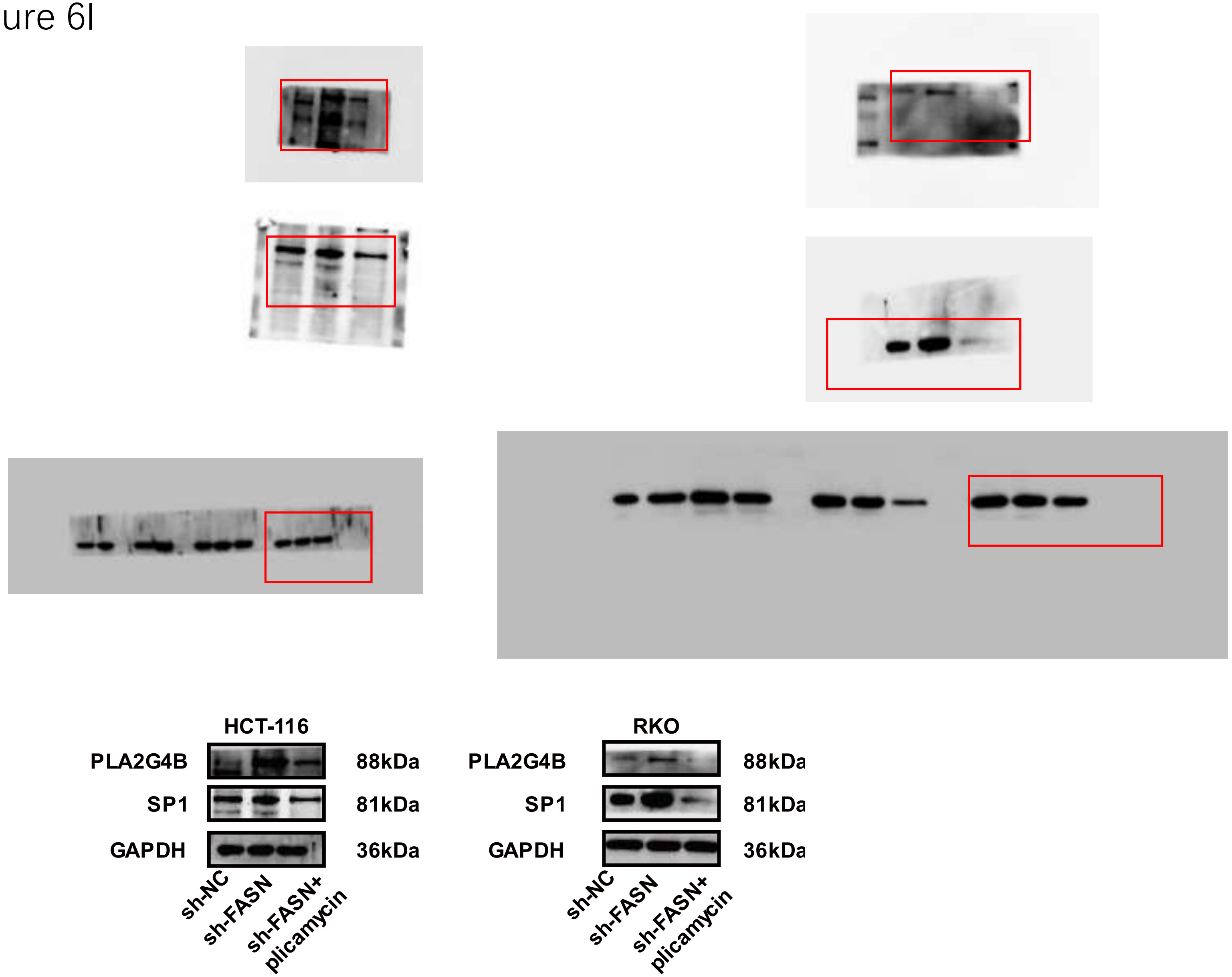
HCT-116
PLA2G4B
SP1
GAPDH
88kDa
81kDa
36kDa
sh-NC
sh-FASN
sh-FASN+ plicamycin
RKO
PLA2G4B
SP1
GAPDH
88kDa
81kDa
36kDa
sh-NC
sh-FASN
sh-FASN+ plicamycin

P-AKT

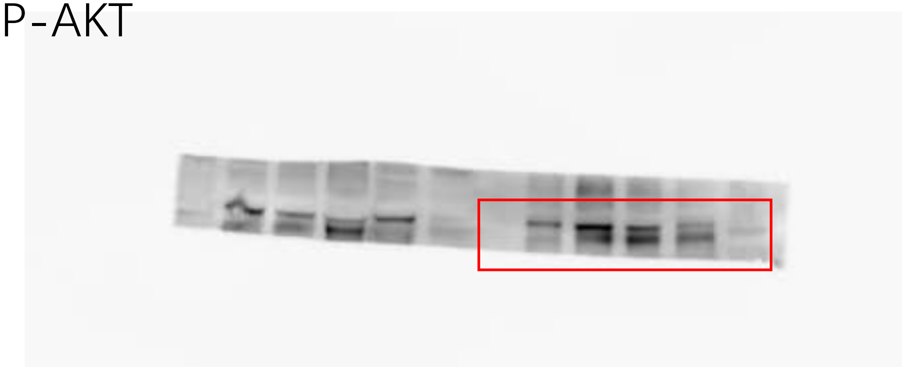

AKT

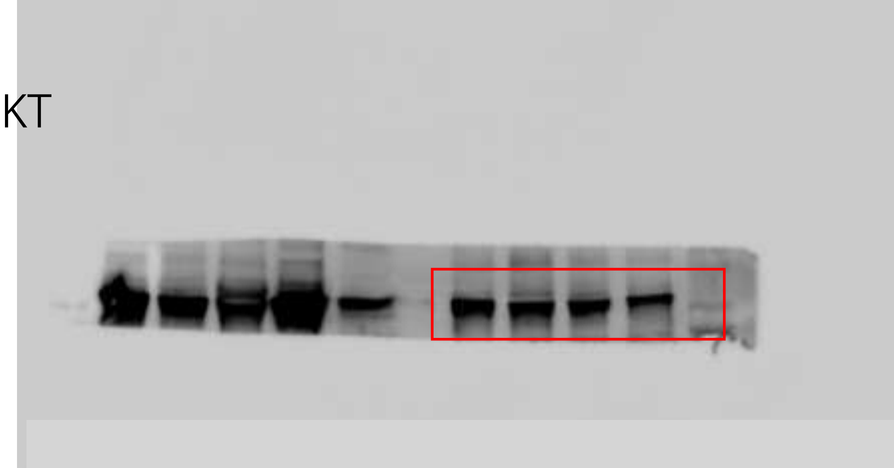

GAPDH

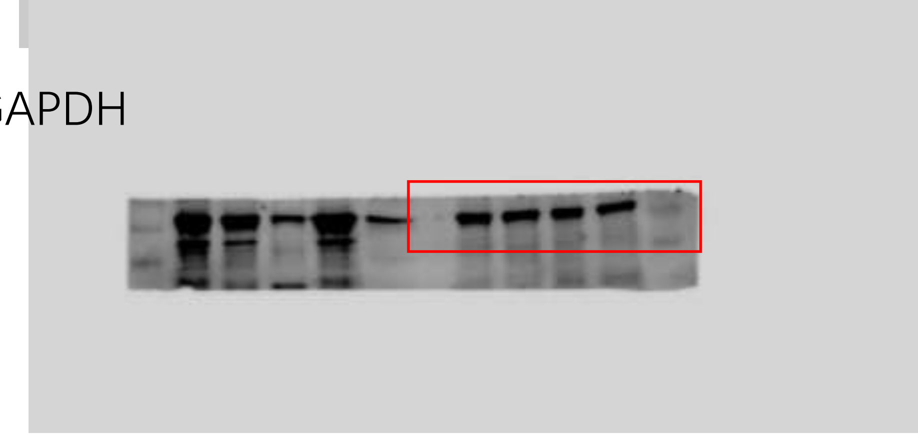

Figure S4D

D

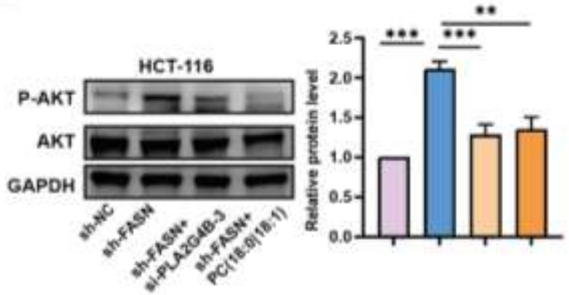

P-AKT

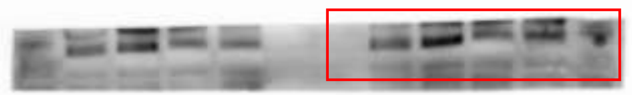

Figure S4D

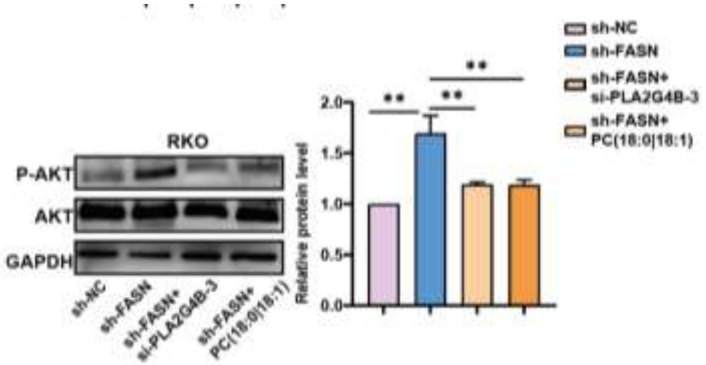

AKT

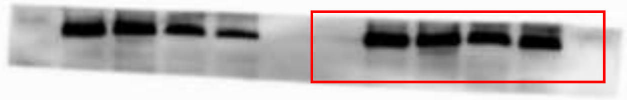

GAPDH

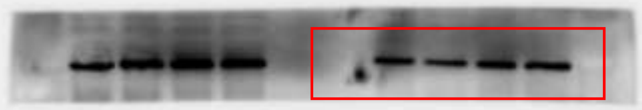

Supplement: Supplementary file 7 — Original Data File [file 41420_2025_2409_MOESM7_ESM.pdf]
